# SGF29 nuclear condensates reinforce cellular aging

**DOI:** 10.1038/s41421-023-00602-7

**Published:** 2023-11-07

**Authors:** Kaowen Yan, Qianzhao Ji, Dongxin Zhao, Mingheng Li, Xiaoyan Sun, Zehua Wang, Xiaoqian Liu, Zunpeng Liu, Hongyu Li, Yingjie Ding, Si Wang, Juan Carlos Izpisua Belmonte, Jing Qu, Weiqi Zhang, Guang-Hui Liu

**Affiliations:** 1 State Key Laboratory of Membrane Biology, Institute of Zoology, Chinese Academy of Sciences, Beijing, China; 2 University of Chinese Academy of Sciences, Beijing, China; 3 Institute for Stem Cell and Regeneration, Chinese Academy of Sciences, Beijing, China; 4 Beijing Institute for Stem Cell and Regenerative Medicine, Beijing, China; 5 Shanghai Institute of Materia Medica, Chinese Academy of Sciences, Shanghai, China; 6 State Key Laboratory of Stem Cell and Reproductive Biology, Institute of Zoology, Chinese Academy of Sciences, Beijing, China; 7 CAS Key Laboratory of Genomic and Precision Medicine, Beijing Institute of Genomics, Chinese Academy of Sciences and China National Center for Bioinformation, Beijing, China; 8 National Laboratory of Biomacromolecules, CAS Center for Excellence in Biomacromolecules, Institute of Biophysics, Chinese Academy of Sciences, Beijing, China; 9 Advanced Innovation Center for Human Brain Protection, and National Clinical Research Center for Geriatric Disorders, Xuanwu Hospital Capital Medical University, Beijing, China; 10 Aging Translational Medicine Center, International Center for Aging and Cancer, Xuanwu Hospital, Capital Medical University, Beijing, China; 11 Altos Labs, Inc., San Diego, CA USA; 12 Sino-Danish College, University of Chinese Academy of Sciences, Beijing, China

**Keywords:** Cell biology, Senescence

## Abstract

Phase separation, a biophysical segregation of subcellular milieus referred as condensates, is known to regulate transcription, but its impacts on physiological processes are less clear. Here, we demonstrate the formation of liquid-like nuclear condensates by SGF29, a component of the SAGA transcriptional coactivator complex, during cellular senescence in human mesenchymal progenitor cells (hMPCs) and fibroblasts. The Arg 207 within the intrinsically disordered region is identified as the key amino acid residue for SGF29 to form phase separation. Through epigenomic and transcriptomic analysis, our data indicated that both condensate formation and H3K4me3 binding of SGF29 are essential for establishing its precise chromatin location, recruiting transcriptional factors and co-activators to target specific genomic loci, and initiating the expression of genes associated with senescence, such as *CDKN1A*. The formation of SGF29 condensates alone, however, may not be sufficient to drive H3K4me3 binding or achieve transactivation functions. Our study establishes a link between phase separation and aging regulation, highlighting nuclear condensates as a functional unit that facilitate shaping transcriptional landscapes in aging.

## Introduction

Aging is a physiological process associated with increased risk of a wide range of age-related diseases^1–3^. As aging progresses, tissues and organs undergo a variety of structural and functional changes. These are reflected at the cellular and molecular level as senescence, loss of proteostasis, and epigenetic alterations^4–7^. Despite tremendous efforts are devoted to interpret the proteostasis collapse with age, little is known about how the crowded macromolecule interaction environment reshapes in senescent cells. Indeed, the mechanisms underlying the aging-related aberrant organization of chromatin and transcriptional apparatus remain a mystery under the conventional models of transcriptional control. Recently, liquid–liquid phase separation (LLPS), a process which creates membrane-less compartments in cells^8–15^, has provided a new paradigm of spatiotemporal control of gene transcription across a broad spectrum of biological contexts, including embryonic development and cancer^16–31^. In this regard, understanding how phase-separated condensates facilitate the partitioning of essential molecular constituents and, consequently, govern the transcription of age-associated genes holds significant potential for offering new insights into the molecular foundation of aging.

Recent studies have provided compelling evidence indicating that chromatin has the ability to undergo phase separation, resulting in the formation of condensates with distinct transcriptional properties. Notable examples of these condensates include complexes such as Pol II, BRD4 and MED1, associated with active transcription, as well as complexes like HP1 and CBX2, associated with transcriptional repression^10,32–35^. These findings underscore the intricate interplay among epigenetic states, gene transcription, and phase separation, highlighting their tightly intertwined regulatory dynamics. Within this framework, Spt-Ada-Gcn5 acetyltransferase (SAGA) complex stands out as an evolutionarily conserved transcription coactivator complex that possesses histone acetyltransferase (HAT) activity, playing central roles in transcription initiation and histone modification^36–42^. Notably, a specific subunit of SAGA complex, known as SAGA-associated factor 29 (SGF29), serves as a recognition and binding module for H3K4me2/3, facilitating the recruitment of the SAGA complex to specific genomic sites^43,44^. SGF29 has been reported to mediate proto-oncogene MYC and MYC-regulated gene sets, and to be essential for malignant transformation of hepatocellular carcinoma^45^ and acute myeloid leukemia^46^. However, whether SGF29 physically undergoes phase-separation, and whether it exerts an epigenetic role in governing important biological functions, such as cellular senescence, is still unknown.

In this study, we discovered that senescence-associated formation of SGF29 intranuclear condensates reinforced the transcription program of cellular aging. In addition to facilitating the binding of H3K4me3 by SAGA complex, the condensates formed by SGF29 provide a specialized compartment that accommodates key elements of the transcriptional machinery, including the transcription factor SP1, transcriptional coactivator MED4 and Pol II. These SGF29 condensates play a crucial role in activating gene transcription associated with senescence, thereby mediating cellular senescence. Our results provide valuable insights into the molecular properties of SGF29, highlighting its roles in phase separation states necessary for appropriate chromatin binding, coordinating interactions with various transcriptional factors and cofactors, and ultimately facilitating efficient and targeted transcriptional activation. This mechanism involving SGF29 represents a novel role for phase separation in the regulation of aging processes.

## Results

### Senescence-associated SGF29 nuclear condensates mediate hMPC aging

**Fig. 1:**
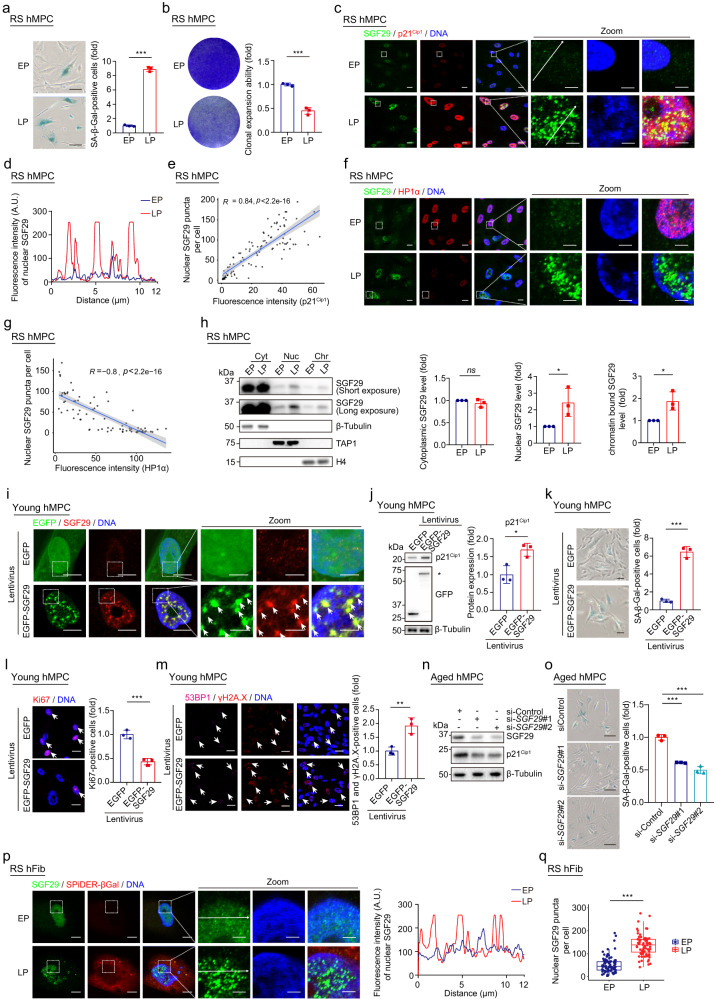
SGF29 forms nuclear condensate in senescent cells and its overexpression promotes cellular aging in hMPCs. **a** SA-β-Gal staining of WT hMPCs at early passage (EP, P5) and late passage (LP, P16). Left, representative images of SA-β-Gal staining. Scale bars, 50 μm. Right, quantitation of the relative percentages of SA-β-Gal-positive cells. Data are presented as the mean ± SEM. *n* = 3 biological replicates. Over 100 cells were quantified in each replicate. ***P < 0.001 (*t*-test). **b** Clonal expansion assay in WT hMPCs at EP (P5) and LP (P16). Left, representative images of crystal violet staining. Right, quantification of the relative clonal expansion ability of EP and LP hMPCs. Data are presented as the mean ± SEM. *n* = 3 biological replicates. ***P < 0.001 (*t-*test). **c** Immunofluorescence staining of p21^Cip1^ and SGF29 in hMPCs at EP (P5) and LP (P16). Scale bars, 10 μm and 2.5 μm (zoomed-in image). **d** Quantification of the fluorescence intensity along the inset arrows in (**c**) following the arrow direction. **e** Correlation between the fluorescence intensity of p21^Cip1^ and the number of nuclear SGF29 puncta. *n* = 100 hMPCs. Each dot represents one cell. The SGF29 condensate with a size greater than 0.196 μm^2^ (area) was quantified as a SGF29 punctum. **f** Immunofluorescence staining of HP1α and SGF29 in hMPCs at EP (P5) and LP (P16). Scale bars, 10 μm and 2.5 μm (zoomed-in image). **g** Correlation between the fluorescence intensity of HP1α and the number of nuclear SGF29 puncta. *n* = 100 hMPCs. Each dot represents one cell. The SGF29 condensate with a size greater than 0.196 μm^2^ (area) was quantified as a SGF29 punctum. **h** Western blot analysis of the abundance of SGF29 in the cytoplasmic (Cyt), nuclear soluble (Nuc) and chromatin-associated (Chr) fractions in hMPCs at EP (P5) and LP (P16). β-Tubulin, TAP1, and H4 were used as the loading control for indicated fraction, respectively. Left, representative images of western blotting. Right, quantification of the relative protein levels of SGF29 in indicated fractions. *n* =3 independent experiments. Data are presented as the means ± SEM. ns, not significant; *P < 0.05 (*t*-test). **i** Immunofluorescence staining of SGF29 in EP WT hMPCs (P5, young hMPCs) transduced with lentiviruses expressing either EGFP or EGFP-SGF29. The white arrowheads denote the SGF29 puncta. Scale bars, 10 μm and 2.5 μm (zoomed-in image). **j** Western blot analysis of p21^Cip1^ in EP WT hMPCs (P5, young hMPCs) transduced with lentiviruses expressing either EGFP or EGFP-SGF29. Left, representative images of western blotting. The band of exogenous EGFP-SGF29 protein is marked with *. β-Tubulin was used as the loading control. Right, quantitation of the relative protein levels of p21^Cip1^. Data are presented as the means ± SEM. *n* = 3 biological replicates. *P < 0.05 (*t-*test). **k** SA-β-Gal staining of EP WT hMPCs (P5, young hMPCs) transduced with lentiviruses expressing either EGFP or EGFP-SGF29. Left, representative images of SA-β-Gal staining. Scale bars, 20 μm. Right, quantitation of the relative percentages of SA-β-Gal-positive cells. Data are presented as the mean ± SEM. *n* = 3 biological replicates. Over 100 cells were quantified in each replicate. ***P < 0.001 (*t-*test). **l** Immunofluorescence staining of Ki67 in EP WT hMPCs (P5, young hMPCs) transduced with lentiviruses expressing either EGFP or EGFP-SGF29. Left, representative images of Ki67 immunofluorescence. The white arrowheads denote the Ki67-positive cells. Scale bars, 20 μm. Right, quantification of the relative percentages of Ki67-positive cells. Data are presented as the mean ± SEM. *n* = 3 biological replicates. Over 100 cells were quantified in each replicate. ***P < 0.001 (*t-*test). **m** Immunofluorescence staining of γH2A.X and 53BP1 in EP WT hMPCs (P5, young hMPCs) transduced with lentiviruses expressing either EGFP or EGFP-SGF29. Left, representative images of γH2A.X and 53BP1 immunofluorescence. The white arrowheads denote the γH2A.X and 53BP1-positive cells. Scale bars, 20 μm. Right, quantification of the relative percentages of γH2A.X and 53BP1-positive cells. Data are presented as the mean ± SEM. *n* = 3 biological replicates. Over 100 cells were quantified in each replicate. **P < 0.01 (*t-*test). **n** Western blot analysis of SGF29 in LP WT hMPCs (P16, aged hMPCs) after treatment with si-Control or si-*SGF29*. β-Tubulin was used as the loading control. **o** SA-β-Gal staining of LP WT hMPCs (P16, aged hMPCs) after treatment with si-Control or si-*SGF29*. Left, representative images of SA-β-Gal staining. Scale bars, 50 μm. Right, quantitation of the relative percentages of SA-β-Gal-positive cells. Data are presented as the mean ± SEM. *n* = 3 biological replicates. Over 100 cells were quantified in each replicate; ***P < 0.001 (*t-*test). **p** Immunofluorescence staining of SPIDER-βGal and SGF29 in human fibroblasts (hFib) at EP (P13) and LP (P23). Left, representative images of SPIDER-βGal and SGF29 immunofluorescence. Right, quantification of the fluorescence intensity along the line embedded in the zoomed-in images following the arrow direction. Scale bars, 10 μm and 2.5 μm (zoomed-in image). **q** Quantification of the number of SGF29 puncta in human fibroblasts at EP (P13) and LP (P23) in Fig. 1p. *n* = 80 hFib cells. Data are shown as means ± SEM. ***P < 0.001 (*t-*test).

To investigate the potential of SAGA complex members to form punctate structure during cellular senescence, we referenced to PhaSepDB, a database of liquid–liquid phase separation-related proteins^47^ and found that six members of the SAGA complex^41^, namely SGF29, TAF5L, SUPT20H, ATXN7L2, ATXN7L3, and SF3B3, were predicted to have the potential for phase separation (Supplementary Fig. S1a). Subsequently, we employed replicative-senescent human mesenchymal progenitor cells (RS hMPCs), a cellular model of senescence^48^. Compared to early passage (EP) counterparts, late passage (LP) hMPCs exhibit typical senescent phenotypes, including an increase in the fraction of cells with senescence-associated β-galactosidase (SA-β-Gal) activity (Fig. 1a), upregulation of the senescent marker p21^Cip1^ (*CDKN1A*), and reduction of heterochromatin-associated proteins (HP1α) (Supplementary Fig. S1b). Additionally, RS hMPCs have reduced cellular proliferation potential, as indicated by decreased Ki67 expression (Supplementary Fig. S1c) and clonal expansion ability (Fig. 1b). We conducted a series of experiments to investigate whether these predicted phase separation candidates within the SAGA members form condensates in the nucleus in senescent hMPCs (Supplementary Fig. S1d–i). Accordingly, our findings demonstrated that among the candidates, only SGF29, TAF5L, and ATXN7L2 exhibited a punctate pattern and increased nuclear localization in SA-β-Gal (SPiDER-βGal)-positive senescent cells (LP hMPCs) (Supplementary Fig. S1d–f). Notably, SGF29 exhibited the most prominent formation of condensates within the nucleus compared to TAF5L and ATXN7L2 (Supplementary Fig. S1d–f). In line with these results, the numbers of SGF29 puncta in the nuclei were positively correlated with expression levels of the senescence protein p21^Cip1^ (Fig. 1c–e) and negatively correlated with the protein level of HP1α, for which downregulation is a hallmark of aging (Fig. 1f, g). Consistently, analysis of the SGF29 distribution in subcellular compartments revealed that the nuclear and chromatin-bound fractions of SGF29 both increase largely in RS hMPCs relative to control (Fig. 1h).

In addition to the replicative senescence model, we also investigated SGF29 condensates in two genetically-driven models of premature aging: Hutchinson-Gilford Progeria Syndrome hMPCs (HGPS-hMPC, *LMNA*^G608G/+^ and *LMNA*^G608G/G608G^) and Werner Syndrome hMPCs (WS-hMPC, *WRN*^–/–^)^49–54^, in which hMPCs exhibit genetically accelerated senescence (Supplementary Fig. S2a–d). Indeed, in both senescent HGPS- and WS-hMPCs, we observed formation of nuclear SGF29 condensates, which is characterized by lower levels of HP1α expression as detected by immunofluorescence (Supplementary Fig. S2e). We also validated the specificity of the SGF29 antibody using CRISPR/Cas9-mediated gene knock-down approach (Supplementary Fig. S2f, g) and cDNA overexpression assays (Supplementary Fig. S2h, i). These data indicate that formation of SGF29 nuclear condensates is a novel feature of hMPC senescence.

To further clarify the functional role of SGF29 in cellular senescence, we constructed a lentiviral expression vector encoding EGFP-fused SGF29 and expressed this construct in young wild-type (WT) hMPCs. Unlike the expression pattern of EGFP in hMPCs (diffuse whole-cell distribution), overexpressed EGFP-SGF29 mainly exists in the form of nuclear condensates (Fig. 1i). Notably, an increased level of SGF29 nuclear condensates was associated with accelerated cellular senescence, as detected by an increased fraction of SA-β-Gal-positive cells, diminished cell proliferation rates, elevated p21^Cip1^ protein levels, as well as a lower expression of the proliferation marker Ki67 (Fig. 1j–l). In addition, a higher frequency of γH2AX and 53BP1 foci indicated an increase DNA damage response in SGF29-overexpressed cells (Fig. 1m). In contrast, the silence of SGF29 using siRNA, either in RS hMPCs or HGPS hMPCs, resulted in the attenuation of the cellular senescence, as evidenced by decreased expression of p21^Cip1^ (*CDKN1A*) and reduced signals of the SA-β-Gal signals (Fig. 1n, o; Supplementary Fig. S2j, k).

Next, we examined whether the senescence-associated formation of SGF29 condensates also occurred in other cell types. We employed human primary fibroblasts, a diploid cell type widely used in human replicative cell senescence research^55^, to determine the subcellular localization and pattern of SGF29. As expected, a number of SGF29 condensates with intense fluorescence signals were present in the nuclei of SPiDER-βGal positive aged human fibroblasts, compared to their phenotypically younger counterparts (Fig. 1p, q; Supplementary Fig. S3a–e). In particular, the acceleration of cellular senescence resulting from SGF29 overexpression (Supplementary Fig. S3f, g) and, conversely, the mitigation of cellular senescence by SGF29 knockdown, were also reproduced in RS human fibroblasts (hFib) (Supplementary Fig. S3h, i). These results suggest that formation of SGF29 condensates in the nucleus is a common feature across different cell types and potentially a driver of cellular senescence.

### SGF29 condensates exhibit liquid-like properties

**Fig. 2:**
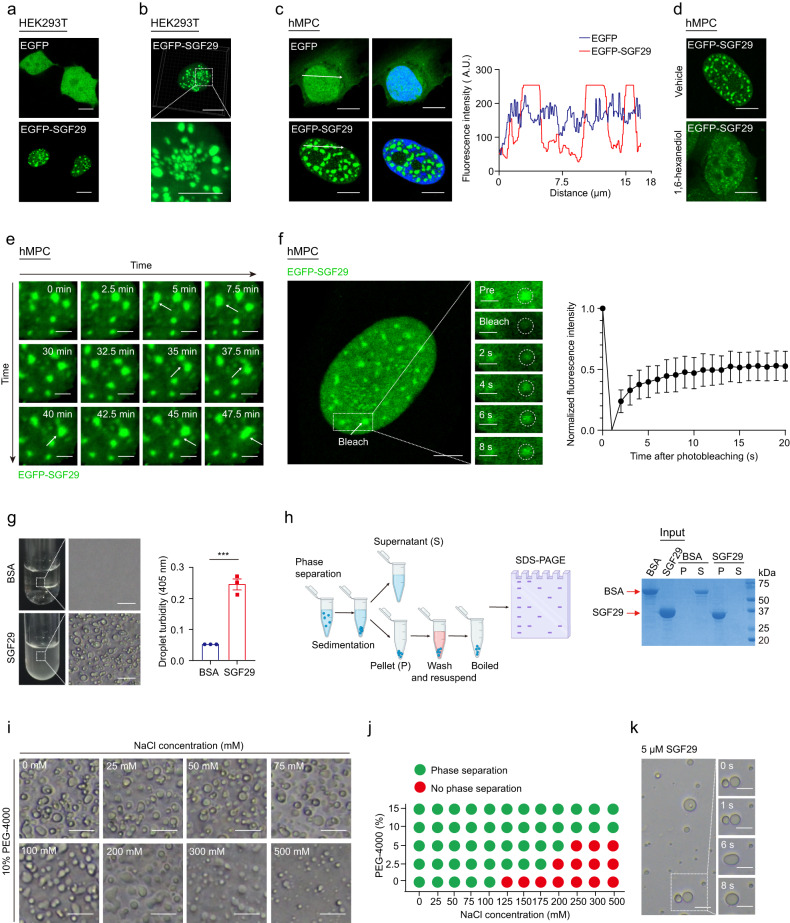
SGF29 spontaneously and reversibly assembles into liquid droplets. **a** Representative images of the HEK293T cells overexpressing either EGFP or EGFP-SGF29. Scale bars, 10 μm. **b** Representative 3D-reconstructed confocal images of the HEK293T cells overexpressing EGFP-SGF29. Scale bars, 10 μm and 5 μm (zoomed-in image). **c** Representative images of hMPCs overexpressing either EGFP or EGFP-SGF29. Quantification of the fluorescence intensity along the line embedded in images following the arrow direction is on the right. Scale bars, 10 μm. **d** Representative images of hMPCs before and after treatment with 10% v/v 1, 6-hexanediol (1, 6-HD) for 1 min. Scale bars, 10 μm. **e** Time-lapse images taken with a confocal microscopy, along with magnified illustrations, showing two adjacent EGFP-SGF29 aggregates fusing in hMPCs at the time point of 47.5-min. Scale bars, 2.5 μm. **f** Live-cell images of fluorescence recovery after photobleaching (FRAP) experiments in hMPCs expressing EGFP-SGF29. Left, representative time-lapse FRAP images of EGFP-SGF29 in hMPCs. Right, quantification of fluorescence intensity during FRAP assay. *n* = 7 hMPCs. Scale bars, 5 μm and 2.5 μm (zoomed-in image). **g** Differential interference contrast (DIC) microscopy analysis of test tube containing 5 μM BAS or 5 μM SGF29 at 25 °C. Left, representative DIC images. Scale bars, 50 μm. Right, quantification of the indicated droplet turbidity. Data are presented as the mean ± SEM. *n* = 3 biological replicates. ***P < 0.001 (*t*-test). **h** Sedimentation assay for SGF29. Left, diagram of the sedimentation assay which separates the condensed liquid phase and the aqueous phase for SDS-PAGE and Coomassie blue staining assays. Right, the BSA and SGF29 levels of the input, the solution (Supernatant, S) and separated droplets (Pellet, P) were assessed. **i** Formation of SGF29 droplets in 10% PEG-4000 solution containing different NaCl concentrations at 25 °C. Scale bars, 50 μm. **j** Diagram showing the formation of SGF29 droplets in buffers containing different NaCl concentrations and PEG-4000 concentrations. **k** The time-lapse images displaying fusion of SGF29 droplets in vitro. Scale bars, 50 μm.

The redistribution of SGF29 in the nuclei of senescent hMPCs (Fig. 1h), led us to hypothesize that SGF29 condensates may form through a process of concentration-dependent LLPS^56^. To test this hypothesis, we transduced either EGFP-SGF29 or EGFP alone by lentiviral vector in HEK293T cells and hMPCs. In both cell types, EGFP-SGF29 assembled into droplet-like spheres in the nuclei, while EGFP alone displayed the expected ubiquitous and diffuse fluorescence signal (Fig. 2a–c; Supplementary Fig. S4a, b). When we added 1, 6-hexanediol (Fig. 2d), a chemical widely used to dissolve phase-separated condensates^33^, we observed that the SGF29 droplets became dispersed. When we then performed live cell imaging to examine the dynamic liquid-like properties of the SGF29 condensates, we observed that some EGFP-SGF29 droplets underwent spontaneous fusion (Fig. 2e; Supplementary Video S1). Fluorescence recovery after photobleaching (FRAP) experiments revealed rapid exchange kinetics of SGF29 droplets (Fig. 2f; Supplementary Fig. S4c and Videos S2, S3), as previously described for other LLPS nuclear proteins^42^. These results demonstrated that SGF29 forms liquid-like droplets in the nuclei of live cells.

Next, we performed in vitro LLPS assays to identify characteristics of the SGF29 liquid-like droplets. To this end, we expressed and purified His-tagged full-length SGF29 protein from bacteria (Supplementary Fig. S4d). Interestingly, in the absence of crowding agents commonly used to induce formation of artificial liquid droplets, such as PEG-4000, induction of SGF29 droplet formation occurred in the buffer. Compared to the control solution containing bovine serum albumin (BSA), the solution containing full-length SGF29 appeared turbid (measured by OD 405 nm) (Fig. 2g). Examination under differential interference contrast microscopy (DIC) confirmed that the turbidity in the SGF29 solution was due to the presence of numerous spherical droplets (Fig. 2g). When we harvested the phase-separated droplets by sedimentation and analyzed it by SDS-PAGE, we could verify that SGF29, but not BSA, entered the droplet fraction (Fig. 2h). Since usually LLPS protein is highly responsive to changes in environmental factors such as salt and molecular crowding, we next varied the sodium chloride (NaCl) and PEG-4000 concentrations. We found that droplets with 5 μM SGF29 protein were stable in the presence of lower salt concentrations at room temperature (RT), but gradually disappeared at NaCl concentrations above 300 mM (Fig. 2i). The inclusion of PEG-4000 enhanced formation of LLPS, compared to the same salt concentration in the absence of PEG-4000 (Fig. 2j). Remarkably, we observed that the addition of total RNA to the SGF29 phase system increased the turbidity of the solution (Supplementary Fig. S4e), suggesting that the presence of RNA components, which is highly negatively charged, facilitates crowding of SGF29. Furthermore, we found that the droplets of purified SGF29 fused within a few seconds upon contact (Fig. 2k; Supplementary Video S4), validating their liquid nature. These in vitro experiments support that SGF29 droplets exhibit liquid-like properties.

### SGF29 phase separation is dependent on its C-terminal intrinsically disordered region

**Fig. 3:**
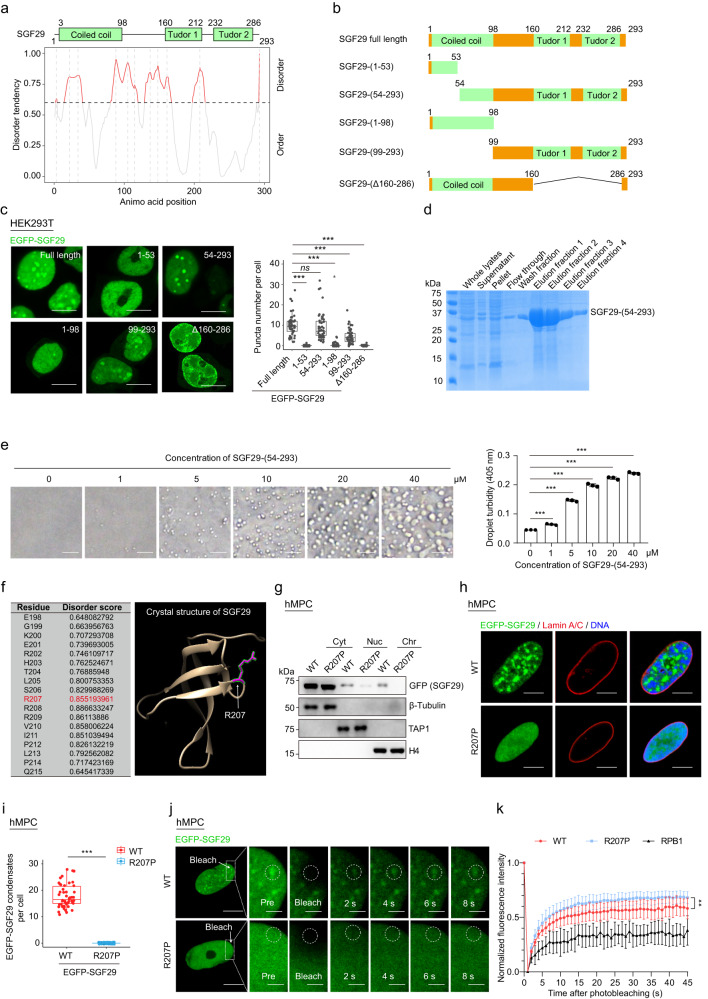
Tudor domains and Arginine 207 are necessary for SGF29 phase separation. **a** Protein sequence and disorder prediction (PONDR) of the SGF29. The number on the top represents the position of amino acids (aa). **b** Schematic diagram for EGFP-SGF29 truncation mutants. **c** Immunofluorescence images of EGFP-SGF29 truncation mutants in HEK293T cells. Left, representative immunofluorescence images. Right, quantification for the condensate numbers of EGFP-SGF29 truncation mutants in HEK293T cells. Data are shown as means ± SEM. *n* = 50 HEK293T cells. Scale bars, 10 μm. ns, not significant; ***P < 0.001 (*t-*test). **d** Coomassie blue staining of purified recombinant SGF29-(54-293) after being resolved on SDS-PAGE. **e** Formation of phase-separated condensates of purified recombinant SGF29 (54–293aa) forms phase-separated condensates at the indicated concentrations. Left, representative phase images for SGF29-(54–293) droplets. Scale bars, 50 μm. Right, quantification for the turbidity of SGF29-(54–293) droplet. Data are presented as the mean ± SEM. *n* = 3 biological replicates; ***P < 0.001 (*t-*test). **f** The PONDR and the crystal structure of SGF29. The white arrowheads indicate the position of Arg 207 residue in the crystal structure of SGF29. **g** Subcellular fractionation of exogenous EGFP-SGF29 in the cytoplasmic (Cyt), nuclear soluble (Nuc) and chromatin-associated (Chr) fractions in EP WT hMPCs (P5, young hMPCs) transduced with lentiviruses expressing EGFP-SGF29-WT (WT) or EGFP-SGF29-R207P (R207P). β-Tubulin, TAP1, and H4 were used as the loading control, respectively. **h** Representative images of hMPCs transduced with lentiviruses expressing either EGFP-SGF29-WT (WT) or EGFP-SGF29-R207P (R207P). Scale bars, 10 μm. **i** Quantification of SGF29 puncta number per cell in hMPCs transduced with lentiviruses expressing either EGFP-SGF29-WT (WT) or EGFP-SGF29-R207P (R207P). *n* = 50 hMPCs. Data are presented as the mean ± SEM. ***P < 0.001 (*t-*test). **j** Representative time-lapse FRAP images acquired in hMPCs transduced with lentiviruses expressing either EGFP-SGF29-WT (WT) or SGF29-R207P (R207P) with magnified insets showing the pre-bleach and recovery signals of EGFP-SGF29-WT (WT), EGFP-SGF29-R207P (R207P), respectively. Scale bars, 10 μm and 2.5 μm (zoomed-in image). **k** The curve showing the quantification of fluorescence intensity in FRAP recovery assay indicated in Fig. 3j and Supplementary Fig. S5c. EGFP-RPB1 was used as the slow recovery control. *n* = 7 hMPCs. **P < 0.01 (*t-*test).

Intrinsically disordered regions (IDRs) are highly enriched in proteins that are capable of undergoing phase separation and have been demonstrated to be important for this process^57–59^. Overall, human SGF29 contains a coiled-coil domain (3–98) at the N-terminus and two tandem Tudor domains (160–212 and 232–286) at the C-terminus, both of which are predicted to be flexible and unstructured^8,47,60–63^. However, when utilizing the Predictor of Natural Disordered Regions algorithm, we found that the full-length SGF29 protein contains four IDRs, with the first region of IDR located in amino acids 1–53 at the N-terminus region, and the remaining IDRs convergent in 54–293 at the C-terminus end (Fig. 3a; Supplementary Table S1).

To test the importance of SGF29 IDRs in phase transition, we produced five truncated SGF29 proteins: (1) 1–53, the N-terminal IDR region; (2) 54–293, SGF29 lacking the N-terminal IDR but including the remaining three IDRs; (3) 1–98, the coiled-coil domain; (4) 99–293, SGF29 lacking the coiled-coil domain; and (5) Δ160–286, SGF29 lacking the two Tudor domains (Fig. 3b). We then assessed droplet formation efficiency of the truncated SGF29 proteins by fluorescence microscopy. Similar to full-length SGF29, we found that SGF29 (54–293) formed a number of liquid-like puncta in the nuclei. In contrast, SGF29 (1–53), SGF29 (1–98), and SGF29 (Δ160–286) all lost the ability to concentrate into droplets (Fig. 3c), validating that the tandem Tudor domains are functionally required for SGF29 phase separation. We then produced recombinant C-terminal truncation variant of SGF29 (SGF29-(54-293)) (Fig. 3d) and found that it retained the capability to undergo phase separation similar to the full-length protein (Fig. 3e). Furthermore, by analyzing the crystal structure of human SGF29 (PDB ID: 3MEU) with the ChimeraX program, we recognized 12 residues located at the region connecting two β folds, of which the mutations were predicated to lower the disorder score of SGF29 (Fig. 3f). Among the 18 residues, it is noteworthy that arginine at position 207 (R207) exhibited a high disorder score and occupied a crucial corner location (Fig. 3f). Intriguingly, subcellular fractionation analysis revealed that SGF29-R207P exhibited a reduced presence in the nuclear fraction and was barely detectable in the chromatin bound fraction, despite having a comparable expression level to SGF29-WT in the total cellular lysate (Fig. 3g; Supplementary Fig. S5a, b). In contrast, SGF29-WT exists substantially in both nuclear and chromatin bound fractions (Fig. 3g). Moreover, we discovered that the SGF29-R207P mutant failed to concentrate into droplets in hMPCs (Fig. 3h, i) and the recovery of the SGF29-R207P was substantially faster than its SGF29 WT counterparts and the slow recovery control RPB1 (the largest subunit of RNA polymerase II (RNApII))^42^ by FRAP assay (Fig. 3j, k; Supplementary Fig. S5c), indicating an essential role of R207 in mediating SGF29 phase separation and chromatin binding.

A previous study demonstrated that certain residues in the Tudor domains of SGF29 are responsible for its binding to histone H3K4me3^43^. To determine whether the interaction between H3K4me3 and SGF29 contributes to phase separation, we ectopically expressed four EGFP-tagged interaction-defective mutants SGF29-D194A, SGF29-D196A, SGF29-Y245A, SGF29-F264A^43^ in hMPCs. We found that all four mutants successfully assembled into droplets in nuclei, to an extent comparable to that of WT SGF29 (Supplementary Fig. S5a, d). These data suggest that phase separation as a biological process does not depend on the activity of SGF29 to recognize H3K4me3. Instead, our data demonstrate that the residues situated in the IDRs within the tandem Tudor domains are essential for SGF29 condensation.

### Proper recognition and binding to H3K4me3 by SGF29 requires its phase separation

**Fig. 4:**
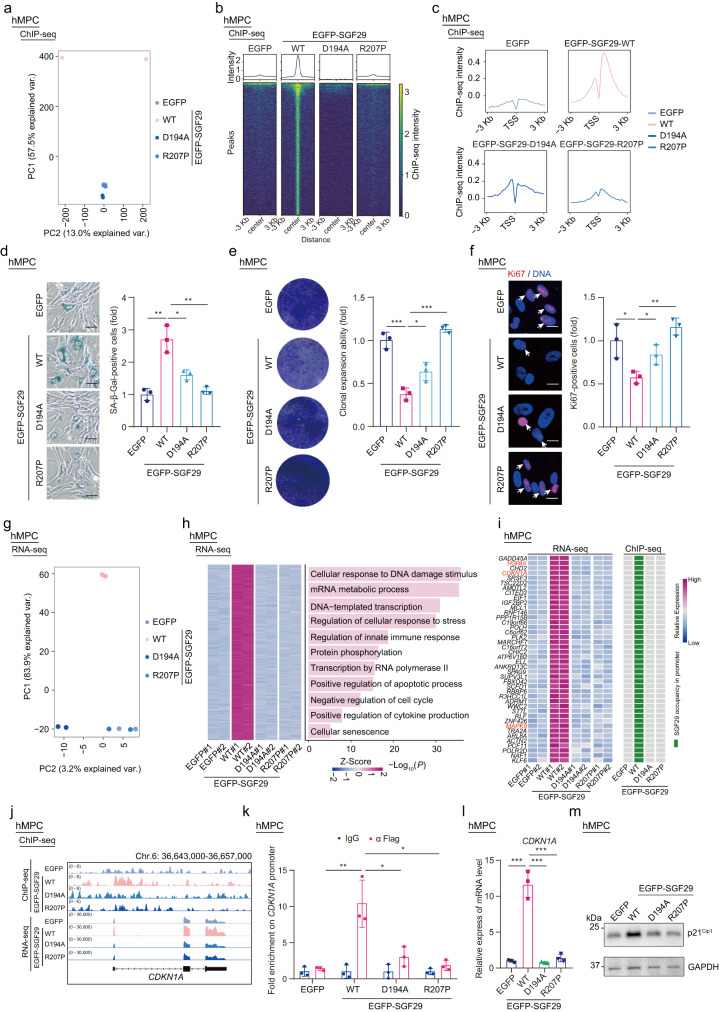
SGF29 phase separation directs a transcriptional program favorable to senescence. **a** Principal Component Analysis (PCA) of SGF29 ChIP-seq data in hMPCs (P13) with CRISPR/Cas9-mediated knockdown of endogenous SGF29 followed by overexpression of EGFP, EGFP-SGF29-WT (WT), EGFP-SGF29-D194A (D194A) and EGFP-SGF29-R207P (R207P) variants, respectively. **b** Heatmap showing the chromatin occupancy profiles of SGF29 in hMPCs (P13) with CRISPR/Cas9-mediated knockdown of endogenous SGF29 followed by expression of EGFP, EGFP-SGF29-WT (WT), EGFP-SGF29-D194A (D194A) and EGFP-SGF29-R207P (R207P) variants, respectively. Peaks identified in hMPCs with CRISPR/Cas9-mediated knockdown of endogenous SGF29 followed by overexpression of EGFP-SGF29-WT (*n* = 2890) were used for comparison in all groups. **c** Metaplots showing the enriched levels of SGF29 occupancies surrounding the TSS regions for protein-coding genes in hMPCs (P13) with CRISPR/Cas9-mediated knockdown of endogenous SGF29 followed by overexpression of EGFP, EGFP-SGF29-WT (WT), EGFP-SGF29-D194A (D194A) and EGFP-SGF29-R207P (R207P) variants, respectively. **d** SA-β-Gal staining of hMPCs (P13) with CRISPR/Cas9-mediated knockdown of endogenous SGF29 followed by expression of EGFP, EGFP-SGF29-WT (WT), EGFP-SGF29-D194A (D194A) and EGFP-SGF29-R207P (R207P) variants, respectively. Left, representative images of SA-β-Gal staining. Scale bars, 50 μm. Right, quantitation of the relative percentages of SA-β-Gal-positive cells. Data are presented as the mean ± SEM. *n* = 3 biological replicates. Over 100 cells were quantified in each replicate. *P < 0.05; **P < 0.01 (*t-*test). **e** Clonal expansion assay in hMPCs (P13) with CRISPR/Cas9-mediated knockdown of endogenous SGF29 followed by overexpression of EGFP, EGFP-SGF29-WT (WT), EGFP-SGF29-D194A (D194A) and EGFP-SGF29-R207P (R207P) variants, respectively. Left, representative images of crystal violet staining. Right, quantification of the relative clonal expansion ability. Data are presented as the mean ± SEM. *n* = 3 biological replicates. *P < 0.05, ***P < 0.001 (*t-*test). **f** Immunofluorescence staining of Ki67 in hMPCs (P13) with CRISPR/Cas9-mediated knockdown of endogenous SGF29 followed by overexpression of EGFP, EGFP-SGF29-WT (WT), EGFP-SGF29-D194A (D194A) and EGFP-SGF29-R207P (R207P) variants, respectively. Left, representative images of Ki67 immunofluorescence staining. Scale bars, 20 μm. Right, quantification of the relative percentages of Ki67-positive cells. Data are presented as the mean ± SEM. *n* = 3 biological replicates. Over 100 cells were quantified in each replicate. *P < 0.05; **P < 0.01 (*t-*test). **g** PCA of transcriptomic profiles in hMPCs (P13) with CRISPR/Cas9-mediated knockdown of endogenous SGF29 followed by overexpression of EGFP, EGFP-SGF29-WT (WT), EGFP-SGF29-D194A (D194A) and EGFP-SGF29-R207P (R207P) variants, respectively. **h** Heatmap showing the relative expression of indicated genes, which were activated in hMPCs expressing EGFP-SGF29-WT (WT) but remained silent in hMPCs expressing EGFP-SGF29-D194A (D194A) and EGFP-SGF29-R207P (R207P) mutants, in all groups. Representative Gene Ontology (GO) terms are shown on the right. **i** Heatmaps showing the relative transcriptional levels and SGF29 occupancies surrounding the TSS regions of 42 genes, which were activated in hMPCs expressing SGF29-WT but remained silent in hMPCs expressing EGFP, or D194A and R207P mutants. **j** Integrative Genome Viewer tracks of the ChIP-seq and RNA-seq signals at *CDKN1A* locus in hMPCs (P13) with CRISPR/Cas9-mediated knockdown of endogenous SGF29 followed by overexpression of EGFP, EGFP-SGF29-WT (WT), EGFP-SGF29-D194A (D194A) and EGFP-SGF29-R207P (R207P) variants, respectively. **k** Bar plot showing the ChIP-qPCR detection of the SGF29 enrichment at *CDKN1A* promoter in hMPCs (P13) with CRISPR/Cas9-mediated knockdown of endogenous SGF29 followed by overexpression of EGFP, EGFP-SGF29-WT (WT), EGFP-SGF29-D194A (D194A) and EGFP-SGF29-R207P (R207P) variants, respectively. Data are presented as the mean ± SEM. *n* = 3 biological replicates. *P < 0.05; **P < 0.01 (*t-*test). **l** Bar plot showing the qPCR detection of the mRNA levels of *CDKN1A* in hMPCs (P13) with CRISPR/Cas9-mediated knockdown of endogenous SGF29 followed by expression of EGFP, EGFP-SGF29-WT (WT), EGFP-SGF29-D194A (D194A) and EGFP-SGF29-R207P (R207P) variants, respectively. Data are presented as the mean ± SEM. *n* = 3 biological replicates. ***P < 0.001 (*t-*test). **m** Western blotting detected the protein expression of p21^Cip1^ in hMPCs (P13) with CRISPR/Cas9-mediated knockdown of endogenous SGF29 followed by expression of EGFP, EGFP-SGF29-WT (WT), EGFP-SGF29-D194A (D194A) and EGFP-SGF29-R207P (R207P) variants, respectively.

Notably, even though SGF29-R207P mutants show deficiencies in both condensate formation (Fig. 3h–k) and chromatin binding (Fig. 3g), other mutants with impaired H3K4me3 recognition and chromatin binding, such as D194A, D196A, Y245A, and F264A, still exhibit condensate formation (Supplementary Fig. S5a, d). This raises intriguing questions regarding the contribution of SGF29 nuclear condensate formation to its recognition and binding to H3K4me3 promoter sites. Therefore, we expressed the EGFP-SGF29-WT, H3K4me3 recognition-deficient mutant (EGFP-SGF29-D194A) and LLPS-incompetent mutant (EGFP-SGF29-R207P) in young hMPCs with the endogenous SGF29 knockdown by CRISPR/Cas9 approach and carried out chromatin immunoprecipitation assays followed by sequencing (ChIP-seq) for SGF29 in hMPCs ectopically expressing EGFP-SGF29-WT, EGFP-SGF29-R207P or EGFP-SGF29-D194A. We observed distinct patterns of chromatin occupancy for SGF29 variants compared to SGF29-WT (Fig. 4a, b; Supplementary Fig. S6a, b). Specifically, both SGF29-D194A and SGF29-R207P mutants exhibited extensive decreases in enrichment around transcriptional start sites (TSSs) compared to WT (Fig. 4c; Supplementary Fig. S6c). This finding is consistent with previous reports on the reduced binding affinity of SGF29-D194A to H3K4me3^43^. Moreover, considering that SGF29-R207P is barely detectable in the chromatin-bound fraction of hMPCs (Fig. 3g), our results demonstrate that condensate formation of SGF29 is crucial for its appropriate promoter binding.

### SGF29 LLPS facilitates the activation of genes related to cellular senescence

Considering the fact that LLPS-incompetent variant of SGF29 exhibited compromised promoter recognition and binding ability, we investigated the impact of SGF29 phase separation on expression of genes associated with cellular senescence. To this end, we expressed the SGF29-WT, SGF29-D194A and SGF29-R207P in young hMPCs with the endogenous SGF29 knockdown, and found that SGF29-WT expression led to the acquisition of various senescence phenotypes, including increased SA-β-Gal positive cells and compromised proliferative activities (Fig. 4d–f). Whereas, expression of the SGF29-D194A or SGF29-R207P reduced the ability to induce cellular senescence (Fig. 4d–f). These data support the importance of proper H3K4me3 recognition and binding for SGF29 in driving cellular senescence.

Next, we performed genome-wide RNA sequencing (RNA-seq) to gain insights into the transcriptional signatures associated with cellular senescence. Euclidean distance measurement (Supplementary Fig. S6d) and principal component analysis (Fig. 4g) revealed the transcriptomic profile in hMPCs expressing SGF29-WT was distinct from those of hMPCs transduced with SGF29 mutants or EGFP control, which were similar (Supplementary Fig. S6e, f). Gene ontology (GO) enrichment analysis revealed that differentially expressed genes (DEGs) associated with SGF29-WT were enriched in aging-related biological processes, such as cellular senescence, DNA damage, stress response and immune response. However, these gene expression changes were not observed in hMPCs expressing SGF29-D194A or SGF29-R027P (Fig. 4h; Supplementary Fig. S6e, f). These results further support the notion that SGF29 promotes the activation of genes related to senescence.

Integrative analysis of SGF29 ChIP-seq and RNA-seq data revealed that SGF29 occupancies were enriched at TSSs and positively correlated with the magnitude of gene expression (Fig. 4c; Supplementary Fig. S6g). Genes that were activated exclusively in cells expressing SGF29-WT but remained silence in cells expressing SGF29-D194A and SGF29-R207P mutants displayed higher SGF29 occupancy levels around their TSSs, indicating that their activation may be driven by direct binding of SGF29 at their TSSs (Supplementary Fig. S6h). A total 42 genes were identified to exhibit unique SGF29 occupancies and gene activation in cells expressing SGF29-WT. Notably, among these genes, *HSPA9, CDKN1A*, and *MAPK8* have previously been annotated as aging hotspot genes in the Aging Atlas database^64^ (Fig. 4i; Supplementary Fig. S6i). Of particular interest was *CDKN1A* (encoding p21^Cip1^), a well-known senescence biomarker protein that regulates cyclin-dependent kinase (CDKs) by preventing their phosphorylation by retinoblastoma gene (*RB*), thus controlling G1/S cell cycle progression^65–68^ (Fig. 4j). Through ChIP followed by quantitative PCR analysis, we confirmed that SGF29-WT specifically occupied the *CDKN1A* gene promoter (Fig. 4k), while both SGF29-R207P and SGF29-D194A exhibited decreased binding to this locus (Fig. 4k). Consistent with the notion that SGF29 activates *CDKN1A* transcription, both mRNA and protein levels of p21^Cip1^ were upregulated in hMPCs expressing SGF29-WT. However, such phenotypes were not observed in hMPCs expressing SGF29-R207P or SGF29-D194A mutants (Fig. 4l, m). Altogether, these results underscore the critical role of SGF29 phase separation in facilitating appropriate promoter binding, which is essential for activating senescence genes like *CDKN1A*.

### Identification of SGF29 interacting proteins sensitive to condensate perturbation

**Fig. 5:**
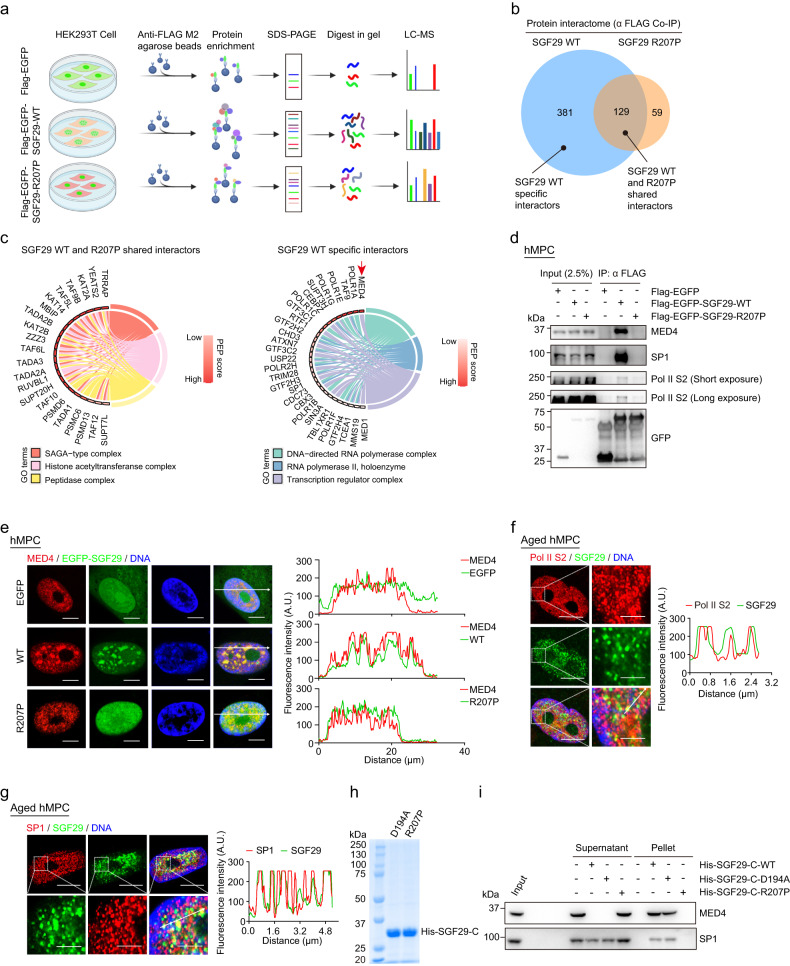
Identification of SGF29 interacting proteins sensitive to condensate perturbation. **a** Flow chart of the mass spectrometry strategy for the identification of EGFP-SGF29-WT (WT) and EGFP-SGF29-R207P (R207P) interacting proteins. Flag-EGFP was used as a control. **b** Shared and specific interacting proteins of EGFP-SGF29-WT (WT) and EGFP-SGF29-R207P (R207P) identified by mass spectrometry. **c** Chord diagrams showing the enriched pathways of shared interaction partners of EGFP-SGF29-WT (WT) and EGFP-SGF29-R207P (R207P) (left) and those of specific protein interaction partners of EGFP-SGF29-WT (right). **d** Co-IP analysis showing the interaction between indicated proteins and EGFP-SGF29-WT (WT) and EGFP-SGF29-R207P (R207P) in hMPCs. **e** Immunofluorescence staining of MED4 in hMPCs transduced with lentiviruses expressing either EGFP, EGFP-SGF29-WT (WT) or EGFP-SGF29-R207P (R207P). Left, representative images. Scale bar, 10 μm. Right, quantification of the fluorescence intensity along the line embedded the image following the arrow direction. **f** Immunofluorescence staining of Pol II S2 and SGF29 in senescent hMPCs. Left, representative images. Scale bars, 10 μm and 5 μm (zoomed-in image). Right, quantification of the fluorescence intensity along the line embedded the image following the arrow direction. **g** Immunofluorescence staining of SP1 and SGF29 in senescent hMPCs. Left, representative images. Scale bars, 10 μm and 5 μm (zoomed-in image). Right, quantification of the fluorescence intensity along the line embedded the image following the arrow direction. **h** Coomassie blue staining of purified recombinant SGF29-C-D194A and SGF29-C-R207P after being resolved on SDS-PAGE. **i** Pelleting assay show that SGF29-C-D194A and SGF29-C-R207P interact with MED4 and SP1.

We next sought to investigate whether SGF29 partner with other protein components to form condensates and contribute to transcriptional activation. Accordingly, we conducted co-immunoprecipitation followed by mass spectrometry (co-IP/MS) in HEK293T cells engineered to express either SGF29-WT or SGF29-R207P protein with flag and EGFP dual tags (Fig. 5a). This approach allowed us to identify 510 proteins that were associated with SGF29-WT and only 188 proteins interacting with the LLPS-deficient mutant SGF29-R207P (Fig. 5b; Supplementary Table S2), demonstrating that interactions between SGF29 and the majority of its binding partners may be LLPS-dependent (Fig. 5b; Supplementary Table S2). Both WT and LLPS-deficient mutant SGF29-R207P can bind to the subunits of the SAGA and ATAC complexes, including KAT2A (GCN5) and KAT2B, ADA2A, and ADA3 (Fig. 5c; Supplementary Fig. S7a–c)^43,44^, which were the most enriched protein categories based on GO enrichment analysis (Fig. 5c; Supplementary Table S2). In addition, we validated that both SGF29-WT and SGF29-R207P protein interacted with ADA3, and with GCN5, the HAT subunit of the SAGA and ATAC complex, suggesting that the disruption of SGF29 condensates does not affect its interaction with the HAT module (Supplementary Fig. S7d–f). Moreover, knockdown of GCN5, resulted in a similar decrease in *CDKN1A* expression and a delay in cellular senescence in human fibroblasts (Supplementary Fig. S7g–j), suggesting the involvement of the HAT module in SGF29-mediated cellular senescence.

Binding partners that lost interaction with SGF29 when its phase separation ability was abrogated included proteins implicated in nuclear DNA−directed RNA polymerase complex, RNA polymerase II, holoenzyme, and transcription regulator complex (Fig. 5d). In the list of candidate proteins, we particularly focused on the mediator of RNA polymerase II transcription subunit 4 (MED4), a key component involved in gene-specific transcription initiation^16^, and the protein with the highest affinity score (Fig. 5d; Supplementary Figs. S7a, S8a). Transcription factor SP1 and more than 10 other components of the RNA Pol II transcription complex were also captured as the above all three pathways (e.g., MED4, SP1, GTF2H2, GTF2H3, GTF2H4, POLR2H, TAF9, TCEA, SUPT3H, RTF1, MMS19, and CDC73) (Fig. 5d; Supplementary Figs. S7a, S8a). Notably, based on transcription factor prediction analysis performed on the genes annotated as aging related genes in aging atlas and those exclusively upregulated in hMPCs expressing SGF29-WT, we identified SP1 as the predominant transcription factor that promoted the expression of senescence-associated genes in hMPCs expressing SGF29-WT (Supplementary Fig. S8b). The analyses collectively suggest that SGF29 condensates may associate with MED4, SP1 and the RNA Pol II transcriptional complex, as exemplified by the Ser2 phosphorylated C-terminal domain (CTD) of RPB1 (Pol II S2). Additional co-IP experiments validated that SGF29 forms a protein complex with Pol II S2, SP1 and MED4 under LLPS condition but not under non-LLPS condition driven by the R207P mutant (Fig. 5d). A reverse co-IP experiment using MED4 antibody for immunoprecipitation further confirmed that MED4 bound to endogenous SGF29 and SP1 (Supplementary Fig. S8c). In addition, when we performed quantitative line-scan immunofluorescent analyses, we found that MED4 colocalized with SGF29-WT in nuclear condensates, whereas the SGF29-R207P mutant was uniformly distributed throughout the nucleus (Fig. 5e). Quantitative line-scan immunofluorescent analyses showed that SGF29 colocalized with the Pol II or SP1 in nuclear condensates (Fig. 5f, g; Supplementary Fig. S8d). To further interrogate whether MED4 and SP1 were preferentially partitioned within SGF29 condensates, we expressed and purified several variants of His-tagged SGF29 recombination proteins, including SGF29 C terminal truncation variants (SGF29-C-WT, SGF29-C-D194A, SGF29-C-R207P) (Fig. 5h). We then added these protein variants into soluble nuclear extract in vitro and performed pelleting assays (Supplementary Fig. S8 e, f). We observed that SGF29-C-WT and SGF29-C-D194A formed liquid-like droplets whereas SGF29-C-R207P did not. Furthermore, through western blot analysis, we detected the presence of MED4 and SP1 in the droplet pellets of both SGF29-C-WT and SGF29-C-D194A (Fig. 5i), validating the incorporation of MED4 and SP1 into SGF29 condensates. Together, the above results suggest that the R207 amino acid residue of SGF29 is required for its multivalent interactions with other transcription factors/co-factors such as MED4, SP1 and potentially others.

### SGF29 and coactivators form condensates at the *CDKN1A* promoter to accelerate cell senescence

**Fig. 6:**
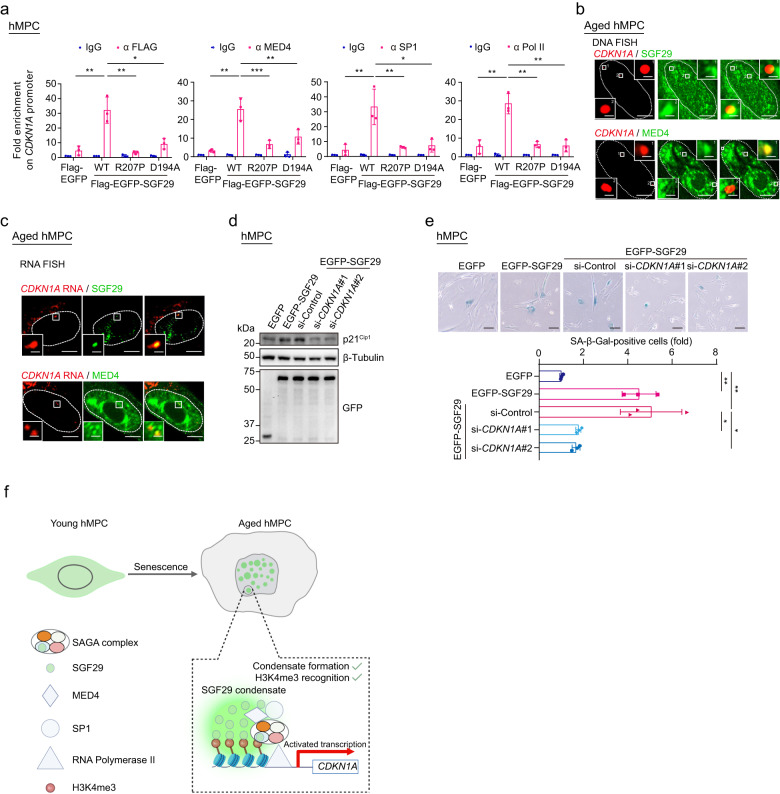
SGF29 and coactivators form condensates at the *CDKN1A* promoter to accelerate cell senescence. **a** Bar plot showing the enrichment of indicated proteins at the promoter of *CDKN1A* in hMPCs transduced with lentiviruses expressing either Flag-EGFP, Flag-EGFP-SGF29-WT (WT), Flag-EGFP-SGF29-R207P (R207P) or Flag-EGFP-SGF29-D194A (D194A). Data are presented as the mean ± SEM. *n* = 3 biological replicates. **P < 0.01, *P < 0.05 (*t*-test). **b** Immunofluorescence images of senescent hMPCs showing that SGF29 (green) colocalizes with *CDKN1A* gene (red) at the SGF29 or MED4 droplets. Scale bars, 10 μm and 1 μm (zoomed-in image). **c** Immunofluorescence images of senescent hMPC showing that MED4 (green) colocalizes with *CDKN1A* RNA (red) at the SGF29 or MED4 droples. Scale bars, 10 μm and 1 μm (zoomed-in image). **d** Western blot analysis for GFP and p21^Cip1^ in hMPCs transduced with lentiviruses expressing EGFP-SGF29 followed by knockdown of *CDKN1A* (p21^Cip1^) using siRNA. β-tubulin was used as the loading control. **e** SA-β-Gal staining of hMPCs transduced with lentiviruses expressing EGFP-SGF29 followed by knockdown of *CDKN1A* (p21^Cip1^) using siRNA. Top, representative images of SA-β-Gal staining. Scale bars, 50 μm. Bottom, quantification of the relative percentages of SA-β-Gal-positive cells. Data are presented as the mean ± SEM. *n* = 3 biological replicates. Over 100 cells were quantified in each replicate. **P < 0.01 (*t-*test). **f** The proposed model illustrates the pivotal role of SGF29 condensates in facilitating promoter-binding of SGF29 and recruitment of transcriptional factor and co-activators to target specific genomic loci, thereby initiating expression of senescence-related genes, such as *CDKN1A*.

Upon ChIP followed by quantitative PCR analysis, we confirmed that the partners of SGF29, including MED4, SP1 and RNA Pol II, were all enriched at *CDKN1A* promoter in the presence of SGF29-WT, but not in the presence of SGF29-R207P mutant, further suggesting that their potential collaboration as a complex in regulating *CDKN1A* gene expression (Fig. 6a). Next, we confirmed both SGF29 and MED4 condensate puncta were present at the *CDKN1A* chromatin foci using immunofluorescence staining analysis with fluorescence in situ hybridization (FISH) (Fig. 6b). The other way around, *CDKN1A* nascent RNA FISH assay revealed that the transcription foci of *CDKN1A* were located within both SGF29 and MED4 condensates (Fig. 6c). Furthermore, the knockdown of *CDKN1A* in hMPCs expressing SGF29-WT alleviated cellular senescence (Fig. 6d, e). These results indicate that senescence-associated formation of SGF29 intranuclear condensates functions as active transcription hubs for senescence-associated genes like *CDKN1A*, coordinating interactions with specific transcriptional factors and cofactors, thereby contributing to cellular senescence.

Collectively, our findings provide compelling support for the role of SGF29 phase separation in facilitating its partition with transcriptional partners and target genes. This process enables its recognition and activation of pro-senescent genes, including the cell cycle regulator *CDKN1A*, ultimately culminating in cellular senescence.

## Discussion

Our data reveal that SGF29 forms liquid-like condensates in the nuclei of senescent cells, but not in young cells. However, when we ectopically expressed SGF29 in young cells, we detected a similar accumulation of SGF29-containing nuclear condensates, and that the young cells acquired senescent cellular phenotypes. The SGF29 condensates formed in the cell nucleus appear liquid-like, and resemble those of phase-separated droplets assembled in vitro by recombinant SGF29 protein. We also found that the intrinsically disordered Tudor domains at the C-terminus of SGF29 are required for condensate formation. At a molecular level, we identify the arginine 207 of SGF29 as instrumental for formation of nuclear condensates, and that the R207P mutation, which alters the biophysical properties of SGF29, disrupts the ability of SGF29 to execute phase separation. At a mechanistic level, our findings reveal that condensate formation of SGF29 may not be sufficient on its own to drive its precise binding to specific promoters. Instead, both condensate formation and H3K4me3 binding of SGF29 are important for its proper chromatin location and roles in gene activation, thereby initiating gene expression related to senescence, including *CDKN1A* (Fig. 6f). These findings unravel a novel paradigm for understanding how SGF29, through phase separation, mediates dynamic changes in transcriptional activation and chromatin organization during aging.

Coactivator complexes play an important role in the formation of multivalent transcriptional machinery and activation of gene transcription by interacting with basal transcription factors, nucleosome remodeling, and histones modification^41^. Recent studies have brought to light the existence of transcriptional condensates that function as sites in which components of the transcriptional machinery, including transcription factors, cofactors, RNA polymerase II, and clusters of enhancers are concentrated and organized^8,20,69,70^. BRD4 and MED1 form liquid-like condensates at super enhancer sites, where they compartmentalize and concentrate transcriptional apparatus to maintain the expression of key cell-identity genes^33^. Similarly, Cho et al. found that large clusters of Mediator interact with clustered enhancer elements and Pol II in transcriptional condensates, which are prone to change as mESCs differentiate^16^. Further research revealed that MED1 condensates trigger transcription activation by selectively partitioning RNA polymerase II and its positive allosteric regulators while excluding negative regulators, which is necessary for adipocyte cell-state transition^71^. Another study focused on TAZ condensates and unveiled their function in transcriptional activation in response to regulation of Hippo signaling through compartmentalizing DNA-binding cofactor TEAD4, coactivators BRD4 and MED1, as well as the transcription elongation factor CDK9^72^. Together, these emerging reports strongly suggest that phase separation-dependent interactions are a common regulatory mechanism in transcriptional regulation.

SGF29, primarily known as a “reader” of histone methylation, is required for the recruitment of the SAGA complex and the subsequent acetylation of histone H3 at the target loci^43^. As a key histone modulator, SGF29 facilitates chromatin opening and transcriptional activation by interacting with transcription factors, mediator complexes and chromatin remodelers^73,74^. The current theory of coacervation posits that phase separation is driven by forces generated by weak electrostatic and hydrophobic interactions between amino acid side chains and interacting biomolecules. In this study, we demonstrated senescence-associated formation of protein condensates in the nuclei of senescent hMPCs and unraveled that the arginine at position 207 is necessary for the phase transition capability of SGF29, which makes it possible to determine whether biological activities of SGF29 are phase separation dependent or independent. Consistent with the discovery that positively charged proteins are favorable to co-phase with negatively charged biomolecules, such as nucleic acids, we found that the addition of RNA promotes formation of SGF29 droplets in vitro (Supplementary Fig. S4e). In addition, phase-separated SGF29 droplets are sensitive to salt concentration (Fig. 2i, j), consistent with electrostatic effects. In the cell nucleus, the transcriptional condensates act as transcriptional hubs where a large amount of nascent RNA molecules are synthesized^75^. Notably, senescent hMPCs show remarkably increased transcripts of repetitive elements such as ERV, LINE1, ribosomal DNA, and microsatellite sequences, which leads to an abundance of negative charge compared to young cells^1,76–81^. These observations may help us understand association between the formation of SGF29 condensates and cellular aging. Presumably, condensate formation may constitute a positive feedback loop that reinforces the cellular aging transcription program, which in turn generates novel transcripts further driving the process of condensate formation. When considering that the intranuclear microenvironment is associated with cellular aging and probably contains multiple factors that affect phase transition, its conceivable that targeting the assembly of SGF29 condensates might have therapeutic potential as a future aging intervention.

In this study, we specifically investigated the role of LLPS in partitioning of SGF29 and other cofactors, leading to the transcriptional activation of senescence-associated genes. We showed that nuclear condensate formation of SGF29 is necessary but not sufficient for its proper genomic binding, both condensate formation and H3K4me3 recognition are indispensable to SGF29 for appropriate chromatin binding, the concerted action of various transcription factors and cofactors, and ultimately leading to efficient and specific transcription activation of senescence-associated genes such as *CDKN1A*, thereby promoting cellular senescence (Fig. 6f). However, there may be additional mechanisms beyond the scope of this study that deserve attention in future studies. One intriguing aspect that emerged from our findings is the potential interaction of SGF29 with TCOF1, also known as treacle, which was identified as a specifically enriched protein of SGF29 immunoprecipitates (Supplementary Table S2, Fig. S8g). TCOF1 is known to play a critical role in ribosome biogenesis by binding and recruiting Pol I, UBF, and Nopp140 to the rDNA promoter^82–85^. Moreover, we also identified POLR1A, a subunit of RNA polymerase I, as a potential interacting protein of SGF29 (Fig. 5c). Notably, previous studies have demonstrated increased ribosomal DNA transcripts in senescent hMPCs^79^. These findings suggest that SGF29 may regulate cellular aging through its involvement in rDNA transcription, which warrants further investigation.

In summary, our study unraveled that SGF29 drives cellular senescence by controlling phase condensation and the compartmentalization of transcriptional partner proteins and target gene sites. The observed relationship between phase separation and aging suggests that phase separation can be understood as a general mechanism for interpreting dynamic changes and critical events accompanying aging. Additionally, our findings highlight an increased complexity of biological macromolecules in regulating the aging process in time and space. Finally, the previously unappreciated phase transition of SGF29, which is demonstrated to highly correlate with and substantially contribute to the progression of cellular senescence, may serve as a novel biomarker of aging. Actionable strategies to specifically target the assembly process of SGF29 condensates may represent an important direction to intervene in human aging program.

## Materials and methods

### Cell culture and RNA interference

The hMPCs were cultured on 0.1% gelatin (Sigma, G1393)-coated plates (CORNING), using hMPC culture medium comprising 90% MEMα (Gibco, 32571-101), 10% FBS (Gibco, 42F1190K), 2 mM GlutaMAX (Gibco, 35050079), 0.1 mM NEAA (Gibco, 11140076), 1% penicillin/streptomycin (Gibco, 15140-163) and 1 ng/mL bFGF (Joint Protein Central, 100120). Cells were cultured in an incubator (Thermo Fisher Scientific) at 37 °C with 5% CO_2_. Transient siRNA transfection was performed using 50 nM, non-targeting siRNA (si-Control), or siRNAs targeting human *SGF29* (si-*SGF29*), *CDKN1A* (si-*CDKN1A*), and *GCN5* (si-*GCN5*), which were purchased from Beijing Tsingke Biotech Co. Ltd (Beijing, China).

### Extraction of cytoplasmic, nuclear and chromatin bound proteins

The extraction of cytoplasmic, nuclear and chromatin bound proteins in RS hMPCs or expressing EGFP-SGF29-WT or EGFP-SGF29-R207P hMPCs was performed as previously described^78,86^. In brief, 1 × 10^7^ living cells were washed with cold PBS followed by centrifugation, and the supernatant was discarded. The cells were then resuspended in a hypotonic buffer (10 mM Tris-HCl pH 7.5, 10 mM KCl, 1.5 mM MgCl_2_, 0.5 mM DTT and 1× protease inhibitor cocktail), and incubated on ice for 15 min followed by centrifugation at 425 g for 10 min. After discarding the supernatant, the pellet was lysed in the lysis buffer 0.3 (50 mM Tris-HCl pH 7.5, 150 mM NaCl, 2 mM MgCl_2_, 0.3% NP40 and 1× protease inhibitor cocktail (Roche)), rotated at 4 °C for 10 min, and then centrifuged at 950 g for 10 min. The obtained supernatant was collected and marked as cytoplasmic fraction. The remaining pellets were resuspended with 1 mL lysis buffer 0.5 (50 mM Tris-HCl pH 7.5, 150 mM NaCl, 2 mM MgCl_2_, 0.5% NP40 and 1× protease inhibitor cocktail), rotated at 4 °C for 10 min, and centrifuged at 950 g for 10 min. The supernatant was discarded and then the remaining pellets were resuspended in 40 µL buffer 1 (50% glycerol, 20 mM Tris-HCl pH 8.0, 75 mM NaCl, 0.5 mM EDTA, 0.85 mM DTT), followed by the addition of 360 µL buffer 2A (20 mM HEPES pH 7.6, 300 mM NaCl, 0.2 mM EDTA, 1 mM DTT, 7.5 mM MgCl_2_, 1M urea, 1% NP-40, adding 400 U RNase inhibitor per mL when used), vortex for 10 s, and rotation at 4 °C for 10 min. After centrifugation for 5 min, the supernatant was collected and marked as nucleoplastic fraction. The remaining pellets were resuspended in 100 µL buffer 1, followed by the addition of 900 µL buffer 2B (20 mM HEPES pH 7.6, 300 mM NaCl, 0.2 mM EDTA, 1 mM DTT, 7.5 mM MgCl_2_, 1 M urea, 1.5% NP-40, adding 400 U RNase inhibitor per mL when used), vortex for 10 s, and rotation at 4 °C for 10 min. After centrifugation at 15,000× *g* for 5 min, the supernatant was discarded. The remaining pellet was washed twice with 600 µL buffer 2A. Finally, the remaining pellets were resuspended with 150 µL buffer 3 (50 mM Tris-HCl pH 7.4, 100 mM NaCl, 0.1% SDS, 0.5% Sodium deoxycholate, adding 400 U RNase inhibitor per mL when used), and the supernatant was collected and marked as chromatin bound fraction.

### Plasmid construction

SGF29 cDNA was generated through reverse transcription of mRNAs from HEK293T cells. DNA fragments encoding the full length human SGF29, SGF29 (1–53), SGF29 (54–293), SGF29 (1–98), SGF29 (99–293) and SGF29 (Δ160–286) were amplified by PCR using Phusion High-Fidelity DNA Polymerase (NEB) and cloned into the pLE4 vector (a gift from Tomoaki Hishida) or the pET28a vector. Generation of SGF29 H3K4me3 binding mutants (D194A, D196A, Y245A, F264A) and R207P mutant was performed through site-directed mutagenesis^43^. eGFP-RPB1 plasmid was a kind gift from Dr. László Tora^42^. Primers used for plasmid construction are listed in Supplementary Table S3.

### Clonal expansion assay

The clonal expansion assay was performed as previously described^81^. In brief, 2000 hMPCs were seeded in a 0.1% gelatin-coated well of a 12-well plate (Corning) and cultured for approximately 2 weeks. The cells were washed twice with cold PBS and then fixed with 4% paraformaldehyde (PFA) for 30 min. After being washed with PBS, the cells were stained with 10% crystal violet (Biohao, C0520) for 30 min at room temperature (RT). Subsequently, crystal violet-stained plates were scanned by a scanner (EPSON V370). The relative cell density was calculated using ImageJ software.

### SA-β-Gal staining

The SA-β-Gal staining of hMPCs was performed as described previously^52,87^. In brief, hMPCs were washed with PBS before fixation with a buffer containing 2% (w/v) formaldehyde and 0.2% (w/v) glutaraldehyde for 5 min. After washing with PBS for twice, the cells were incubated at 37 °C overnight with staining buffer containing 1 mg/mL X-gal. The optical microscope was used to obtain SA-β-Gal-stained cells and the percentage of SA-β-Gal-positive cells was calculated.

### Immunofluorescence

The hMPCs or HEK293T cells were fixed in 4% PFA for 15 min at RT. The cells were washed with PBS and permeabilized in permeabilization buffer for 15 min at RT. Subsequently, the cells were blocked with blocking solution at 37 °C for 30 min. The cells were incubated with the primary antibodies overnight at 4 °C, and then subjected to five washes with 1× PBST buffer. Afterward, the cells were incubated with secondary antibodies and Hoechst 33342 (Thermo Fisher Scientific) for 1 h at RT. The cells were washed with 1× PBST. Finally, coverslips were mounted onto glass slides with Antifade Mounting Medium (Vector Laboratories, H-1000). Images were taken with ZEISS confocal LSM900, using a 63× HC Plan-Apochromat, numerical aperture (NA) 1.40 oil-immersion objective, and laser wavelengths of 405 nm at 0.6%, 488 nm at 4%, 568 nm at 3.3%, and 640 nm at 2%. The same laser parameters were applied to all groups. Puncta analysis were conducted as previously described^33,88^. A size at or above 0.196 µm^2^ (area) was considered to be a punctum in the quantitative analyses.

The antibodies used for immunofluorescence staining were as follow: anti-SGF29 (Santa Cruz, sc-515286); anti-P21^Cip1^ (Cell signaling Technology, CST2974s); anti-HP1α (Cell signaling technology, CST2616s); anti-Ki67 (ZSGB-BIO, ZM-0166); anti-γH2A.X (Millipore, 05-636); anti-SP1 (Abcam, ab231778); Hoechst 33342 (Thermo Fisher Scientific); Anti-AT7L3 (HuaBio, ER60495); Anti-ATXN7L2 (HuaBio, ER1904-03); Anti-TAF5L (HuaBio, ER65092); anti-SF3B3 (Santa cruz, sc-398670); anti-SUPT20H (Santa cruz, sc-374665).

### Chemical treatment

1, 6-hexanediol (Sigma, 240117) treatment was performed as previously described^17^. In brief, 10% 1, 6-hexanediol was dissolved in PBS. hMPCs were seeded in a 0.1% gelatin-coated 6-well plate with a glass coverslip in each well for 24 h. Then, the cells were treated with the 10% 1, 6-hexanediol for 1 min and fixed with 4% PFA and processed for immunofluorescence staining.

### Live-cell imaging

For live-cell imaging, the hMPCs were seeded at a density of 2 × 10^5^ per well in glass bottom dishes (Cellvis, D35-20-1.5-N) 48 h post-infection with lentivirus carrying EGFP-SGF29. After 24 h, live cell imaging was carried out at various time points using a Andor Dragonfly 505 fluorescence microscopy with a temperature-controlled heating system. The cells were imaged with a 60× oil objective lens and images were acquired every 2.5 min for 24 h period.

### FRAP analysis

For FRAP experiments, the hMPCs infected with lentiviruses expressing either EGFP-SGF29 WT or R207P for 96 h were plated on glass bottom dishes (Cellvis, D35-20-1.5-N), and then imaged using a Zeiss LSM900 microscope. Five control images were taken before bleaching. A punctum was selected and bleached with 10 times at nominal 100% laser transmission (488-nm laser). Under these settings, approximately 70%–80% of the signal was bleached. Time-lapse images were collected every one second^89^. The fluorescence intensities of the puncta were measured using Zeiss software.

### Recombinant protein purification

To express recombinant full length SGF29 or SGF29-(54–293) proteins in bacteria, the pET28a vector containing a 6× His tag and encoding sequences of SGF29 full length, and SGF29 C terminal truncation variants (SGF29-C-WT, SGF29-C-D194A, SGF29-C-R207P) was transformed into Rosetta 2 (DE3) competent cells. One litre of bacterial cultures was grown at 37 °C for 8 h followed by addition with a final concentration of 1 mM isopropyl-β-d-1-thiogalactopyranoside at 28 °C for 16 h. After the induction, the cells were harvested and lysed in lysis buffer (8 M urea, 100 mM NaH_2_PO_4_, 10 mM Tris-HCl pH 8.0, 20 mM imidazole) for 30 min on the ice. After sonication, lysates were centrifuged at 13,000× *g* for 1 h. The supernatants were passed through Ni-column (Qiagen, 30250) and washed sequentially with the washing buffers (8 M urea, 100 mM NaH_2_PO_4_, 10 mM Tris-HCl pH 6.3, 20 mM imidazole). The His-tagged fusion proteins were then eluted in elution buffer (8 M urea, 100 Mm NaH_2_PO_4_, 10 mM Tris-HCl pH 4.0, 250 mM imidazole). The proteins were eluted by adding 5× SDS loading buffer, boiled, and analyzed by SDS-PAGE. The fractions were dialyzed with solution from 8 M urea to 0 M urea and concentrated to reach a final protein concentration of 1 M in 25 mM Tris-HCl pH 7.5 and stored at −80 °C.

### Co-IP

The co-IP experiments were carried out as previously described^78,86^. In Brief, Flag-EGFP and Flag-EGFP-SGF29 WT or R207P plasmids were transfected into HEK293T cells using lipofectamine^TM^ 3000 (Thermo Fisher, L3000015). The cells were harvest and lysed in CHAPS lysis solution (0.3% CHAPS, 40 mM HEPES pH 7.5, 1 mM EDTA, 120 mM NaCl, and 1× protease inhibitor cocktail (Roche)) on ice for 2 h. After centrifugation at 13,000× *g* for 30 min at 4 °C, the supernatants were collected and mixed with anti-Flag M2 affinity gel (Sigma, A2220), and rotated at 4 °C overnight. After centrifugation at 500× *g* for 2 min, bead-bound proteins were eluted with Flag peptides for 2 h and detected by immunoblotting using indicated antibodies.

### RNA-seq data processing

RNA-seq data processing pipeline has been described previously^90^. Initially, low-quality reads and adapters were trimmed using TrimGalore software (version 0.6.7). Clean reads were then mapped to the UCSC human hg19 genome using HISAT2 software (version 2.2.1)^91^. The read counts for each gene were generated by featureCounts (version 2.0.1). FPKM (Fragments Per Kilobase per Million) for each gene was calculated using custom scripts. Identification of DEGs were performed using R/Bioconductor package DESeq2^92^ with a cutoff Benjamini Hochberg adjust *P*-value less than 0.05 and absolute log_2_(fold change) > 0.58. GO term enrichment analysis was performed using Metascape (https://metascape.org/). The DEGs are listed in Supplementary Table S4.

### ChIP-qPCR and ChIP-seq

1 × 10^7^ hMPCs were harvest and crosslinked with 1% (v/v) formaldehyde diluted in cold PBS for 10 min at RT, and quenched by incubating in 1.25 M Glycine for 5 min at RT. After being washed with PBS, the cells were resuspended in ice-cold lysis buffer (400 mM NaCl, 20 mM Tris-HCl pH 7.4, 0.1% NP-40, 0.5 mM EDTA, 1.5 mM MgCl_2_) on ice for 30 min. After sonication by a Bioruptor^®^ Plus device (Diagenode), the supernatant was transferred to a new tube and centrifuged at 13,000 rpm at 4 °C for 10 min. Subsequently, the supernatant was collected and added with 1.6 times volume of NETN-0 buffer (20 mM Tris-HCl pH 7.4, 0.1% NP-40, 1.5 mM MgCl_2_). Subsequently, the samples were incubated at 4 °C overnight with Protein A/G dynabeads (Thermo Fisher Scientific, 10004D) conjugated with 2.4 μg rabbit/mouse IgG or anti-Flag (F1804, Sigma), respectively. Then, the beads were washed five times with NETN-150 buffer (150 mM NaCl, 20 mM Tris-HCl pH 7.4, 0.1% NP-40, 0.5 mM EDTA, 1.5 mM MgCl_2_), and reverse cross-linking were performed with proteinase K at 65 °C overnight. DNA was purified by the phenol-chloroform-isoamylalcohol DNA isolation method and subjected to qPCR for evaluation of SP1, MED4, Pol II S2 or SGF29 occupation at *CDKN1A* promoter sequences. The primers used for ChIP-qPCR are listed in Supplementary Table S3. In addition, we performed FLAG-mediated ChIP-seq of SGF29-WT, SGF29-D194A or SGF29-R207P, and the DNA fragments were used to construct the library via KAPA Hyper Prep Kits with PCR Library Amplification/Illumina series (KK8504, Roche) for subsequent analyses.

### ChIP-seq data processing

The ChIP-seq processing pipeline has been previously described^93^. In brief, clean reads were generated using TrimGalore software and were aligned to UCSC human hg19 genome using bowtie2 (version 2.4.4)^94^. The mapped reads with low mapping quality (< 10) and duplication were then filtered using samtools (version 1.9) and Picard (version 2.18.29). To minimize the bias caused by sequencing depth, replicates for each sample were merged and the same numbers (61 million reads) of high-quality reads were randomly sampled for merged bam files. The ChIP-seq signals were calculated for each 100-bp bin size by deepTools (version 3.5.1)^95^ and were then processed using Z-score normalization for epigenomic signal visualization.

The broad peak calling was carried out using MACS2^96^ (version 2.1.2) with parameter “--broad -g hs --broad-cutoff 0.001 -p 0.001”^96^. The identification of differential peaks was performed by overlapping peak regions between different conditions and classifying peaks as unique to one condition^97^. The genomic locations of SGF29 ChIP-seq peaks are presented in Supplementary Table S5.

### FISH with immunofluorescence

The cells were seeded on poly-(D-lysine)-coated (Sigma, P6407) coverslips and cultured overnight followed by fixation using 4% PFA for 15 min. Next, the fixed cells were washed three times with PBS, permeabilized by 0.5% Triton X-100 in PBS for 10 min and blocked with 10% donkey serum for 30 min. The cells were incubated with primary antibodies of anti-SGF29 or anti-MED4 in 1× PBST buffer containing 10% donkey serum at 4 °C overnight and incubated with secondary antibodies (Thermo Fisher Scientific) in 1× PBST buffer for 1 h at RT. Finally, the cells were washed three times with PBS and post-fixed with 4% PFA for 15 min.

For DNA FISH, a Cy3-labeled secondary probe coupled with unlabeled primary probes was used as previously described^98^. 239 primary probes (Supplementary Table S3) covering the promoter of *CDKN1A* were designed with PaintSHOP^93^. To initiate the Primer Exchange Reactions (PERs), the following components were added to a reaction volume of 90 µL: 10 µL of 10× PBS, 20 µL of hairpin (10 μM), 10 µL of 100 mM MgSO_4_, 10 µL of dNTP (6 mM/each), 10 µL of 1 µM Clean G, 10 µL of Bst LF polymerase (NEB, M0275L), and the remaining 20 µL of the reaction volume was filled with double-distilled water (ddH_2_O). The mixture was incubated at 37 °C for 20 min, followed by the addition of 10 µL of the primer probe with a concentration of 10 µM. The reaction was then incubated at 37 °C for 1.5 h. Finally, the polymerase was inactivated by a 20-min reaction at 80 °C. The above immunofluorescence (IF)-treated cells were incubated in 0.2 M HCl for 5 min and washed in 2× SSCT (2× SSC containing 0.2% Tween-20) for 1 min. After being incubated in 2× SSCT for 5 min, the cells were transferred to fresh 2× SSCT at 60 °C for 1 h. 250 μL in situ hybridization (ISH) reaction mixture containing 62.5 μL of 4× FISH Master Mix, 125 μL of 100% formamide, 20 μL of PER extension solutions, 2.5 μL of 100 mg/mL RNase A (Tiangen, RT405-12), and 40 μL DEPC-treated ddH_2_O was prepared. After denaturation at 85 °C for 5 min, samples were incubated with 250 μL ISH reaction mixture at 43 °C at least 16 h in a humidified incubator. Subsequently, the hybridization mixture was discarded and the cells were washed five times with 2× SSCT at 60 °C for 5 min. Probe (5′Cy3-TTGTTAAGTTGTGTTAAGTTGT3′) for fluorescent hybridization was directly incubated with cells at 37 °C for 1 h. Subsequently, the cells were rinsed twice with PBS. After being stained with Hoechst 33342 for 5 min, the cells were washed with PBS. Finally, coverslips were mounted onto glass slides with Anti-fade Mounting Medium.

For RNA FISH, the hybridized flap-structured duplexes were prepared in a PCR machine as described previously^99,100^. The above IF-treated cells were incubated in 15% formamide freshly prepared in SSC buffer for 15 min at RT. Subsequently, 50 µL of the hybridization mixture were added on a 10 cm petri dish and incubate at 37 °C overnight. The cells were rinsed twice for 30 min in freshly prepared 1× SSC solution containing 15% formamide at 37 °C. Finally, the cells were washed with PBS twice and coverslips were mounted onto glass slides with Anti-fade Mounting Medium.

### Statistical analysis

Statistical analyses were performed using Graph-Pad Prism Software (GraphPad Software). Data are presented as mean ± SEM. Comparisons were performed with Two-tailed unpaired Student’s *t-*test. *P* value < 0.05 was defined as statistically significant. *P < 0.05, **P < 0.01, and ***P < 0.001.

## Supplementary information

Supplementary Figures

Supplementary Video S1

Supplementary Video S2

Supplementary Video S3

Supplementary Video S4

Supplementary Table S1

Supplementary Table S2

Supplementary Table S3

Supplementary Table S4

Supplementary Table S5

## Data Availability

The high-throughput sequencing data including RNA-seq and ChIP-seq generated in this study have been deposited in the Genome Sequence Archive (GSA) in the National Genomics Data Center, Beijing Institute of Genomics (China National Center for Bioinformation) of the Chinese Academy of Sciences under the accession number HRA003829, which are publicly accessible at http://bigd.big.ac.cn/gsa-human. Other data or materials generated in this study are available from the corresponding authors upon reasonable request.
