# Publisher Correction: SGF29 nuclear condensates reinforce cellular aging

**DOI:** 10.1038/s41421-024-00690-z

**Published:** 2024-05-30

**Authors:** Kaowen Yan, Qianzhao Ji, Dongxin Zhao, Mingheng Li, Xiaoyan Sun, Zehua Wang, Xiaoqian Liu, Zunpeng Liu, Hongyu Li, Yingjie Ding, Si Wang, Juan Carlos Izpisua Belmonte, Jing Qu, Weiqi Zhang, Guang-Hui Liu

**Affiliations:** 1 State Key Laboratory of Membrane Biology, Institute of Zoology, Chinese Academy of Sciences, Beijing, China; 2 University of Chinese Academy of Sciences, Beijing, China; 3 Institute for Stem Cell and Regeneration, Chinese Academy of Sciences, Beijing, China; 4 Beijing Institute for Stem Cell and Regenerative Medicine, Beijing, China; 5 Shanghai Institute of Materia Medica, Chinese Academy of Sciences, Shanghai, China; 6 State Key Laboratory of Stem Cell and Reproductive Biology, Institute of Zoology, Chinese Academy of Sciences, Beijing, China; 7 CAS Key Laboratory of Genomic and Precision Medicine, Beijing Institute of Genomics, Chinese Academy of Sciences and China National Center for Bioinformation, Beijing, China; 8 National Laboratory of Biomacromolecules, CAS Center for Excellence in Biomacromolecules, Institute of Biophysics, Chinese Academy of Sciences, Beijing, China; 9 Advanced Innovation Center for Human Brain Protection, and National Clinical Research Center for Geriatric Disorders, Xuanwu Hospital Capital Medical University, Beijing, China; 10 Aging Translational Medicine Center, International Center for Aging and Cancer, Xuanwu Hospital, Capital Medical University, Beijing, China; 11 Altos Labs, Inc., San Diego, CA USA; 12 Sino-Danish College, University of Chinese Academy of Sciences, Beijing, China

**Keywords:** Cell biology, Senescence

Correction to: *Cell Discovery* (2023) 9:110

10.1038/s41421-023-00602-7 published online 07 November 2023

During the production, we misupdated Supplementary Fig. S8 in the article^1^. The original Supplementary Figures have been corrected. We apologize for any inconvenience that it may have caused.

### Supplementary information

Supplementary Figures
