# Author Correction: SGF29 nuclear condensates reinforce cellular aging

**DOI:** 10.1038/s41421-025-00773-5

**Published:** 2025-02-15

**Authors:** Kaowen Yan, Qianzhao Ji, Dongxin Zhao, Mingheng Li, Xiaoyan Sun, Zehua Wang, Xiaoqian Liu, Zunpeng Liu, Hongyu Li, Yingjie Ding, Si Wang, Juan Carlos Izpisua Belmonte, Jing Qu, Weiqi Zhang, Guang-Hui Liu

**Affiliations:** 1 State Key Laboratory of Membrane Biology, Institute of Zoology, Chinese Academy of Sciences, Beijing, China; 2 University of Chinese Academy of Sciences, Beijing, China; 3 Institute for Stem Cell and Regeneration, Chinese Academy of Sciences, Beijing, China; 4 Beijing Institute for Stem Cell and Regenerative Medicine, Beijing, China; 5 Shanghai Institute of Materia Medica, Chinese Academy of Sciences, Shanghai, China; 6 State Key Laboratory of Stem Cell and Reproductive Biology, Institute of Zoology, Chinese Academy of Sciences, Beijing, China; 7 CAS Key Laboratory of Genomic and Precision Medicine, Beijing Institute of Genomics, Chinese Academy of Sciences and China National Center for Bioinformation, Beijing, China; 8 National Laboratory of Biomacromolecules, CAS Center for Excellence in Biomacromolecules, Institute of Biophysics, Chinese Academy of Sciences, Beijing, China; 9 Advanced Innovation Center for Human Brain Protection, and National Clinical Research Center for Geriatric Disorders, Xuanwu Hospital Capital Medical University, Beijing, China; 10 Aging Translational Medicine Center, International Center for Aging and Cancer, Xuanwu Hospital, Capital Medical University, Beijing, China; 11 Altos Labs, Inc., San Diego, CA USA; 12 Sino-Danish College, University of Chinese Academy of Sciences, Beijing, China

**Keywords:** Cell biology, Senescence

Correction to: *Cell Discovery* 10.1038/s41421-023-00602-7, published online 07 November 2023

In this article, we identified an oversight in Fig. 5g^1^, where the red and green channel images were inadvertently reversed. Also, Supplementary Fig. S7d contained an oversight with the selection of an atypical nucleus for EGFP-SGF29-WT group.

Incorrect Figure 5

**Fig. 5:**
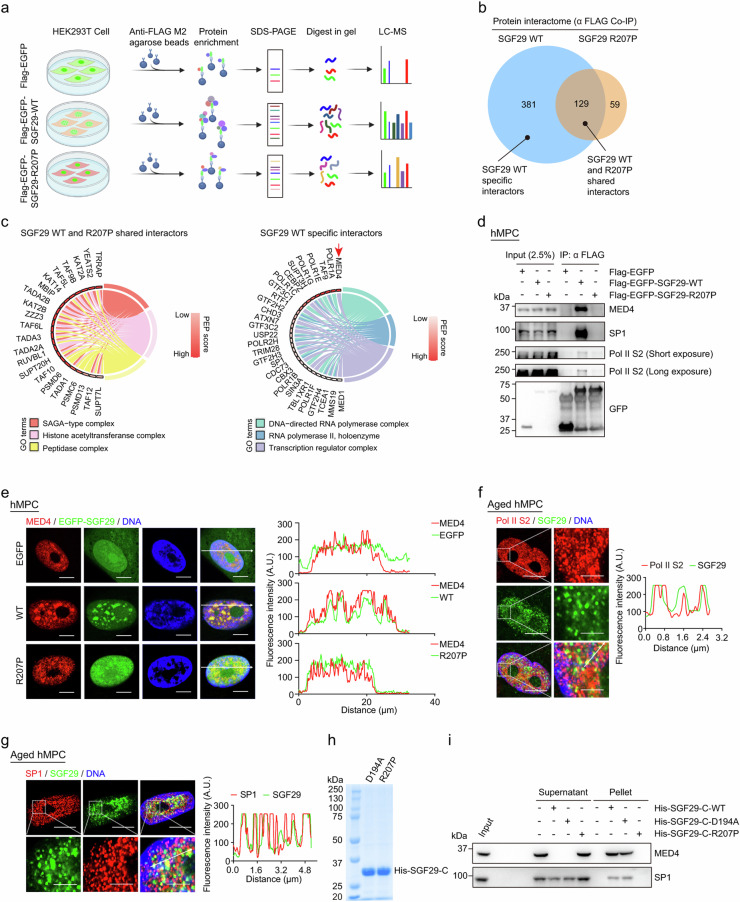
Identification of SGF29 interacting proteins sensitive to condensate perturbation. **a** Flow chart of the mass spectrometry strategy for the identification of EGFP-SGF29-WT (WT) and EGFP-SGF29-R207P (R207P) interacting proteins. Flag-EGFP was used as a control. **b** Shared and specific interacting proteins of EGFP-SGF29-WT (WT) and EGFP-SGF29-R207P (R207P) identified by mass spectrometry. **c** Chord diagrams showing the enriched pathways of shared interaction partners of EGFP-SGF29-WT (WT) and EGFP-SGF29-R207P (R207P) (left) and those of specific protein interaction partners of EGFP-SGF29-WT (right). **d** Co-IP analysis showing the interaction between indicated proteins and EGFP-SGF29-WT (WT) and EGFP-SGF29-R207P (R207P) in hMPCs. **e** Immunofluorescence staining of MED4 in hMPCs transduced with lentiviruses expressing either EGFP, EGFPSGF29-WT (WT) or EGFP-SGF29-R207P (R207P). Left, representative images. Scale bar, 10 μm. Right, quantification of the fluorescence intensity along the line embedded the image following the arrow direction. **f** Immunofluorescence staining of Pol II S2 and SGF29 in senescent hMPCs. Left, representative images. Scale bars, 10 μm and 5 μm (zoomed-in image). Right, quantification of the fluorescence intensity along the line embedded the image following the arrow direction. **g** Immunofluorescence staining of SP1 and SGF29 in senescent hMPCs. Left, representative images. Scale bars, 10 μm and 5 μm (zoomed-in image). Right, quantification of the fluorescence intensity along the line embedded the image following the arrow direction. **h** Coomassie blue staining of purified recombinant SGF29-C-D194A and SGF29-C-R207P after being resolved on SDS-PAGE. **i** Pelleting assay show that SGF29-C-D194A and SGF29-C-R207P interact with MED4 and SP1.

Correct Figure 5

**Fig. 5:**
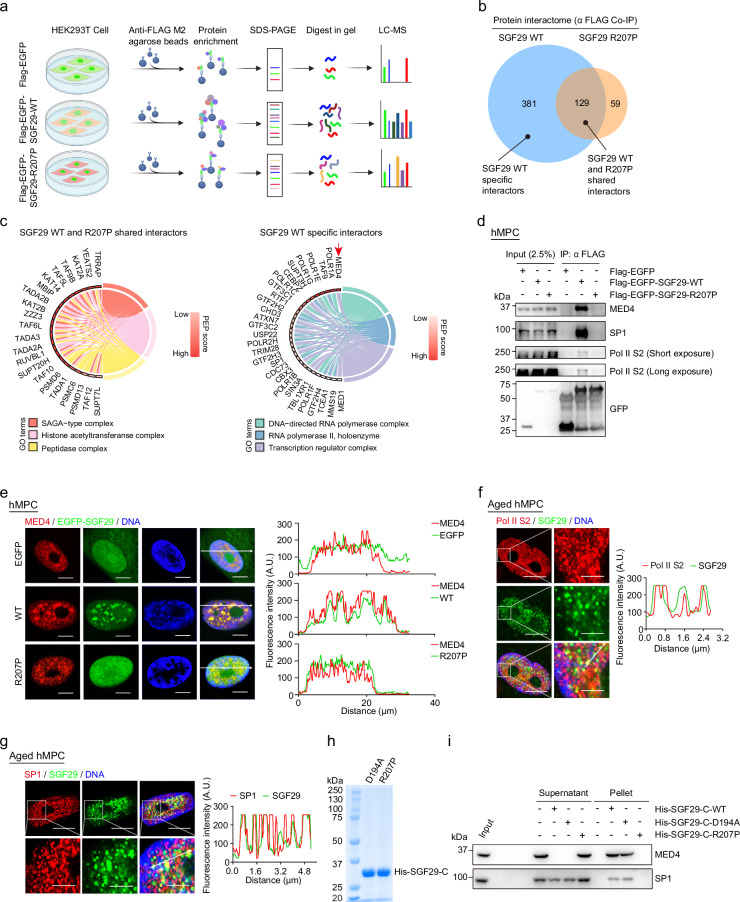
Identification of SGF29 interacting proteins sensitive to condensate perturbation. **a** Flow chart of the mass spectrometry strategy for the identification of EGFP-SGF29-WT (WT) and EGFP-SGF29-R207P (R207P) interacting proteins. Flag-EGFP was used as a control. **b** Shared and specific interacting proteins of EGFP-SGF29-WT (WT) and EGFP-SGF29-R207P (R207P) identified by mass spectrometry. **c** Chord diagrams showing the enriched pathways of shared interaction partners of EGFP-SGF29-WT (WT) and EGFP-SGF29-R207P (R207P) (left) and those of specific protein interaction partners of EGFP-SGF29-WT (right). **d** Co-IP analysis showing the interaction between indicated proteins and EGFP-SGF29-WT (WT) and EGFP-SGF29-R207P (R207P) in hMPCs. **e** Immunofluorescence staining of MED4 in hMPCs transduced with lentiviruses expressing either EGFP, EGFPSGF29-WT (WT) or EGFP-SGF29-R207P (R207P). Left, representative images. Scale bar, 10 μm. Right, quantification of the fluorescence intensity along the line embedded the image following the arrow direction. **f** Immunofluorescence staining of Pol II S2 and SGF29 in senescent hMPCs. Left, representative images. Scale bars, 10 μm and 5 μm (zoomed-in image). Right, quantification of the fluorescence intensity along the line embedded the image following the arrow direction. **g** Immunofluorescence staining of SP1 and SGF29 in senescent hMPCs. Left, representative images. Scale bars, 10 μm and 5 μm (zoomed-in image). Right, quantification of the fluorescence intensity along the line embedded the image following the arrow direction. **h** Coomassie blue staining of purified recombinant SGF29-C-D194A and SGF29-C-R207P after being resolved on SDS-PAGE. **i** Pelleting assay show that SGF29-C-D194A and SGF29-C-R207P interact with MED4 and SP1.

While these issues do not impact the results or conclusions, we are committed to the highest standards of publication integrity. The original figure and Supplementary figures have been corrected. We apologize for any inconvenience that it may have caused.

## Supplementary information

Supplementary information
