# Supplementary material for: SGF29 nuclear condensates reinforce cellular aging
Source: Cell Discov. 2023 Nov 7;9:110. doi: 10.1038/s41421-023-00602-7 (PMC10630320; doi:10.1038/s41421-023-00602-7)
Supplement: Supplementary file 1 — Supplementary Figures [file 41421_2023_602_MOESM1_ESM.pdf]

Supplementary information

Supplementary Fig. S1

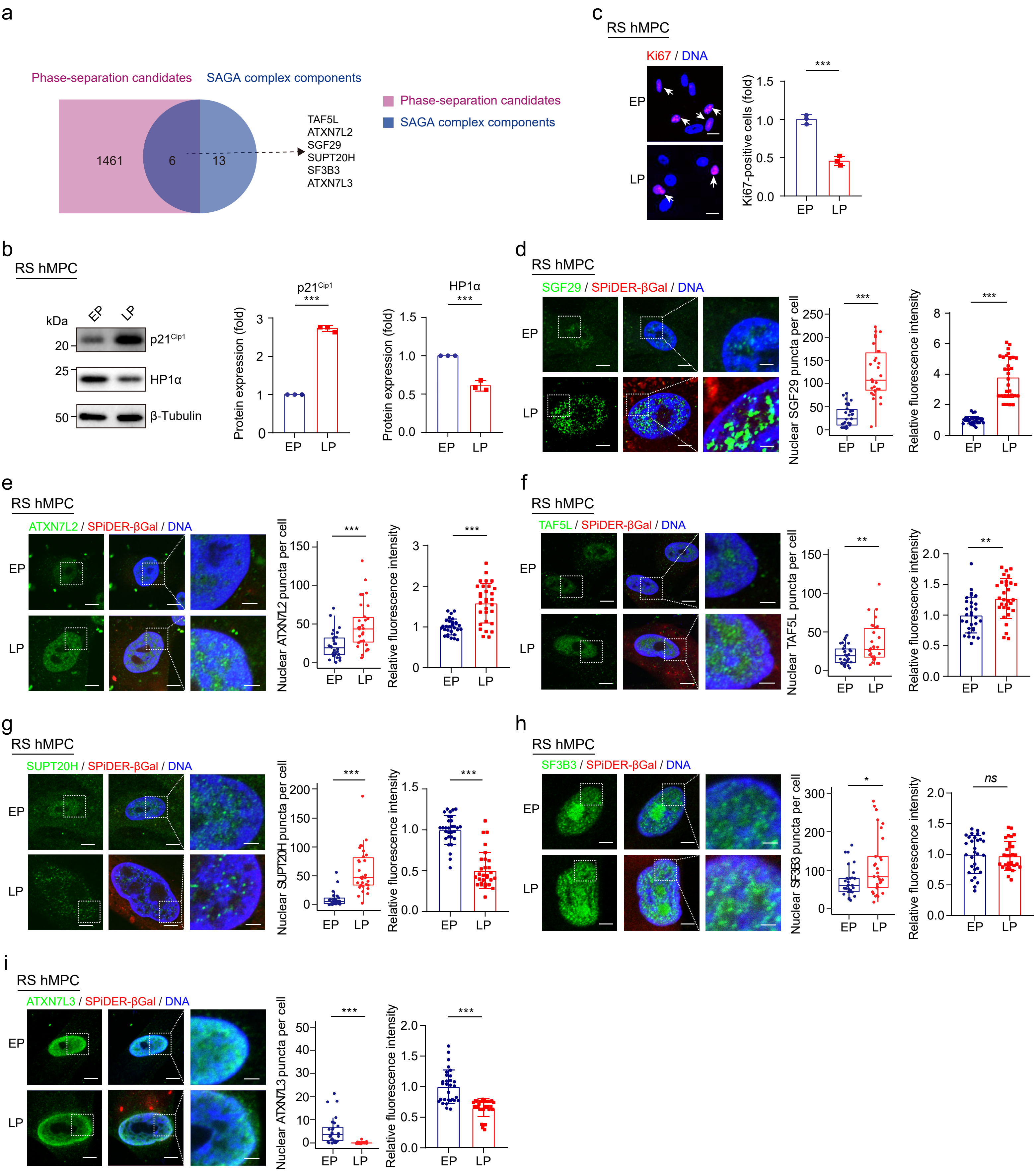

Supplementary Fig. S2

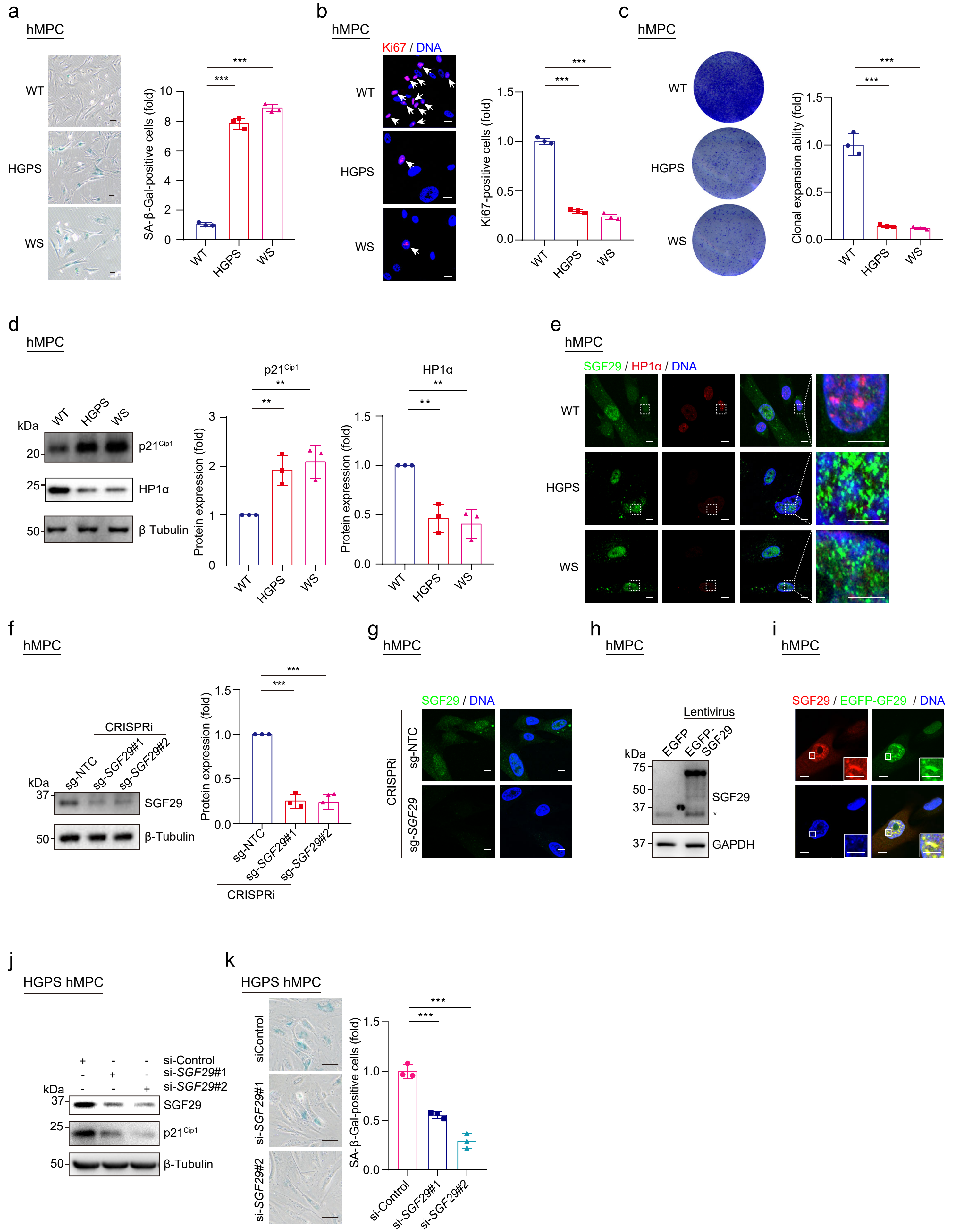

Supplementary Fig. S3

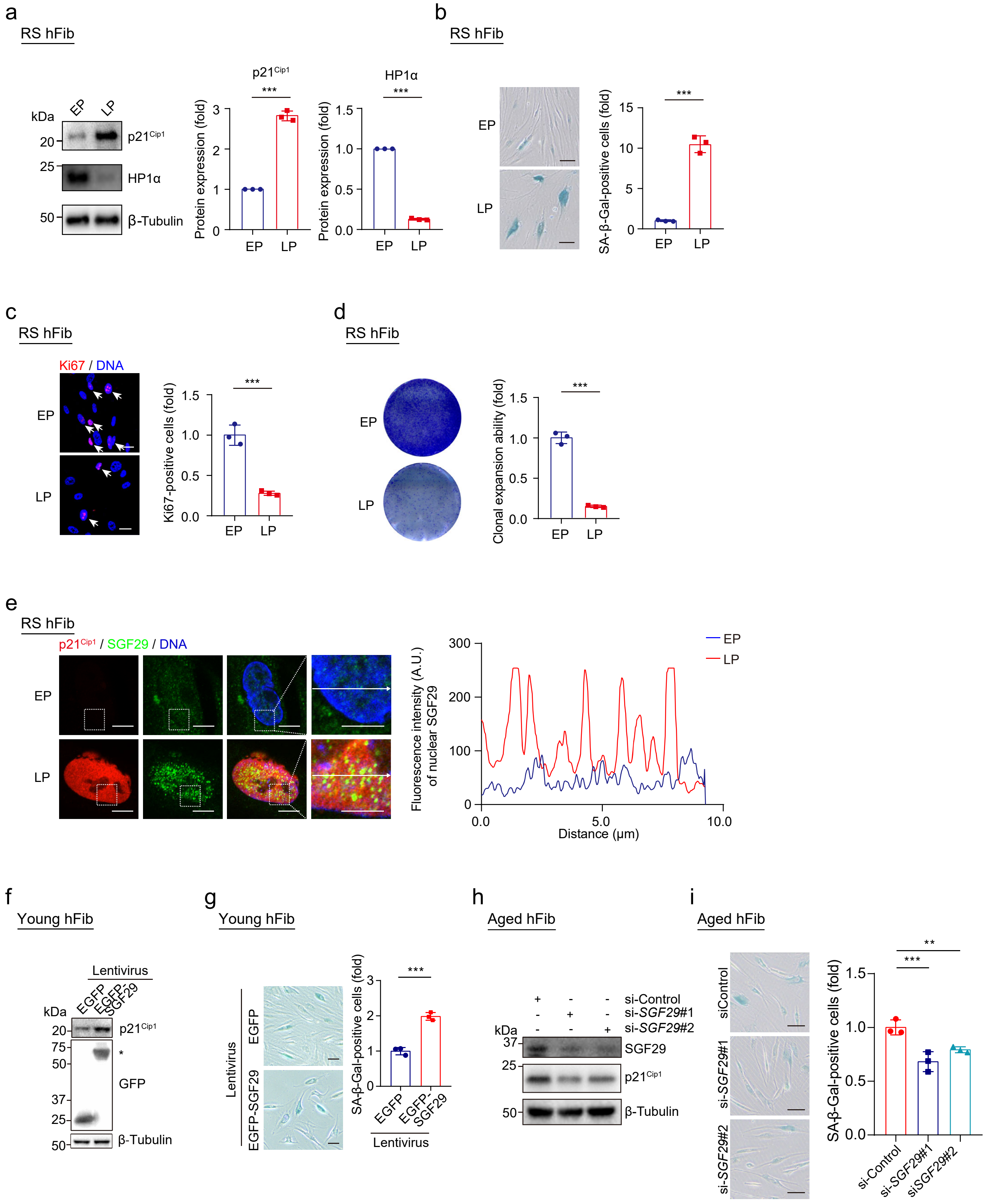

Supplementary Fig. S4

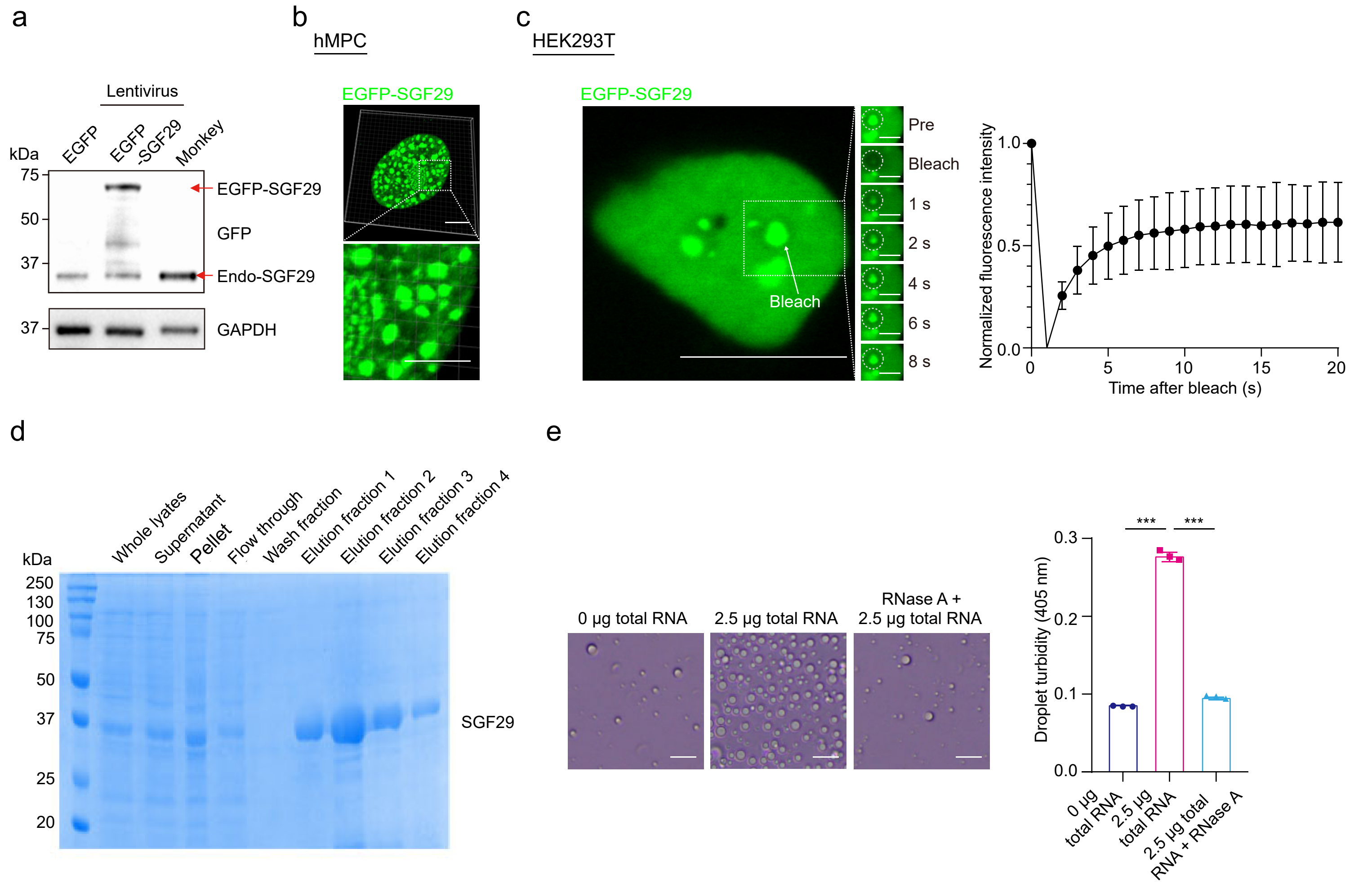

Supplementary Fig. S5

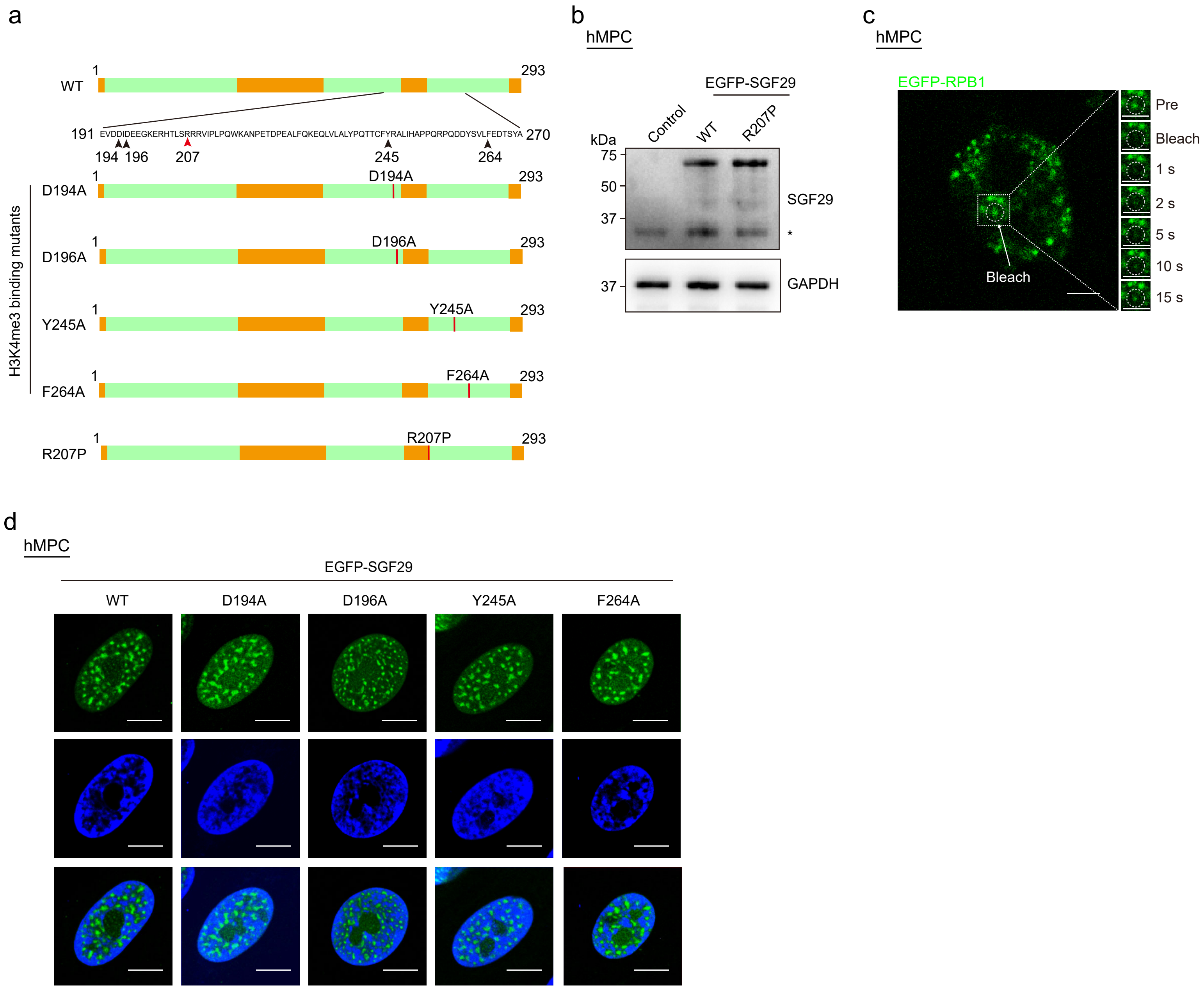

Supplementary Fig. S6

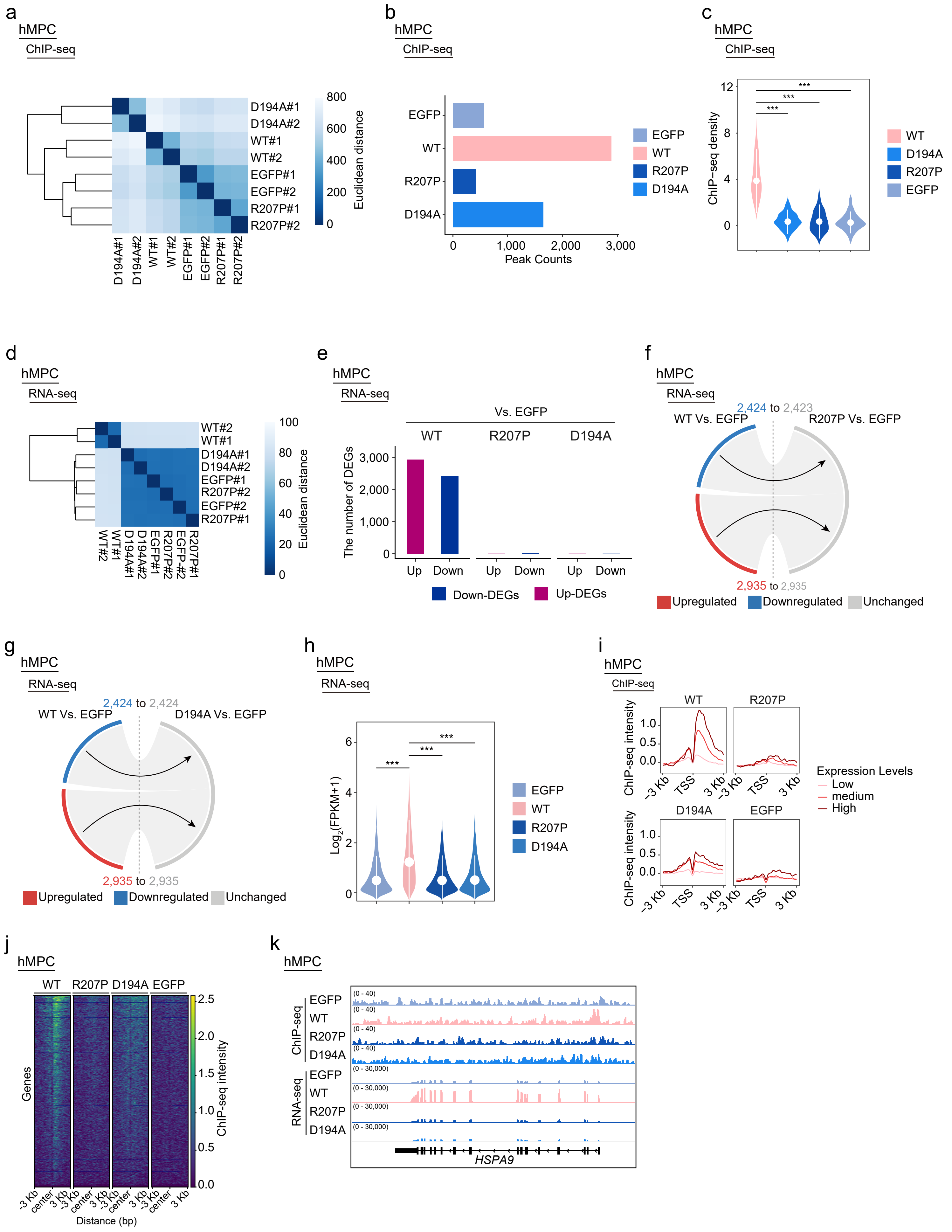

Supplementary Fig. S7

a

Type	Protein name	Coverage (%)	Unique peptide sequences
SGF29 WT and R207P shared interactors	KAT2A	22%	ELKDPDQLYTTLK<br>EYNPPDSEYCR<br>KLFVADLQR<br>LETPAQFR<br>LFVADLQR<br>LGVFSACK<br>IPYTELSHIIKK<br>MDLQQPAANLSELCR<br>NLLAQIK<br>QIPVESVPGIR<br>SCEHPLADHVSHLENVSEDEINR<br>SHPSAWPFMEPVKK<br>SQAEDVATYK<br>TLILTHFPK<br>TLPENLTLEDAKR<br>VIGGICFR<br>YETTHVFGR
	ADA2A	15%	KIYDFLIR<br>LGSFSNDPSDKPPCR<br>IVGPVEHDKFIESHALEFELR<br>LVPGAYLEYK<br>IYDFLIR<br>TAGITNFCSAR
	ADA3	14%	DCPLQFHDFK<br>SEAQHEQPEDGCPFGALTQR<br>VLEAETQILTDWQDK<br>WAQEDLLEEQKDGAR
SGF29 WT specific interactors	MED4	53%	DGEFQELMK<br>DSDIQQLQK<br>EAEQILATAVYQAK<br>ERLLSALEDLEVLSR<br>KGAISSEEIIK<br>LGGGLGVAGGNSTR<br>IHHEMQVLEK<br>IHHEMQVLEKEVEKR<br>LLQAGEENQVLELLIHR<br>LLSALEDLEVLSR<br>ISASNAVCAPLTWVPGDPR<br>RPYPTDLEMR
	GTF2H2	2%	ATIEDILFK
	GTF2H3	7%	LAVIASHIQESR
	GTF2H4	9%	LYGHPATCLAVFR
	SP1	1%	FACPECPK

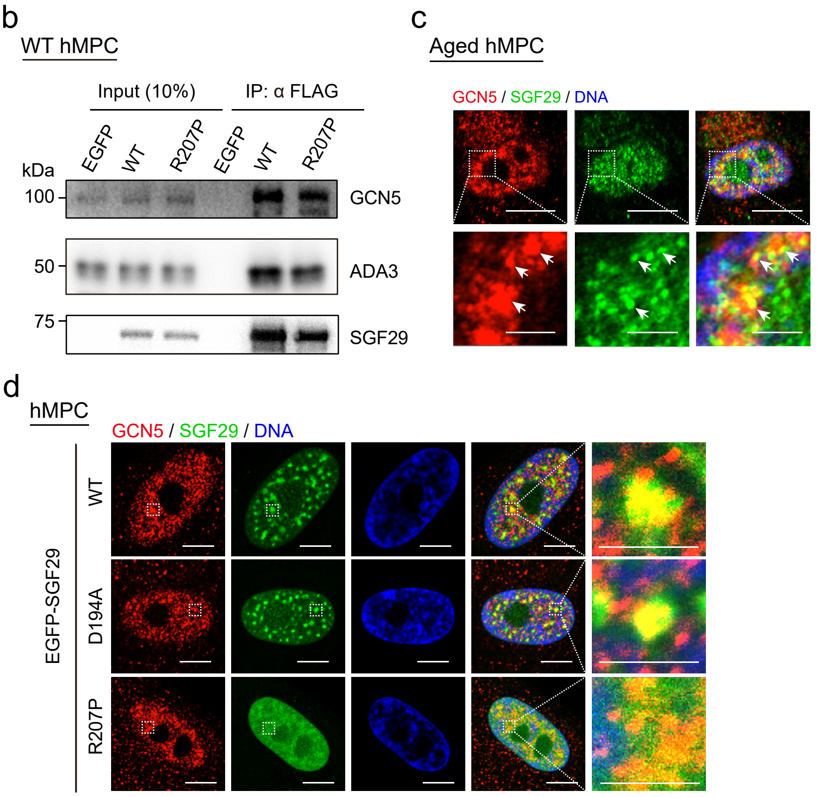

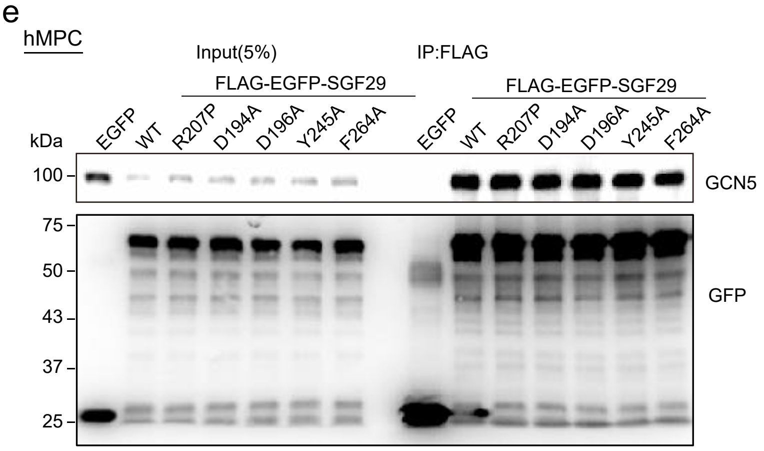

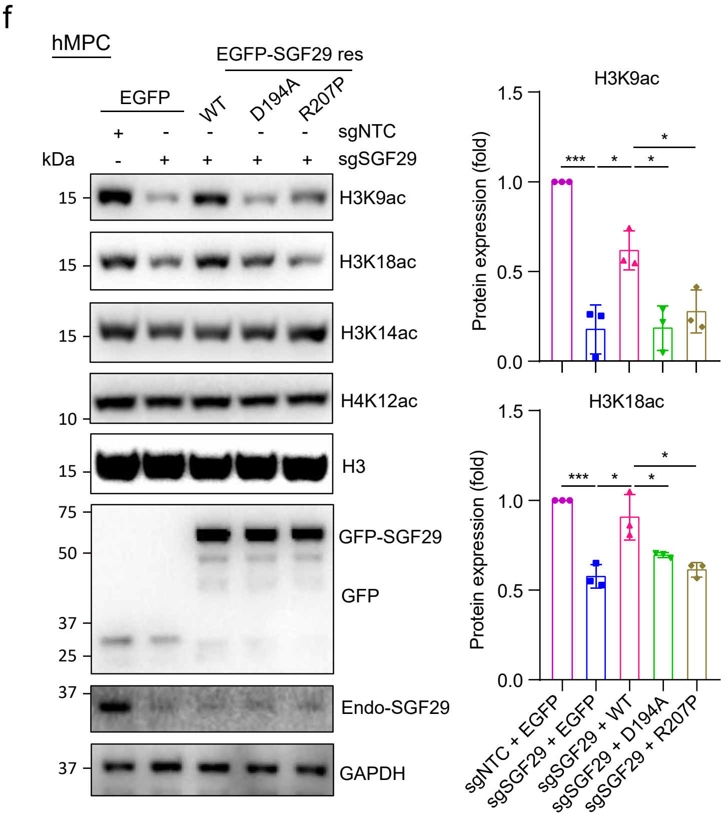

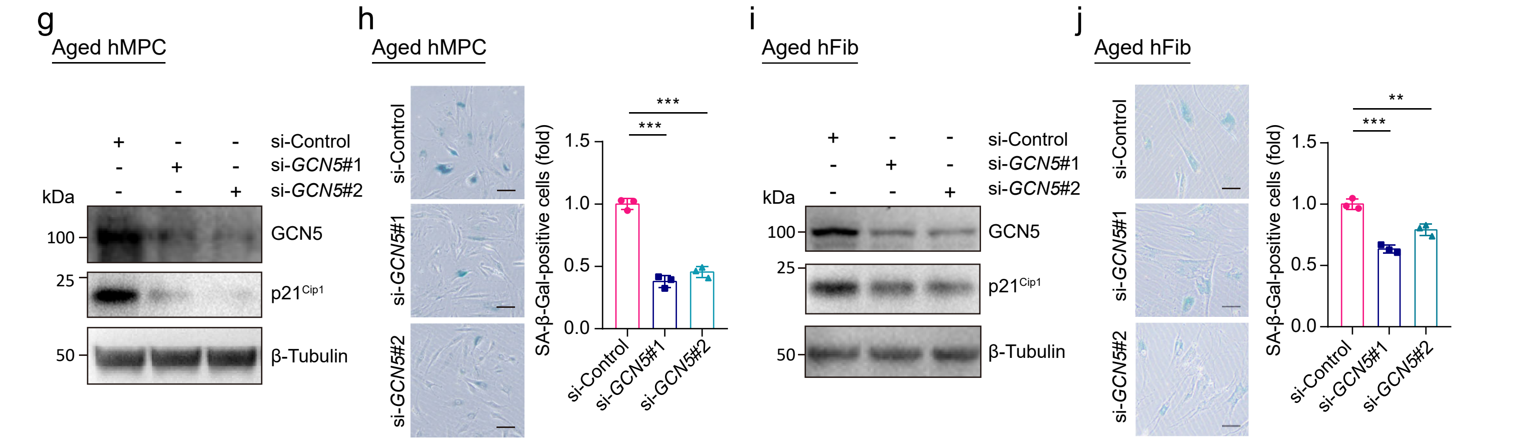

Supplementary Fig. S8

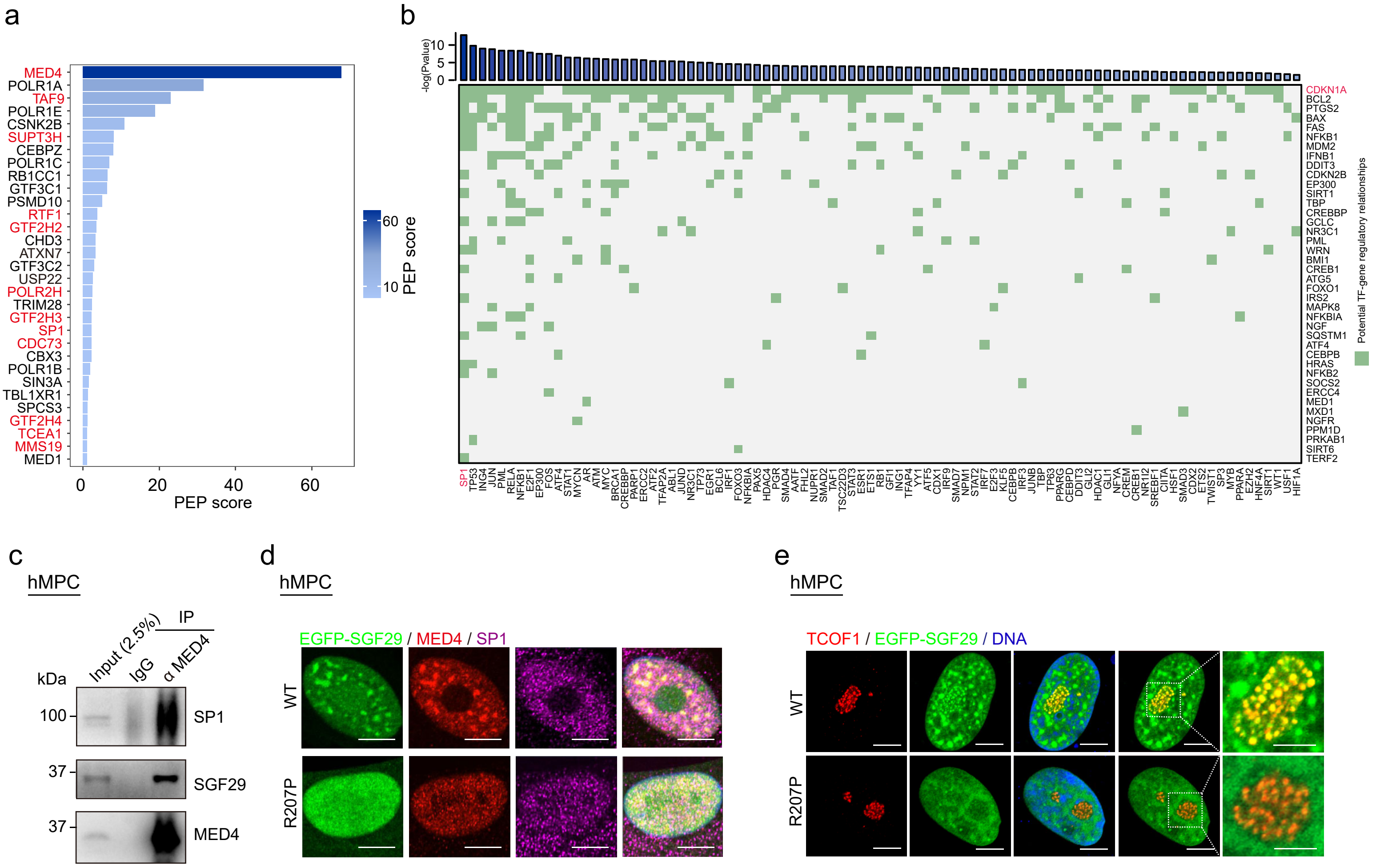

Supplementary Fig. S1. Immunofluorescence staining of SAGA complex members in hMPCs at EP (P5) and LP (P16).

(a) Venn analysis of SAGA complex and phase separation candidates list and showed that a total of 6 members of SAGA complex including TAF5L, SUPT20H, SGF29, ATXN7L2, ATXN7L3, and SF3B3 had potential phase separation capabilities.

(b) Western blotting of HP1α, $p21^{Cip1}$ in WT hMPCs at EP (P5) and LP (P16). Left, representative western blot images. β-Tubulin was used as a loading control. Right, statistical analysis the relative protein expression of HP1α, $p21^{Cip1}$, and SGF29 proteins. Data are presented as the mean ± SEM. n = 3 biological replicates; *ns*, not significant; ***$p < 0.001$ (t test).

(c) Immunofluorescence staining of Ki67 in WT hMPCs at EP (P5) and LP (P16). Left, representative images of immunofluorescence staining. Scale bars: 20 μm. Images were taken with ZEISS confocal LSM900, using a 63× HC Plan-Apochromat, NA 1.40 oil-immersion objective, and laser wavelengths of 405 nm at 0.6%, 568 nm at 3.3%. Right, statistical analysis of the relative percentages of Ki67-positive cells. Data are presented as the mean ± SEM. n = 3 biological replicates. Over 100 cells were quantified in each replicate. ***$p < 0.001$ (t test).

(d) Co-immunostaining of SPIDER-β-gal and SGF29 in hMPCs at EP (P5) and LP (P16). Left: Representative images. Images were taken with ZEISS confocal LSM900, using a 63× HC Plan-Apochromat, NA 1.40 oil-immersion objective, and laser wavelengths of 405 nm at 0.6%, 488 nm at 4%, 568 nm at 3.3%. Scale bars: 10 μm and 2.5 μm (zoomed-in image). Middle: The number of ATXN7L3 puncta in hMPCs at EP (P5) and LP (P16). Right: Quantification of the fluorescence intensity of nuclear ATXN7L3 in hMPCs at EP (P5) and LP (P16). (n = 30 hMPCs). Data are shown as means ± SEM. ***$p < 0.001$ (t test).

(e) Co-immunostaining of SPIDER-β-gal and ATXN7L2 in hMPCs at EP (P5) and LP (P16). Left: Representative images. Images were taken with ZEISS confocal LSM900, using a 63× HC Plan-Apochromat, NA 1.40 oil-immersion objective, and laser wavelengths of 405 nm at 0.6%, 488 nm at 4%, 568 nm at 3.3%. Scale bars: 10 μm and 2.5 μm (zoomed-in image). Middle: The number of ATXN7L2 puncta in hMPCs at EP (P5) and LP (P16). Right: Quantification of the fluorescence intensity of nuclear ATXN7L2 in hMPCs at EP (P5) and LP (P16). (n = 30 hMPCs). Data are shown as means ± SEM. ***$p < 0.001$ (t test).

(f) Co-immunostainig of SPIDER-β-gal and TAF5L in hMPCs at EP (P5) and LP (P16). Left: Representative images. Images were taken with ZEISS confocal LSM900, using a 63× HC Plan-Apochromat, NA 1.40 oil-immersion objective, and laser wavelengths of 405 nm at 0.6%, 488 nm at 4%, 568 nm at 3.3%. Scale bars: 10 μm and 2.5 μm (zoomed-in image). Middle: The number of TAF5L puncta in hMPCs at EP (P5) and LP (P16). Right: Quantification of the fluorescence intensity of nuclear TAF5L in hMPCs at EP (P5) and LP (P16). (n = 30 hMPCs). Data are shown as means ± SEM. **$p < 0.01$ (t test).

(g) Co-immunostaining of SPIDER-β-gal and SUPT20H in hMPCs at EP (P5) and LP (P16). Left: Representative images. Images were taken with ZEISS confocal LSM900, using a 63× HC Plan-Apochromat, NA 1.40 oil-immersion objective, and laser wavelengths of 405 nm at 0.6%, 488 nm at 4%, 568 nm at 3.3%.Scale bars: 10 μm and 2.5 μm (zoomed-in image). Middle: The number of SUPT20H puncta in hMPCs at EP (P5) and LP (P16). Right: Quantification of the fluorescence intensity of nuclear SUPT20H in hMPCs at EP

(P5) and LP (P16). (n = 30 hMPCs). Data are shown as means ± SEM. ***$p < 0.001$ (t test).

(h) Co-immunostaining of SPIDER-β-gal and SF3B3 in hMPCs at EP (P5) and LP (P16). Left: Representative images. Images were taken with ZEISS confocal LSM900, using a 63× HC Plan-Apochromat, NA 1.40 oil-immersion objective, and laser wavelengths of 405 nm at 0.6%, 488 nm at 4%, 568 nm at 3.3%. Scale bars: 10 μm and 2.5 μm (zoomed-in image). Middle: The number of SF3B3 puncta in hMPCs at EP (P5) and LP (P16). Right: Quantification of the fluorescence intensity of nuclear SF3B3 in hMPCs at EP (P5) and LP (P16). (n = 30 hMPCs). Data are shown as means ± SEM. *ns*, not significant (t test).*$p < 0.05$ (t test).

(i) Co-immunostaining of SPIDER-β-gal and ATXN7L3 in hMPCs at EP (P5) and LP (P16). Left: Representative images. Images were taken with ZEISS confocal LSM900, using a 63× HC Plan-Apochromat, NA 1.40 oil-immersion objective, and laser wavelengths of 405 nm at 0.6%, 488 nm at 4%, 568 nm at 3.3%. Scale bars: 10 μm and 2.5 μm (zoomed-in image). Middle: The number of ATXN7L3 puncta in hMPCs at EP (P5) and LP (P16). Right: Quantification of the fluorescence intensity of nuclear ATXN7L3 in hMPCs at EP (P5) and LP (P16). (n = 30 hMPCs). Data are shown as means ± SEM. ***$p < 0.001$ (t test).

Supplementary Fig. S2. **The senescent phenotypes were detected in replicatively senescent hMPCs and validation of SGF29 antibody.**

(a) SA-β-gal staining of WT, HGPS and WS hMPCs (P7). Scale bars: 20 μm. Left, representative images of SA-β-gal staining. Right, statistical analysis of the relative percentages of SA-β-gal-positive cells. Data are presented as the mean ± SEM. n = 3 biological replicates. Over 100 cells were quantified in each replicate. ***$p < 0.001$ (t test).

(b) Immunofluorescence staining of Ki67 in WT, HGPS and WS hMPCs (P7). Left, representative images of immunofluorescence staining. Images were taken with ZEISS confocal LSM900, using a 63× HC Plan-Apochromat, NA 1.40 oil-immersion objective, and laser wavelengths of 405 nm at 0.6%, 568 nm at 3.3%. Scale bars: 20 μm. Right, statistical analysis of the relative percentages of Ki67-positive cells. Data are presented as the mean ± SEM. n = 3 biological replicates. Over 100 cells were quantified in each replicate. ***$p < 0.001$ (t test).

(c) Clonal expansion assay in WT, HGPS and WS hMPCs (P7). Left, representative images of crystal violet staining. Right, quantification of the relative clonal expansion ability of WT, HGPS and WS hMPCs. Data are presented as the mean ± SEM. n = 3 biological replicates. ***$p < 0.001$ (t test).

(d) Western blotting of HP1α and $p21^{Cip1}$ in WT, HGPS and WS hMPCs (P7). Left, representative western blot images. β-Tubulin was used as a loading control. Right, statistical analysis of the relative proteins. Data are presented as the mean ± SEM. n = 3 biological replicates. **$p < 0.01$ (t test).

(e) Confocal images of HP1α and SGF29 in WT, HGPS and WS hMPCs (P7). Images were taken with ZEISS confocal LSM900, using a 63× HC Plan-Apochromat, NA 1.40 oil-immersion objective, and laser wavelengths of 405 nm at 0.6%, 488 nm at 4%, 568 nm at 3.3%. Scale bars: 10 μm and 5 μm (zoomed-in image).

(f) Validation of SGF29 antibody by immunoblotting in WT hMPCs transduced with *SGF29*-targeting sgRNAs at P5 (Passage 4) after transduction. Right, statistical analysis

of the relative proteins. Data are presented as the mean ± SEM. *n* = 3 biological replicates. ****p* < 0.001 (t test).

(g) Validation of SGF29 antibody by IF in hMPCs transduced with *SGF29*-targeting sgRNAs at P5 (Passage 4) after transduction. Images were taken with ZEISS confocal LSM900, using a 63× HC Plan-Apochromat, NA 1.40 oil-immersion objective, and laser wavelengths of 405 nm at 0.6%, 488 nm at 4%. Scale bars: 10 μm.

(h) Western blot analysis of SGF29 protein level in hMPCs transduced with lentiviruses expressing EGFP or EGFP-SGF29.

(i) Immunofluorescence staining of SGF29 in hMPCs transduced with lentiviruses expressing EGFP or EGFP-SGF29. Images were taken with ZEISS confocal LSM900, using a 63× HC Plan-Apochromat, NA 1.40 oil-immersion objective, and laser wavelengths of 405 nm at 0.6%, 488 nm at 4%, 568 nm at 3.3%. Scale bars: 10 μm and 5 μm (zoomed-in image).

(j) Western blot analysis of SGF29 in aged HGPS hMPCs after treatment with si-Control or si-SGF29. β-Tubulin was used as the loading control.

(k) SA-β-gal staining in aged HGPS hMPCs after treatment with si-Control or si-SGF29. Scale bars: 50 μm. Quantitative data on the right are presented as the mean ± SEM. *n* = 3 biological replicates. Over 100 cells were quantified in each replicate; ****p* < 0.001 (t test).

Supplementary Fig. S3. SGF29 puncta were detected in human fibroblast.

(a) Western blotting of HP1α and p21^{Cip1} in human fibroblasts at EP (P13) and LP (P23). Left, representative western blot images. β-Tubulin was used as a loading control. Right, statistical analysis the relative protein expression of HP1α and p21^{Cip1}. Data are presented as the mean ± SEM. *n* = 3 biological replicates. ****p* < 0.001(t test).

(b) SA-β-gal staining of human fibroblasts at EP (P13) and LP (P23). Left, representative images of SA-β-gal staining. Right, statistical analysis of the relative percentages of SA-β-gal-positive cells. Scale bars: 50 μm. Data are presented as the mean ± SEM. *n* = 3 biological replicates. Over 100 cells were quantified in each replicate. ****p* < 0.001 (t test).

(c) Immunofluorescence staining of Ki67 in human fibroblasts at EP (P13) and LP (P23). Left, representative images of immunofluorescence staining. Images were taken with ZEISS confocal LSM900, using a 63× HC Plan-Apochromat, NA 1.40 oil-immersion objective, and laser wavelengths of 405 nm at 0.6%, 568 nm at 3.3%. Scale bars: 20 μm. Right, statistical analysis of the relative percentages of Ki67-positive cells. Data are presented as the mean ± SEM. *n* = 3 biological replicates. Over 100 cells were quantified in each replicate. ****p* < 0.001 (t test).

(d) Clonal expansion assay in human fibroblasts at EP (P13) and LP (P23). Left, representative images of crystal violet staining. Right, quantification of the relative clonal expansion ability of EP and LP human fibroblasts. Data are presented as the mean ± SEM. *n* = 3 biological replicates. ****p* < 0.001 (t test).

(e) Confocal images of p21^{Cip1} and SGF29 in human fibroblasts at EP (P13) and LP (P23). Images were taken with ZEISS confocal LSM900, using a 63× HC Plan-Apochromat, NA 1.40 oil-immersion objective, and laser wavelengths of 405 nm at 0.6%, 488 nm at 4%, 568 nm at 3.3%. Line scans represent fluorescence intensities (FIs) along the inset arrows on the left. Scale bars: 10 μm and 5 μm (zoomed-in image).

(f) Western blot analysis of p21^{Cip1} protein level in young hFibs transduced with lentiviruses expressing either EGFP or EGFP-SGF29.The band of exogenous EGFP-SGF29 protein is marked with *.

(g) SA-β-gal staining of young hFibs transduced with lentiviruses expressing either EGFP or EGFP-SGF29. Quantitative data on the right are presented as the means ± SEM. n = 3 biological replicates; ***p < 0.001 (t test).

(h) Western blot analysis of SGF29 in aged hFibs after treatment with si-Control or si-*SGF29*. β-Tubulin was used as the loading control.

(i) SA-β-gal staining in aged hFibs after treatment with si-Control or si-SGF29. Scale bars: 50 μm. Quantitative data on the right are presented as the mean ± SEM. n = 3 biological replicates. Over 100 cells were quantified in each replicate; **p < 0.01; ***p < 0.001 (t test). Scale bars: 20 μm.

Supplementary Fig. S4. SGF29 condensates exhibit liquid-like properties in cells

(a) Western blot analysis of expressed EGFP-SGF29 and endogenous SGF29 protein levels in hMPCs and in Cynomolgus monkey testis. Top panel, anti-SGF29 antibody is used. The upper band is the expressed EGFP-SGF29, and the lower bands are endogenous SGF29. Bottom panel, anti-GAPDH antibody is used. GAPDH is used as the internal control.

(b) 3D-reconstructed representative confocal images of the hMPCs expressing EGFP-SGF29. Images were taken with ZEISS confocal LSM900, using a 63× HC Plan-Apochromat, NA 1.40 oil-immersion objective, and laser wavelengths of 488 nm at 4%. Scale bars: 10 μm and 5 μm (zoomed-in image).

(c) Live-cell images of FRAP experiments with HEK293T cells expressing EGFP-SGF29. Scale bars: 10 μm and 2 μm (zoomed-in image). Images were taken with ZEISS confocal LSM900, using a 63× HC Plan-Apochromat, NA 1.40 oil-immersion objective, and laser wavelengths of 488 nm at 4%. The curve on the right shows the relative fluorescence intensity quantified during FRAP recovery (n = 10 HEK293T cells). Photobleaching occurs at t = 1 s. Data are presented as mean ± SEM.

(d) SGF29 purified from E. coil were analysed by SDS-pAGE and visualized by coomassie blue staining.

(e) Left, representative images of purified SGF29 (2.5 μM) in vitro in the presence of total RNA, and addition of RNase A to a sample of SGF29 (2.5 μM) solubilized with 2.5 ug/ml of total RNA. Right, quantitative turbidity graphs of phase separation. Scale bars: 50 um.

Supplementary Fig. S5. Phase separation of SGF29 is not depend on the its H3K4me3 binding ability

(a) Schematic diagram of a series of EGFP-tagged SGF29 H3K4me3 interaction-disrupting truncated mutants and R207P mutant.

(b) Western blot analysis of the protein level of expressed either EGFP-SGF29-WT or R207P in hMPCs. GAPDH is used as the internal control. Control (no transfected hMPCs), * band represents endogenous SGF29.

(c) Representative time-lapse FRAP images acquired in hMPC transduced with lentiviruses expressing either EGFP-RPB1 with magnified insets showing the pre-bleach and recovery signals of EGFP-RPB1.

(d) Immunofluorescence monitored the nuclear puncta formation and the subcellular localization of EGFP-tagged SGF29 H3K4me3 interaction-disrupting truncated mutants in hMPC. Images were taken with ZEISS confocal LSM900, using a 63× HC Plan-Apochromat, NA 1.40 oil-immersion objective, and laser wavelengths of 405 nm at 0.6%, 488 nm at 4%. Scale bars: 10 μm.

Supplementary Fig. S6. Condensates formation of SGF29 is necessary for its proper promoter binding

(a) Euclidean distance heatmap showing sample repeatability of ChIP-seq data in hMPCs (P8) with SGF29 knockdown using CRISPR/Cas9, followed by expression of EGFP-SGF29-WT, D194A and R207P variants. The colour key from dark to light represents relatively near to far Euclidean distance, respectively.

(b) Bar plot showing the number of SGF29 occupancies in hMPCs (P8) with SGF29 knockdown using CRISPR/Cas9, followed by expression of EGFP-SGF29-WT, D194A and R207P variants. The SGF29 occupancies in cells expressing EGFP, EGFP-SGF29-D194A or EGFP-SGF29-R207P were considered as sequencing biases unless those co-localized with SGF29 occupancies in cells expressing EGFP-SGF29-WT.

(c) Boxplot showing the normalized ChIP-seq signals around SGF29-binding TSSs (n= 439) in hMPCs (P8) with SGF29 knockdown using CRISPR/Cas9, followed by expression of EGFP-SGF29-WT, D194A and R207P variants.

(d) Euclidean distance heatmap showing sample repeatability of RNA-seq data in hMPCs (P8) with SGF29 knockdown using CRISPR/Cas9, followed by expression of EGFP-SGF29-WT, D194A and R207P variants. The colour key from dark to light represents relatively near to far Euclidean distance, respectively.

(e) Bar plots showing the number of differentially expressed genes in hhMPCs (P8) with SGF29 knockdown using CRISPR/Cas9, followed by expression of EGFP-SGF29-WT, D194A and R207P variants.

(f) Chord diagram showing the relationship between transcriptional changes in cells expressing EGFP-SGF29-WT compared to cells expressing EGFP and transcriptional changes in cells expressing EGFP-SGF29-R207P compared to cells expressing EGFP.

(g) Chord diagram showing the relationship between transcriptional changes in cells expressing EGFP-SGF29-WT compared to cells expressing EGFP and transcriptional changes in cells expressing EGFP-SGF29-D194A compared to cells expressing EGFP.

(h) Boxplot showing the expression of these genes, which were activated in cells expressing EGFP-SGF29-WT but remained silence in cells expressing EGFP-SGF29-D194A and EGFP-SGF29-R207P, in all groups.

(i) Metaplots showing the enriched levels of SGF29 occupancies surrounding the TSS regions for genes with different indicated expression levels in hMPCs (P8) with SGF29 knockdown using CRISPR/Cas9, followed by expression of EGFP-SGF29-WT, D194A and R207P variants.

(j) Heatmap showing the enriched levels of SGF29 occupancies surrounding the TSS regions for genes, which were activated in cells expressing EGFP-SGF29-WT but remained silence in hMPCs expressing EGFP-SGF29-D194A and EGFP-SGF29-R207P, in all groups

(k) Integrative Genome Viewer tracks of the indicated ChIP-seq and RNA-seq signals at *HSPA9* locus in hMPCs (P8) with SGF29 knockdown using CRISPR/Cas9, followed by expression of EGFP-SGF29-WT, D194A and R207P variants.

Supplementary Fig. S7. Disruption of SGF29 condensates has no effect on interaction with HAT module but decreased its H3K4me3 promoter binding

(a) Detailed in formation illustrated that GCN5, ADA2A, ADA3, GTF2H2, GTF2H3, GTF2H4, SP1 and MED4 were identified as SGF29 interacting candidates by mass spectrometry. The SGF29 interacting candidates identified by mass spectrometry are listed in the Supplementary Table S2.

(b) Co-IP analysis of GCN5, ADA3 with exogenous FLAG-tagged and EGFP-tagged SGF29-WT and SGF29-R207P in hMPCs.

(c) Co-immunostaining of GCN5 and SGF29 in aged hMPCs at P16. The white arrowheads denote colocalization of SGF29 puncta with GCN5.Images were taken with ZEISS confocal LSM900, using a 63× HC Plan-Apochromat, NA 1.40 oil-immersion objective, and laser wavelengths of 405 nm at 0.6%, 488 nm at 4%, 568 nm at 3.3%. Scale bars: 10 μm and 2.5 μm (zoomed-in image).

(d) Immunofluorescence staining of GCN5 in hMPCs transduced with lentiviruses expressing either EGFP-SGF29-WT or EGFP-SGF29-R207P, EGFP-SGF29-D194A. Images were taken with ZEISS confocal LSM900, using a 63× HC Plan-Apochromat, NA 1.40 oil-immersion objective, and laser wavelengths of 405 nm at 0.6%, 488 nm at 4%, 568 nm at 3.3%. Scale bars, 10 μm and 2.5 μm (zoomed-in image).

(e) Co-IP analysis show that EGFP-tagged SGF29-WT, R207P and H3K4me3 binding mutants interact with GCN5 in the hMPCs transduced with lentiviruses expressing either EGFP, EGFP-SGF29-WT, EGFP-SGF29-R207P or EGFP-SGF29-D194A, D196A, Y245A, F264A.

(f) Global acetylation levels of H3K9, H3K18, H3K14, and H4K12 in hMPCs infected with sgNTC or sgSGF29 Lentivirus and expressed EGFP-SGF29-WT rescue, D194A and R207P. Quantified values for the H3K18 and H3K9 acetylation levels on the right are present in a bar chart with values as percentage of control (First lane). ***$p < 0.001$; *$p < 0.05$ (t test).

(g) Western blot analysis of GCN5 in aged hMPCs after treatment with si-Control or si-*GCN5*. β-Tubulin was used as the loading control.

(h) SA-β-gal staining in aged hMPCs after treatment with si-Control or si-*GCN5*. Scale bars: 50 μm. Quantitative data on the right are presented as the mean ± SEM. $n = 3$ biological replicates. Over 100 cells were quantified in each replicate; ***$p < 0.001$ (t test).

(i) Western blot analysis of GCN5 in aged hFibs after treatment with si-Control or si-*GCN5*. β-Tubulin was used as the loading control.

(j) SA-β-gal staining in aged hFibs after treatment with si-Control or si-*GCN5*. Scale bars: 50 μm. Quantitative data on the right are presented as the mean ± SEM. $n = 3$ biological

replicates. Over 100 cells were quantified in each replicate; ***$p < 0.001$; **$p < 0.01$ (t test).

Supplementary Fig. S8. Multivalent interaction of SGF29 is sensitive to condensate perturbation.

(a) Bar plot showing the phase-separation-dependent potential SGF29 interacting proteins related to RNA polymerase complex. Potential interacting proteins were ranked by PEP scores.

(b) The transcription factor prediction analysis of ageing hotspot genes, which were activated in hMPCs expressing EGFP-SGF29-WT but remained silence in hMPCs expressing EGFP-SGF29-D194A and EGFP-SGF29-R207P.

(c) Co-IP analysis show that endogenous MED4 interacts with SGF29 and SP1 proteins in hMPCs.

(d) Immunofluorescence staining of MED4 and SP1 in hMPCs transduced with lentiviruses expressing either EGFP-SGF29-WT or EGFP-SGF29-R207P.Images were taken with ZEISS confocal LSM900, using a 63× HC Plan-Apochromat, NA 1.40 oil-immersion objective, and laser wavelengths of 405 nm at 0.6%, 488 nm at 4%, 568 nm at 3.3%, and 640 nm at 2%. Scale bars: 10 μm.

(e) Immunofluorescence staining of TCOF1 in young hMPCs transduced with lentiviruses expressing either EGFP-SGF29-WT or EGFP-SGF29-R207P. Scale bars: 10 μm and 5 μm (zoomed-in image).

Supplementary Video S1. The fusion of two adjacent EGFP-SGF29 condensates in hMPCs within a 47.5-min duration. Scale bar, 10 μm.

Supplementary Video S2. Fluorescence signal recovery of EGFP-tagged SGF29 in hMPCs. A bleaching 488 nm laser pulse was applied. Scale bar, 10 μm.

Supplementary Video S3. Fluorescence signal recovery of EGFP-tagged SGF29 in HEK293T cells. A bleaching 488 nm laser pulse was applied. Scale bar, 10 μm.

Supplementary Video S4. The fusion of two adjacent SGF29 droplets in vitro. Scale bar, 50 μm.

Supplementary Table S1. IDR Score of SGF29 by PONDR (http://www.pondr.com/).

Supplementary Table S2. Interacting proteins of EGFP, EGFP-SGF29-WT and R207P identified by co-IP/MS.

Supplementary Table S3. The sequences of sgRNAs or primers used in this study for gene knockdown, overexpression, DNA FISH, RNA FISH, ChIP-qPCR, and qPCR.

Supplementary Table S4. The sequencing information of RNA-seq data, differentially expressed genes (DEGs) between hMPCs transduced with lentiviruses expressing either EGFP, EGFP-SGF29-WT, or EGFP-SGF29-R207P.

Supplementary Table S5. The sequencing information of ChIP-seq data, the genomic location of SGF29 peaks identified in hMPCs transduced with lentiviruses expressing either EGFP-SGF29-WT or EGFP-SGF29-R207P.
