# Supplementary material for: Publisher Correction: SGF29 nuclear condensates reinforce cellular aging
Source: Cell Discov. 2024 May 30;10:61. doi: 10.1038/s41421-024-00690-z (PMC11139929; doi:10.1038/s41421-024-00690-z)
Supplement: Supplementary file 1 — Supplementary Figures [file 41421_2024_690_MOESM1_ESM.pdf]

Supplementary information

Supplementary Fig. S1

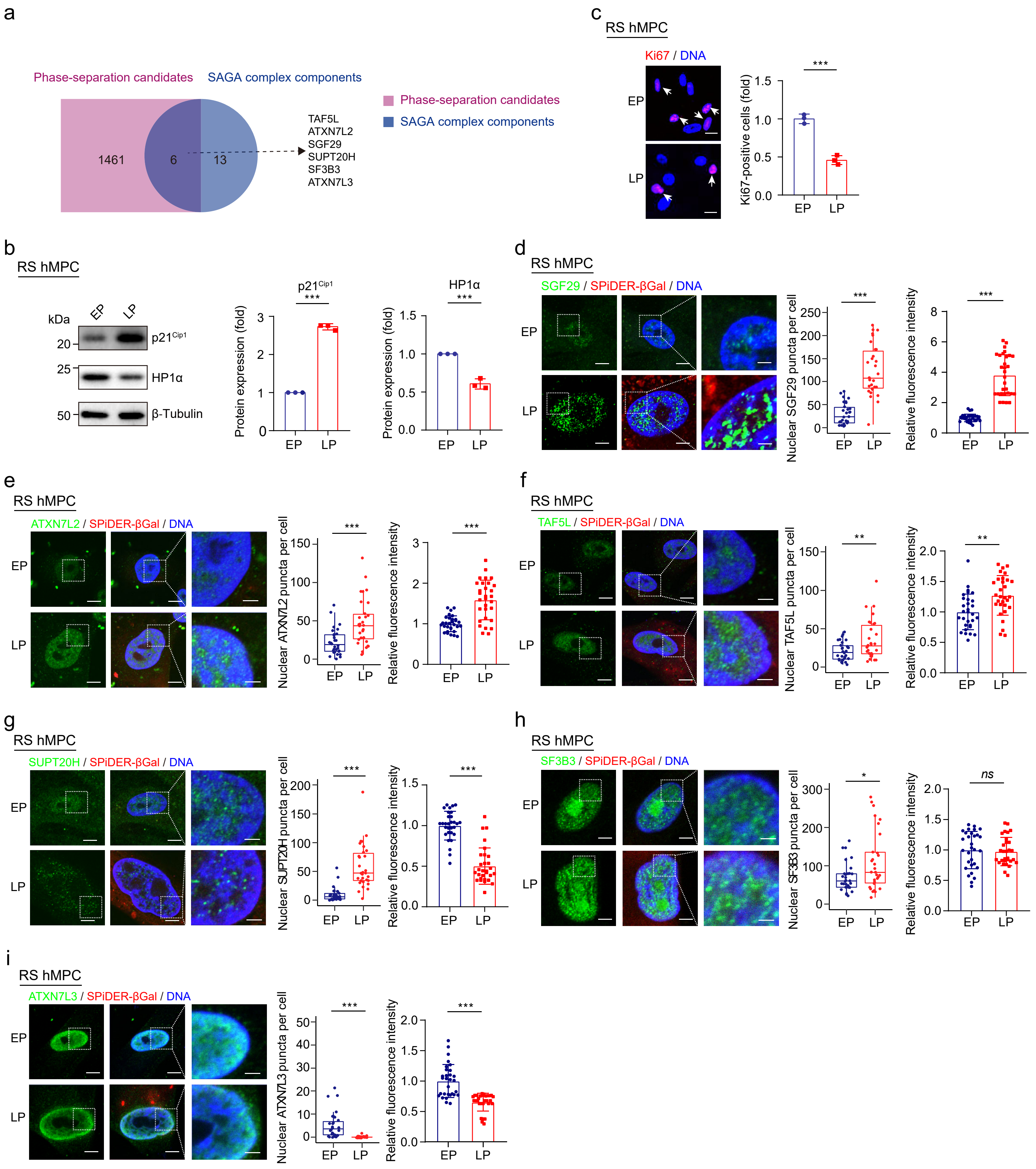

Supplementary Fig. S2

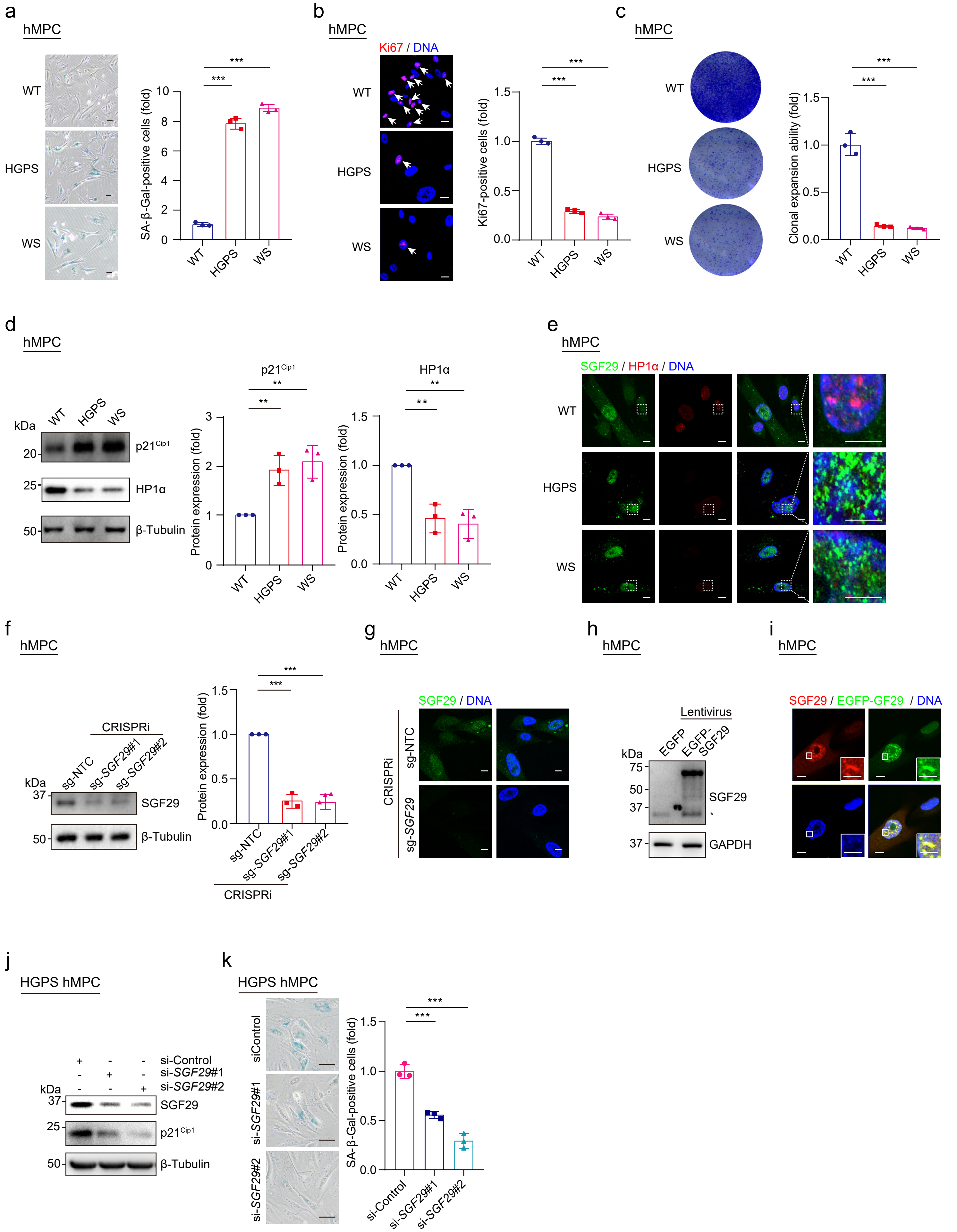

Supplementary Fig. S3

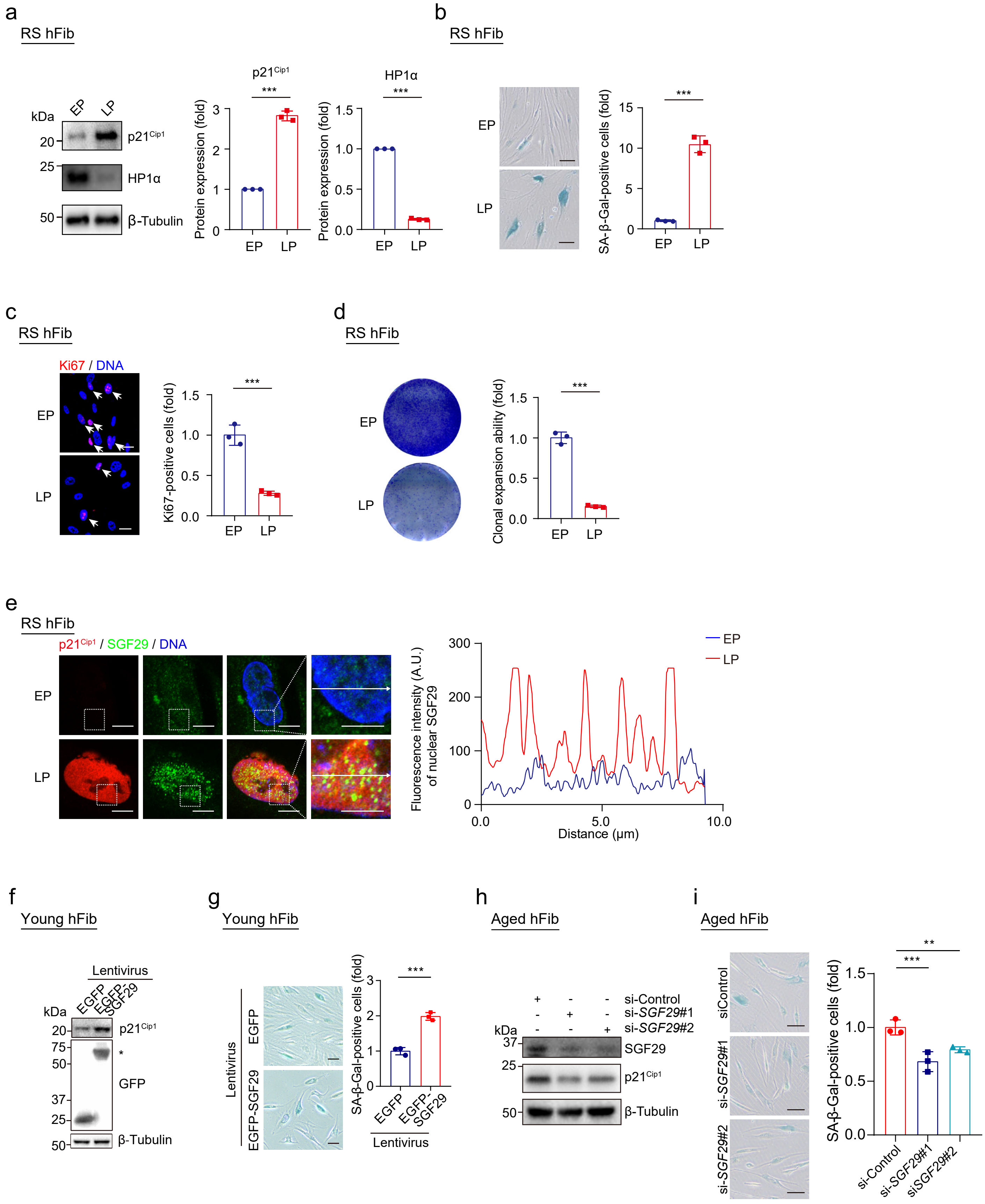

Supplementary Fig. S4

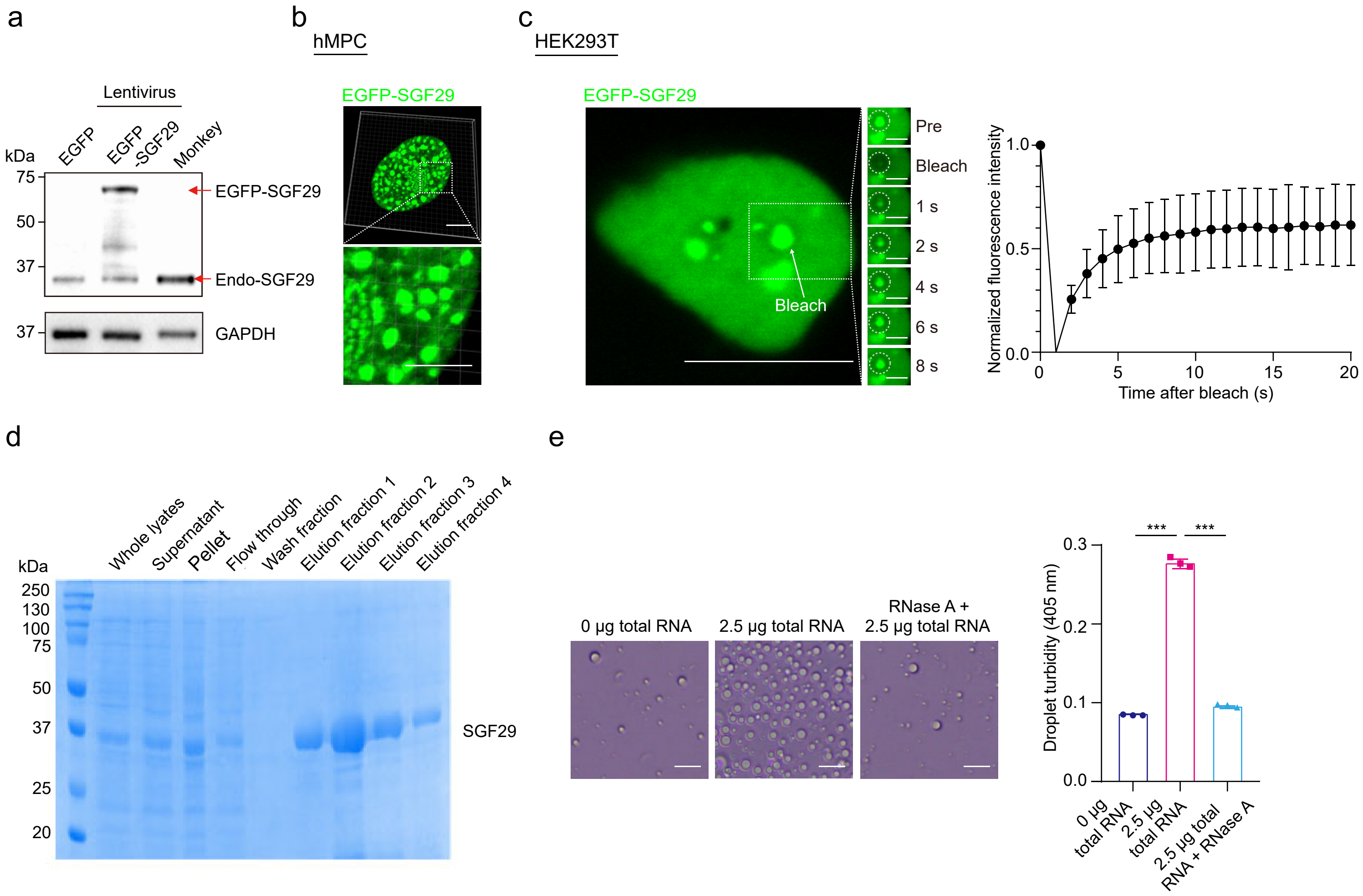

Supplementary Fig. S5

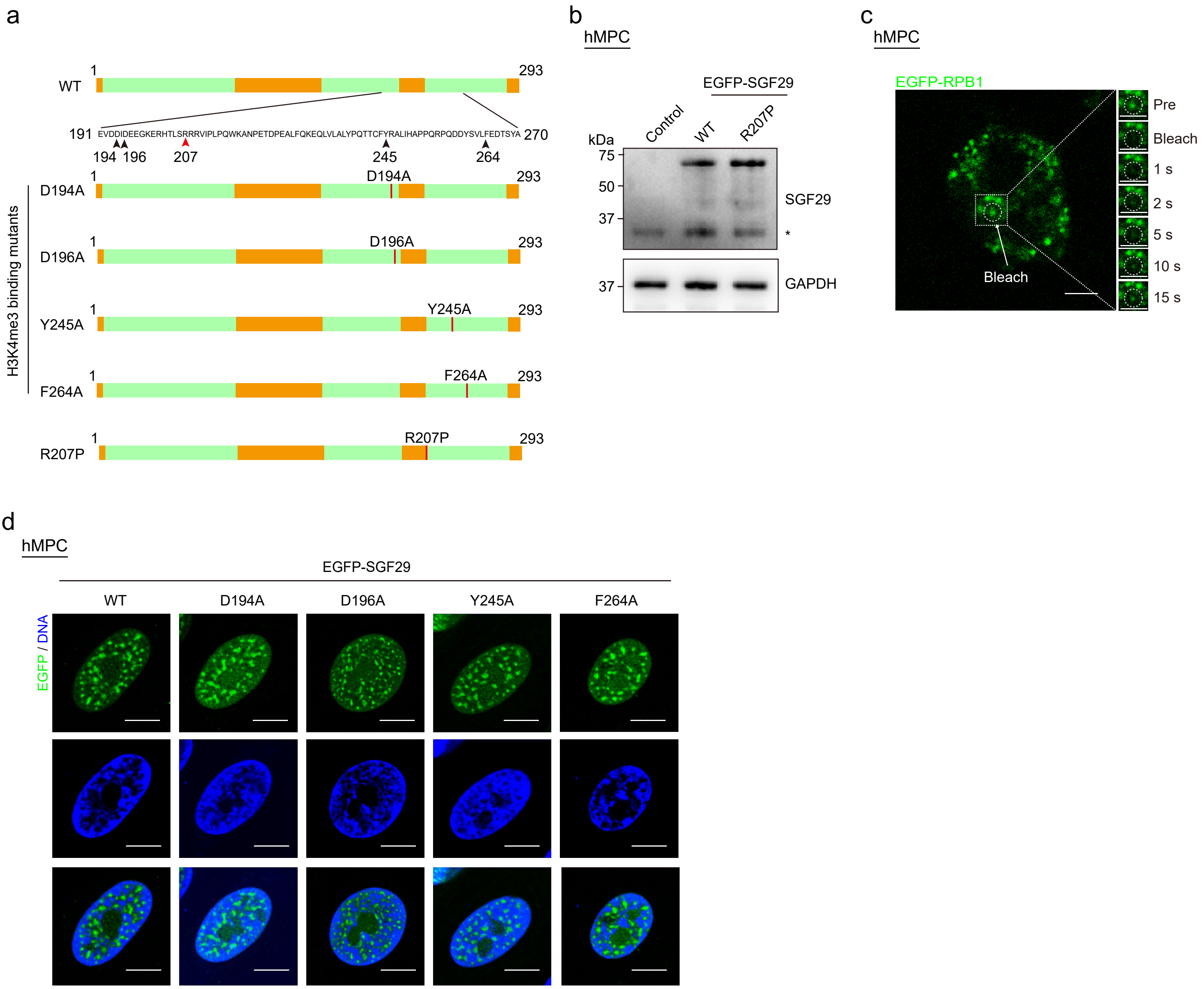

Supplementary Fig. S6

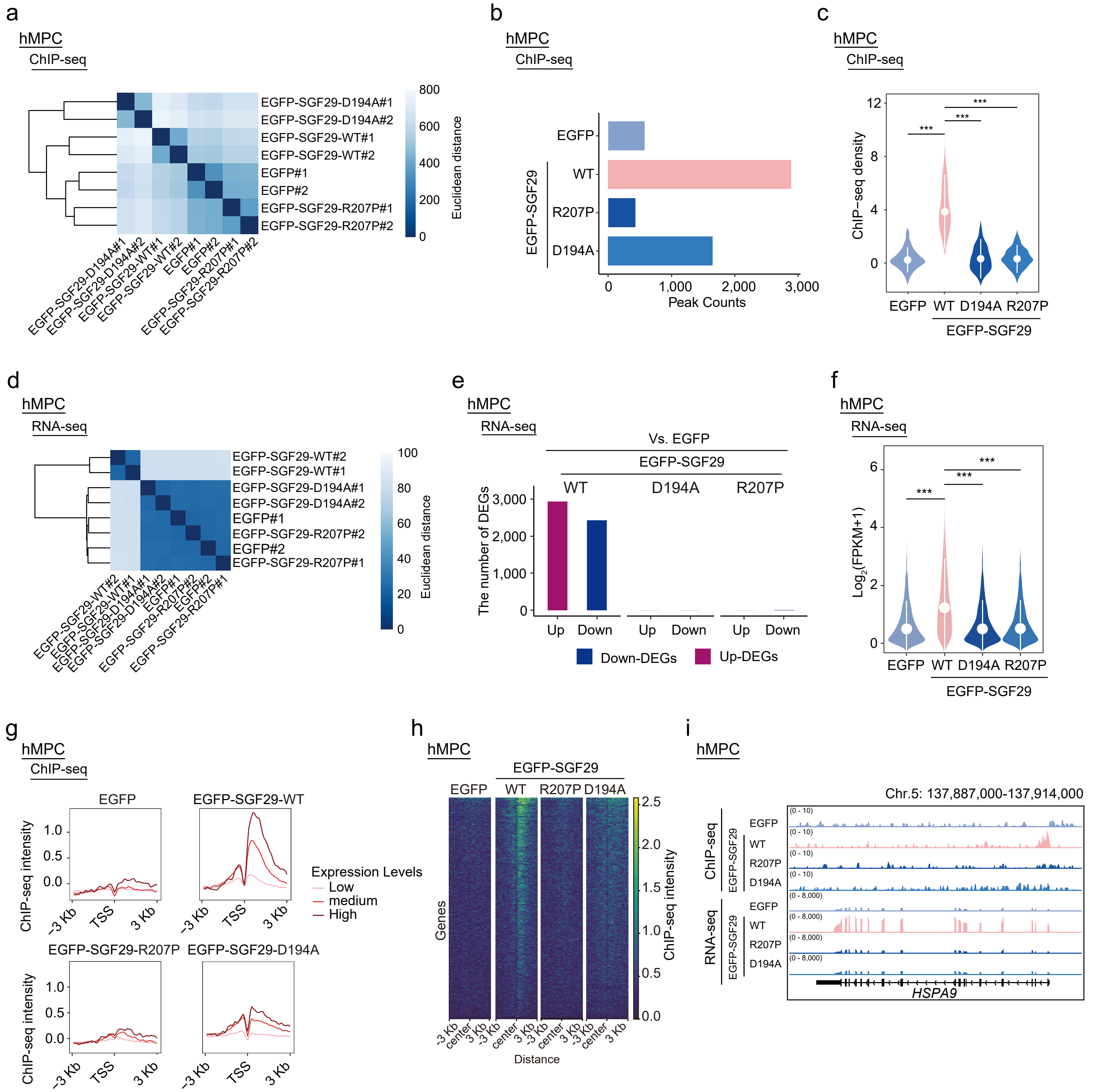

Supplementary Fig. S7

a

Type	Protein name	Coverage (%)	Unique peptide sequences
SGF29 WT and R207P shared interactors	KAT2A	22%	ELKDPDQLYTTLK<br>EYNPPDSEYCR<br>KLFVADLQR<br>LETPAQFR<br>LFVADLQR<br>LGVFSACK<br>IPYTELSHIIKK<br>MDLQQPAANLSELCR<br>NLLAQIK<br>QIPVESVPGIR<br>SCEHPLADHVSHLENVSEDEINR<br>SHPSAWPFMEPVKK<br>SQAEDVATYK<br>TLILTHFPK<br>TLPENLTLEDAKR<br>VIGGICFR<br>YETTHVFGR
	ADA2A	15%	KIYDFLIR<br>LGSFSNDPSDKPPCR<br>IVGPVEHDKFIESHALEFELR<br>LVPGAYLEYK<br>IYDFLIR<br>TAGITNFCSAR
	ADA3	14%	DCPLQFHDFK<br>SEAQHEQPEDGCPFGALTQR<br>VLEAETQILTDWQDK<br>WAQEDLLEEQKDGAR
SGF29 WT specific interactors	MED4	53%	DGEFQELMK<br>DSDIQQLQK<br>EAEQILATAVYQAK<br>ERLLSALEDLEVLSR<br>KGAISSEEIIK<br>LGGGLGVAGGNSTR<br>IHHEMQVLEK<br>IHHEMQVLEKEVEKR<br>LLQAGEENQVLELLIHR<br>LLSALEDLEVLSR<br>ISASNAVCAPLTWVPGDPR<br>RPYPTDLEMR
	GTF2H2	2%	ATIEDILFK
	GTF2H3	7%	LAVIASHIQESR
	GTF2H4	9%	LYGHPATCLAVFR
	SP1	1%	FACPECPK

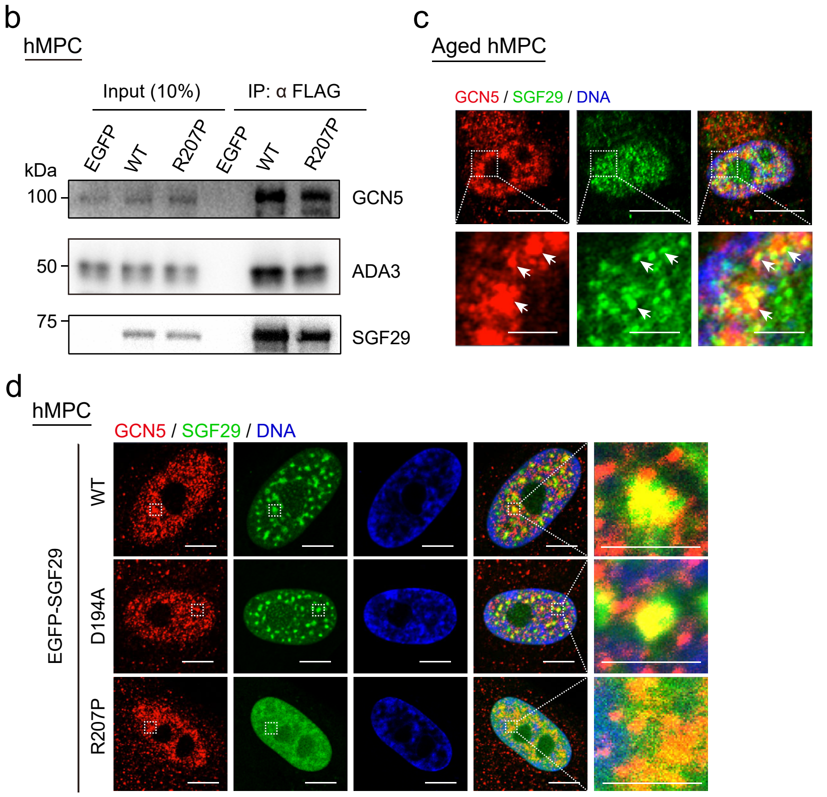

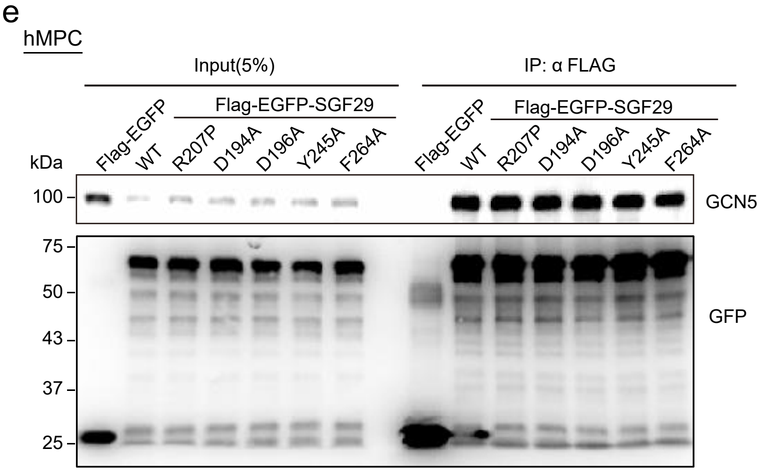

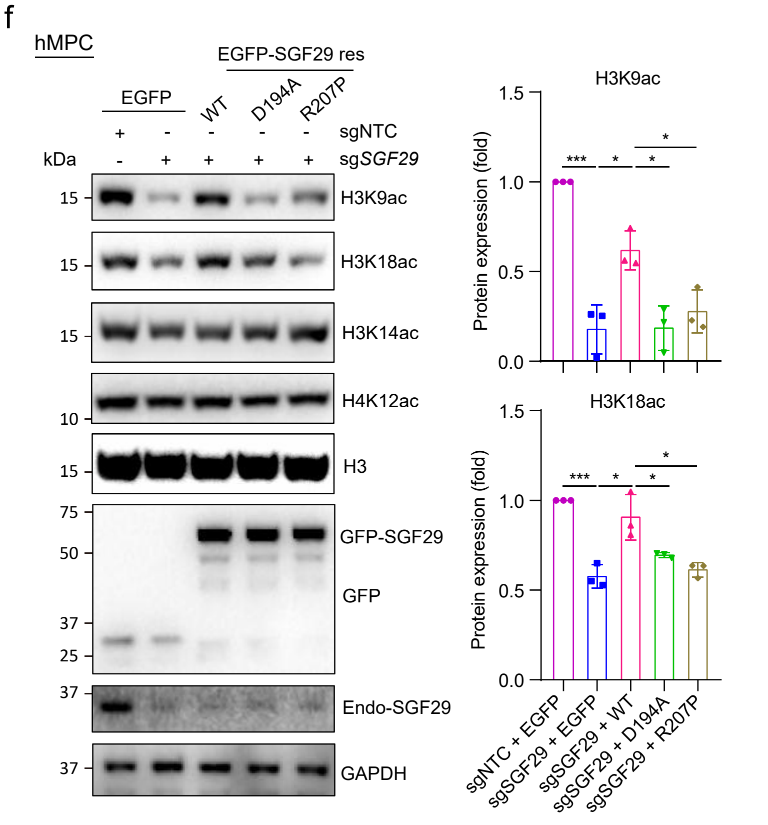

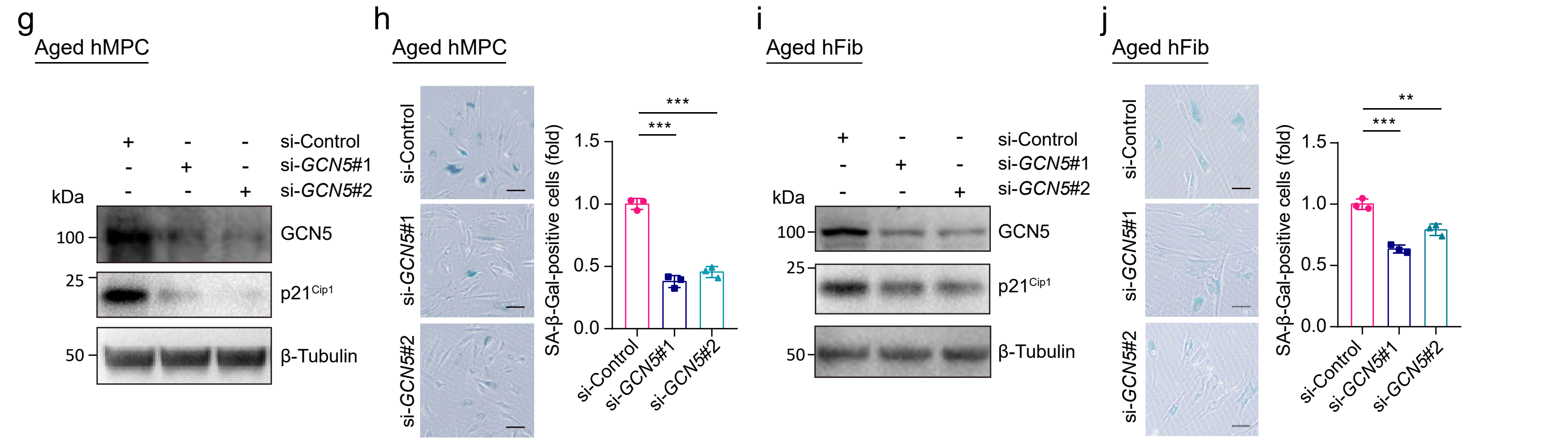

Supplementary Fig. S8

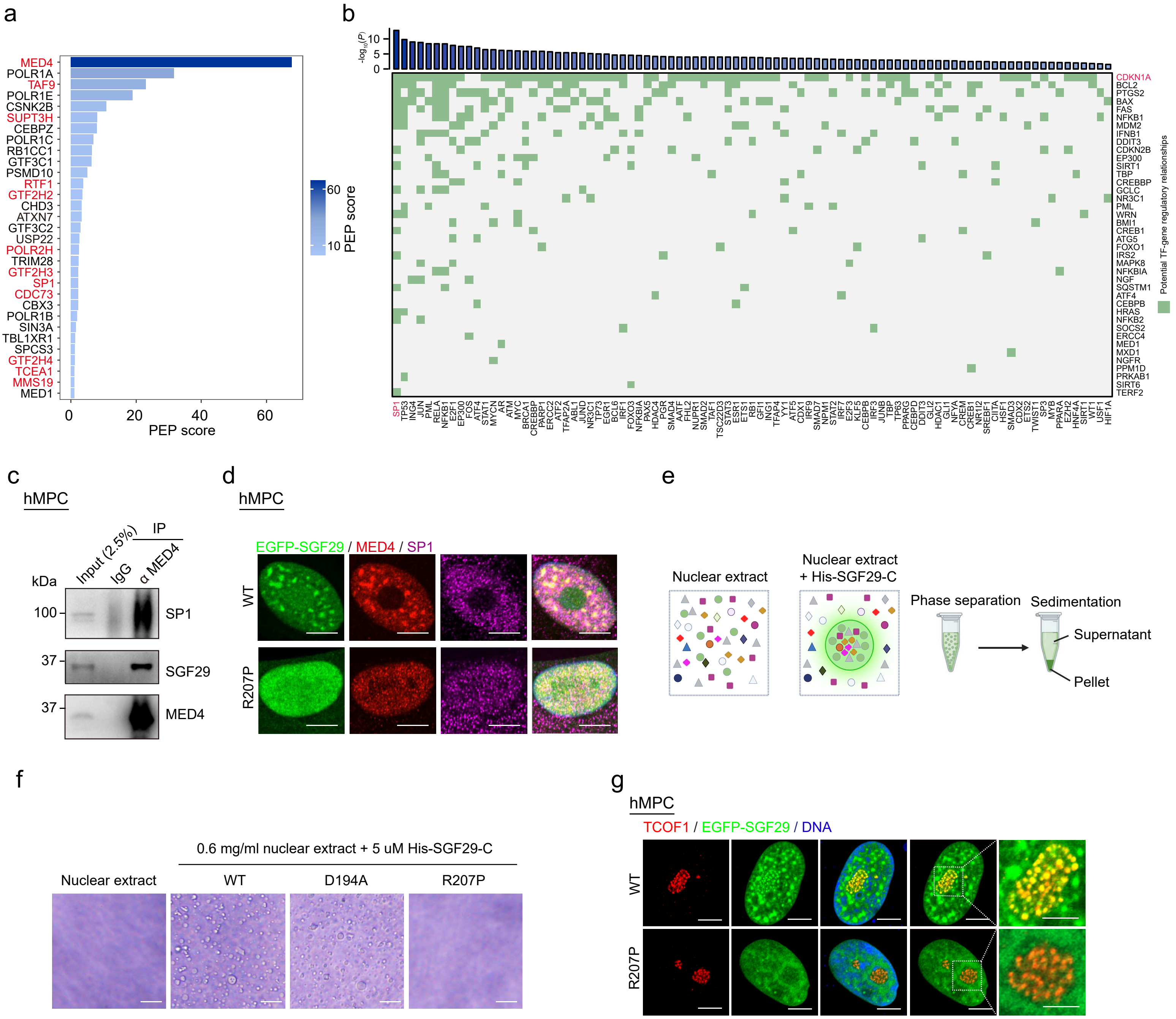

Supplementary Fig. S1. Immunofluorescence staining of SAGA complex members in hMPCs at EP (P5) and LP (P16).

(a) Venn diagram showing the members of SAGA complex with potential phase separation capabilities, including TAF5L, SUPT20H, SGF29, ATXN7L2, ATXN7L3, and SF3B3.

(b) Western blot analysis of HP1α, $p21^{Cip1}$ in WT hMPCs at EP (P5) and LP (P16). Left, representative images of western blotting. β-Tubulin was used as the loading control. Right, quantification of the relative protein levels of HP1α, $p21^{Cip1}$, and SGF29 proteins. Data are presented as the mean ± SEM. $n = 3$ biological replicates; *** $p < 0.001$ (t test).

(c) Immunofluorescence staining of Ki67 in WT hMPCs at EP (P5) and LP (P16). Left, representative images of Ki67 immunofluorescence. Scale bars, 20 μm. Right, quantification of the relative percentages of Ki67-positive cells. Data are presented as the mean ± SEM. $n = 3$ biological replicates. Over 100 cells were quantified in each replicate. *** $p < 0.001$ (t test).

(d) Immunofluorescence staining of SPiDER-βGal and SGF29 in hMPCs at EP (P5) and LP (P16). Left, representative images of SPiDER-βGal and SGF29 immunofluorescence. Scale bars, 10 μm and 2.5 μm (zoomed-in image). Middle, the number of SGF29 puncta in hMPCs at EP (P5) and LP (P16). Right, quantification of the fluorescence intensity of nuclear SGF29 in hMPCs at EP (P5) and LP (P16). $n = 30$ hMPCs. Data are shown as means ± SEM. *** $p < 0.001$ (t test).

(e) Immunofluorescence staining of SPiDER-βGal and ATXN7L2 in hMPCs at EP (P5) and LP (P16). Left, representative images of SPiDER-βGal and ATXN7L2 immunofluorescence. Scale bars, 10 μm and 2.5 μm (zoomed-in image). Middle, the number of ATXN7L2 puncta in hMPCs at EP (P5) and LP (P16). Right, quantification of the fluorescence intensity of nuclear ATXN7L2 in hMPCs at EP (P5) and LP (P16). $n = 30$ hMPCs. Data are shown as means ± SEM. *** $p < 0.001$ (t test).

(f) Immunofluorescence staining of SPiDER-βGal and TAF5L in hMPCs at EP (P5) and LP (P16). Left, representative images of SPiDER-βGal and TAF5L immunofluorescence. Scale bars, 10 μm and 2.5 μm (zoomed-in image). Middle, the number of TAF5L puncta in hMPCs at EP (P5) and LP (P16). Right, quantification of the fluorescence intensity of nuclear TAF5L in hMPCs at EP (P5) and LP (P16). $n = 30$ hMPCs. Data are shown as means ± SEM. ** $p < 0.01$ (t test).

(g) Immunofluorescence staining of SPiDER-βGal and SUPT20H in hMPCs at EP (P5) and LP (P16). Left, representative images of SPiDER-βGal and SUPT20H immunofluorescence. Scale bars, 10 μm and 2.5 μm (zoomed-in image). Middle, the number of SUPT20H puncta in hMPCs at EP (P5) and LP (P16). Right, quantification of the fluorescence intensity of nuclear SUPT20H in hMPCs at EP (P5) and LP (P16). $n = 30$ hMPCs. Data are shown as means ± SEM. *** $p < 0.001$ (t test).

(h) Immunofluorescence staining of SPiDER-βGal and SF3B3 in hMPCs at EP (P5) and LP (P16). Left, representative images of SPiDER-βGal and SF3B3 immunofluorescence. Scale bars, 10 μm and 2.5 μm (zoomed-in image). Middle, the number of SF3B3 puncta in hMPCs at EP (P5) and LP (P16). Right, quantification of the fluorescence intensity of nuclear SF3B3 in hMPCs at EP (P5) and LP (P16). $n = 30$ hMPCs. Data are shown as means ± SEM. *ns*, not significant (t test). * $p < 0.05$ (t test).

(i) Immunofluorescence staining of SPiDER-βGal and ATXN7L3 in hMPCs at EP (P5) and LP (P16). Left, representative images of SPiDER-βGal and ATXN7L3

immunofluorescence. Scale bars, 10 μm and 2.5 μm (zoomed-in image). Middle, the number of ATXN7L3 puncta in hMPCs at EP (P5) and LP (P16). Right, quantification of the fluorescence intensity of nuclear ATXN7L3 in hMPCs at EP (P5) and LP (P16). $n = 30$ hMPCs. Data are shown as means ± SEM. ***$p < 0.001$ (*t* test).

Supplementary Fig. S2. The senescent phenotypes were detected in replicatively senescent hMPCs and validation of SGF29 antibody.

(a) SA-β-Gal staining of WT, HGPS and WS hMPCs (P7). Left, representative images of SA-β-Gal staining. Scale bars, 20 μm. Right, quantification of the relative percentages of SA-β-Gal-positive cells. Data are presented as the mean ± SEM. $n = 3$ biological replicates. Over 100 cells were quantified in each replicate. ***$p < 0.001$ (*t* test).

(b) Immunofluorescence staining of Ki67 in WT, HGPS and WS hMPCs (P9). Left, representative images of Ki67 immunofluorescence. Scale bars, 20 μm. Right, quantification of the relative percentages of Ki67-positive cells. Data are presented as the mean ± SEM. $n = 3$ biological replicates. Over 100 cells were quantified in each replicate. ***$p < 0.001$ (*t* test).

(c) Clonal expansion assay in WT, HGPS and WS hMPCs (P9). Left, representative images of crystal violet staining. Right, quantification of the relative clonal expansion ability of WT, HGPS and WS hMPCs. Data are presented as the mean ± SEM. $n = 3$ biological replicates. ***$p < 0.001$ (*t* test).

(d) Western blot analysis of HP1α and $p21^{Cip1}$ in WT, HGPS and WS hMPCs (P9). Left, representative images of western blotting. β-Tubulin was used as the loading control. Right, quantification of the relative protein levels of HP1α, $p21^{Cip1}$, and SGF29 proteins. Data are presented as the mean ± SEM. $n = 3$ biological replicates. **$p < 0.01$ (*t* test).

(e) Immunofluorescence staining of HP1α and SGF29 in WT, HGPS and WS hMPCs (P9). Scale bars, 10 μm and 5 μm (zoomed-in image).

(f) Validation of SGF29 antibody by immunoblotting in WT hMPCs transduced with *SGF29*-targeting sgRNAs at P5 (Passage 3) after transduction. Left, representative images of western blotting. Right, quantification of protein levels of SGF29. Data are presented as the mean ± SEM. $n = 3$ biological replicates. ***$p < 0.001$ (*t* test).

(g) Validation of SGF29 antibody by Immunofluorescence in hMPCs transduced with *SGF29*-targeting sgRNAs at P5 (Passage 3) after transduction. Scale bars, 10 μm.

(h) Western blot analysis of SGF29 in hMPCs transduced with lentiviruses expressing EGFP or EGFP-SGF29.

(i) Immunofluorescence staining of SGF29 in hMPCs transduced with lentiviruses expressing EGFP or EGFP-SGF29. Scale bars, 10 μm and 5 μm (zoomed-in image).

(j) Western blot analysis of SGF29 in late passage HGPS hMPCs (P9, aged HGPS hMPCs) after treatment with si-Control or si-*SGF29*. β-Tubulin was used as the loading control.

(k) SA-β-Gal staining of late passage HGPS hMPCs (P9, aged HGPS hMPCs) after treatment with si-Control or si-*SGF29*. Scale bars, 50 μm. Left, representative images of SA-β-Gal staining. Right, quantification of the relative percentages of SA-β-Gal-positive cells. Data are presented as the mean ± SEM. $n = 3$ biological replicates. Over 100 cells were quantified in each replicate; ***$p < 0.001$ (*t* test).

Supplementary Fig. S3. SGF29 puncta were detected in human fibroblast.

(a) Western blot analysis of HP1α and p21^{Cip1} in human fibroblasts at EP (P13) and LP (P23). Left, representative images of western blotting. β-Tubulin was used as the loading control. Right, quantification of the relative protein levels of HP1α and p21^{Cip1}. Data are presented as the mean ± SEM. n = 3 biological replicates. ***p < 0.001(t test).

(b) SA-β-Gal staining of human fibroblasts at EP (P13) and LP (P23). Left, representative images of SA-β-Gal staining. Scale bars, 50 μm. Right, quantification of the relative percentages of SA-β-Gal-positive cells. Data are presented as the mean ± SEM. n = 3 biological replicates. Over 100 cells were quantified in each replicate. ***p < 0.001 (t test).

(c) Immunofluorescence staining of Ki67 in human fibroblasts at EP (P13) and LP (P23). Left, representative images of Ki67 immunofluorescence. Scale bars, 20 μm. Right, quantification of the relative percentages of Ki67-positive cells. Data are presented as the mean ± SEM. n = 3 biological replicates. Over 100 cells were quantified in each replicate. ***p < 0.001 (t test).

(d) Clonal expansion assay in human fibroblasts at EP (P13) and LP (P23). Left, representative images of crystal violet staining. Right, quantification of the relative clonal expansion ability of EP and LP human fibroblasts. Data are presented as the mean ± SEM. n = 3 biological replicates. ***p < 0.001 (t test).

(e) Immunofluorescence staining of p21^{Cip1} and SGF29 in human fibroblasts at EP (P13) and LP (P23). Left, representative images of p21^{Cip1} and SGF29 staining. Right, quantification of the fluorescence intensity along the line embedded in the zoomed-in images following the arrow direction. Scale bars, 10 μm and 5 μm (zoomed-in image).

(f) Western blot analysis of p21^{Cip1} protein level in early passage human fibroblasts (P13, young human fibroblasts) transduced with lentiviruses expressing either EGFP or EGFP-SGF29.The band of exogenous EGFP-SGF29 protein is marked with *.

(g) SA-β-Gal staining of early passage human fibroblasts (P13, young human fibroblasts) transduced with lentiviruses expressing either EGFP or EGFP-SGF29. Left, representative images of SA-β-Gal staining. Scale bars, 20 μm. Right, quantification of the relative percentages of SA-β-Gal-positive cells. Data are presented as the means ± SEM. n = 3 biological replicates; ***p < 0.001 (t test).

(h) Western blot analysis of SGF29 in late passage human fibroblasts (P23, aged human fibroblasts) after treatment with si-Control or si-*SGF29*. β-Tubulin was used as the loading control.

(i) SA-β-Gal staining of late passage human fibroblasts (P23, aged human fibroblasts) after treatment with si-Control or si-*SGF29*. Left, representative images of SA-β-Gal staining. Scale bars, 50 μm. Right, quantification of the relative percentages of SA-β-Gal-positive cells. Data are presented as the mean ± SEM. n = 3 biological replicates. Over 100 cells were quantified in each replicate; **p < 0.01; ***p < 0.001 (t test).

Supplementary Fig. S4. SGF29 condensates exhibit liquid-like properties in cells

(a) Western blot analysis of expressed EGFP-SGF29 and endogenous SGF29 protein levels in hMPCs and in cynomolgus monkey testis. Top panel, anti-SGF29 antibody was used. The upper band is the expressed EGFP-SGF29, and the lower bands are endogenous SGF29. Bottom panel, anti-GAPDH antibody was used. GAPDH is used as the internal control.

(b) 3D-reconstructed representative confocal images of the hMPCs expressing EGFP-SGF29. Scale bars, 10 μm and 5 μm (zoomed-in image).

(c) Live-cell images of fluorescence recovery after photobleaching (FRAP) experiments in HEK293T cells expressing EGFP-SGF29. Left, representative time-lapse FRAP images of EGFP-SGF29. Scale bars, 10 μm and 2 μm (zoomed-in image). Right, quantification of fluorescence intensity during FRAP assay. Data are presented as mean ± SEM. n = 10 HEK293T cells. Photobleaching occurs at t = 1 s.

(d) Purified recombinant His-SGF29 protein were analysed by SDS-pAGE and visualized by coomassie blue staining.

(e) RNA regulates the phase behavior of SGF29. Left, representative images of purified SGF29 (2.5 μM) in the presence of total RNA, and addition of RNase A to a sample of SGF29 (2.5 μM) solubilized with 2.5 ug/ml of total RNA. Right, quantitative turbidity graphs of droplets. n = 3 biological replicates. ***p < 0.001 (t test). Scale bars, 50 μm.

Supplementary Fig. S5. Phase separation of SGF29 is not depend on the its H3K4me3 binding ability

(a) Schematic diagram of a series of EGFP-tagged SGF29 H3K4me3 interaction-disrupting truncated mutants and R207P mutant.

(b) Western blot analysis of SGF29 in hMPCs transduced with lentiviruses expressing either EGFP-SGF29-WT (WT) and EGFP-SGF29-R207P (R207P). GAPDH was used as the internal control. Control (no transfected hMPCs), * band represents endogenous SGF29.

(c) Representative time-lapse FRAP images of EGFP-RPB1 in hMPCs transduced with lentiviruses expressing EGFP-RPB1. Scale bars, 5 μm and 1 μm (zoomed-in image).

(d) Immunofluorescence showed the nuclear puncta formation of EGFP-tagged SGF29 H3K4me3 interaction-disrupting truncated mutants in hMPC transduced with lentiviruses expressing EGFP-SGF29-WT (WT), EGFP-SGF29-D194A (D194A), EGFP-SGF29-D196A (D196A), EGFP-SGF29-Y245A (Y245A) or EGFP-SGF29-F264A (F264A) variants, respectively.

Supplementary Fig. S6. Condensates formation of SGF29 is necessary for its proper promoter binding

(a) Euclidean distance heatmap showing sample repeatability of ChIP-seq data in hMPCs (P13) with CRISPR/Cas9-mediated knockdown of endogenous SGF29 followed by overexpression of EGFP, EGFP-SGF29-WT (WT), EGFP-SGF29-D194A (D194A) or EGFP-SGF29-R207P (R207P) variants, respectively. The colour key from dark to light represents relatively near to far Euclidean distance.

(b) Bar plot showing the number of SGF29 occupancies in hMPCs (P13) with CRISPR/Cas9-mediated knockdown of endogenous SGF29 followed by overexpression of EGFP, EGFP-SGF29-WT (WT), EGFP-SGF29-D194A (D194A) or EGFP-SGF29-R207P (R207P) variants, respectively.

(c) Boxplot showing the normalized ChIP-seq signals around SGF29-binding TSSs (n= 439) in hMPCs (P13) with CRISPR/Cas9-mediated knockdown of endogenous SGF29 followed by overexpression of EGFP, EGFP-SGF29-WT (WT), EGFP-SGF29-D194A (D194A) or EGFP-SGF29-R207P (R207P) variants, respectively.

(d) Euclidean distance heatmap showing sample repeatability of RNA-seq data in hMPCs (P13) with CRISPR/Cas9-mediated knockdown of endogenous SGF29 followed by overexpression of EGFP, EGFP-SGF29-WT (WT), EGFP-SGF29-D194A (D194A) or EGFP-SGF29-R207P (R207P) variants, respectively. The colour key from dark to light represents relatively near to far Euclidean distance.

(e) Bar plots showing the number of differentially expressed genes in hMPCs (P13) with CRISPR/Cas9-mediated knockdown of endogenous SGF29 followed by overexpression of EGFP-SGF29-WT (WT), EGFP-SGF29-D194A (D194A) or EGFP-SGF29-R207P (R207P) variants compared to hMPCs with overexpression of EGFP.

(f) Boxplot showing the expression levels of these genes, which were activated in hMPCs expressing EGFP-SGF29-WT (WT) but remained silence in hMPCs expressing EGFP-SGF29-D194A (D194A) or EGFP-SGF29-R207P (R207P) mutants, in all groups.

(g) Metaplots showing the enriched levels of SGF29 occupancies surrounding the TSS regions for genes with indicated expression levels in hMPCs (P13) with CRISPR/Cas9-mediated knockdown of endogenous SGF29 followed by overexpression of EGFP, EGFP-SGF29-WT (WT), EGFP-SGF29-D194A (D194A) or EGFP-SGF29-R207P (R207P) variants, respectively.

(h) Heatmap showing the enriched levels of SGF29 occupancies surrounding the TSS regions for genes, which were activated in hMPCs expressing EGFP-SGF29-WT (WT) but remained silence in hMPCs expressing EGFP-SGF29-D194A (D194A) and EGFP-SGF29-R207P (R207P) variants, in all groups.

(i) Integrative Genome Viewer tracks of the indicated ChIP-seq and RNA-seq signals at *HSPA9* locus in hMPCs (P13) with CRISPR/Cas9-mediated knockdown of endogenous SGF29 followed by overexpression of EGFP, EGFP-SGF29-WT (WT), and EGFP-SGF29-D194A (D194A) or EGFP-SGF29-R207P (R207P) variants, respectively.

Supplementary Fig. S7. Disruption of SGF29 condensates has no effect on interaction with HAT module but decreased its H3K4me3 promoter binding

(a) Detailed information of representative SGF29 interacting candidates identified by mass spectrometry, including GCN5, ADA2A, ADA3, GTF2H2, GTF2H3, GTF2H4, SP1 and MED4. The SGF29 interacting candidates identified by mass spectrometry are listed in the Supplementary Table S2.

(b) Co-IP analysis showing the interactions between indicated proteins and EGFP-SGF29-WT (WT) and EGFP-SGF29-R207P (R207P) in hMPCs.

(c) Immunofluorescence staining of GCN5 and SGF29 in late passage WT hMPCs (P16, aged hMPCs). The white arrowheads denote colocalization of SGF29 puncta with GCN5. Scale bars, 10 μm and 2.5 μm (zoomed-in image).

(d) Immunofluorescence staining of GCN5 in hMPCs transduced with lentiviruses expressing either EGFP-SGF29-WT (WT), and EGFP-SGF29-D194A (D194A) or EGFP-SGF29-R207P (R207P) variants. Scale bars, 10 μm and 2.5 μm (zoomed-in image).

(e) Co-IP analysis showing the interactions between GCN5 and EGFP-SGF29-WT (WT), EGFP-SGF29-R207P (R207P) or EGFP-tagged SGF29 H3K4me3 interaction-disrupting truncated mutants in hMPCs.

(f) Global acetylation levels of H3K9, H3K18, H3K14, and H4K12 in hMPCs with CRISPR/Cas9-mediated knockdown of endogenous SGF29 followed by overexpression of EGFP, EGFP-SGF29-WT rescue (WT), EGFP-SGF29-D194A rescue (D194A) or EGFP-SGF29-R207P rescue (R207P) variants, respectively. Left, representative images of western blotting. GAPDH was used as the loading control. Right, quantification of the relative acetylation levels of H3K18 and H3K9. ***$p < 0.001$; *$p < 0.05$ (*t* test).

(g) Western blot analysis of GCN5 in late passage WT hMPCs (P16, aged hMPCs) after treatment with si-Control or si-*GCN5*. β-Tubulin was used as the loading control.

(h) SA-β-Gal staining of late passage WT hMPCs (P16, aged hMPCs) after treatment with si-Control or si-*GCN5*. Left, representative images of SA-β-Gal staining. Scale bars, 50 μm. Right, quantification of the relative percentages of SA-β-Gal-positive cells. Data are presented as the mean ± SEM. $n = 3$ biological replicates. Over 100 cells were quantified in each replicate; ***$p < 0.001$ (*t* test).

(i) Western blot analysis of GCN5 in late passage human fibroblasts (P23, aged human fibroblasts) after treatment with si-Control or si-*GCN5*. β-Tubulin was used as the loading control.

(j) SA-β-Gal staining of late passage human fibroblasts (P23, aged human fibroblasts) after treatment with si-Control or si-*GCN5*. Left, representative images of SA-β-Gal staining. Scale bars, 50 μm. Right, quantification of the relative percentages of SA-β-Gal-positive cells. Data are presented as the mean ± SEM. $n = 3$ biological replicates. Over 100 cells were quantified in each replicate; ***$p < 0.001$; **$p < 0.01$ (*t* test).

Supplementary Fig. S8. Multivalent interaction of SGF29 is sensitive to condensate perturbation.

(a) Bar plot showing the phase-separation-dependent SGF29 interacting proteins related to RNA polymerase complex. SGF29 interacting proteins were ranked by peptide posterior error probability (PEP) scores.

(b) The transcription factor prediction analysis of ageing hotspot genes, which were activated in hMPCs expressing EGFP-SGF29-WT (WT) but remained silence in hMPCs expressing EGFP-SGF29-D194A (D194A) and EGFP-SGF29-R207P (R207P) variants.

(c) Co-IP analysis showing the interactions between endogenous MED4 and SGF29 and SP1 in hMPCs.

(d) Immunofluorescence staining of MED4 and SP1 in hMPCs transduced with lentiviruses expressing either EGFP-SGF29-WT (WT) or EGFP-SGF29-R207P (R207P). Scale bars, 10 μm.

(e) Schematic diagram of pelleting experiment.

(f) Purified recombinant SGF29-C(54-293)-WT and SGF29-C(54-293)-D194A forms droplets in the nuclear extract. Scale bars, 50 μm.

(g) Immunofluorescence staining of TCOF1 in early passage WT hMPCs (P5, young hMPCs) transduced with lentiviruses expressing either EGFP-SGF29-WT (WT) or EGFP-SGF29-R207P (R207P). Scale bars, 10 μm and 5 μm (zoomed-in image).

Supplementary Video S1. The fusion of two adjacent EGFP-SGF29 condensates in hMPCs within a 47.5-min duration. Scale bar, 10 μm.

Supplementary Video S2. Fluorescence signal recovery of EGFP-SGF29 in hMPCs. A bleaching 488 nm laser pulse was applied. Scale bar, 10 μm.

Supplementary Video S3. Fluorescence signal recovery of EGFP-SGF29 in HEK293T cells. A bleaching 488 nm laser pulse was applied. Scale bar, 10 μm.

Supplementary Video S4. The fusion of two adjacent SGF29 droplets in vitro. Scale bar, 50 μm.

Supplementary Table S1. IDR Score of SGF29 by PONDR (http://www.pondr.com/).

Supplementary Table S2. Interacting proteins of EGFP, EGFP-SGF29-WT and R207P identified by Co-IP/MS.

Supplementary Table S3. The sequences of sgRNAs or primers used in this study for gene knockdown, overexpression, DNA FISH, RNA FISH, ChIP-qPCR, and qPCR.

Supplementary Table S4. The sequencing information of RNA-seq data, differentially expressed genes (DEGs) in hMPCs (P13) with CRISPR/Cas9-mediated knockdown of endogenous SGF29 followed by overexpression of EGFP-SGF29-WT, EGFP-SGF29-D194A (D194A) or EGFP-SGF29-R207P (R207P) variants compared to hMPCs with overexpression of EGFP.

Supplementary Table S5. The sequencing information of ChIP-seq data, the genomic location of SGF29 peaks identified in hMPCs (P13) with CRISPR/Cas9-mediated knockdown of endogenous SGF29 followed by overexpression of EGFP-SGF29-WT (WT), EGFP-SGF29-D194A (D194A) or EGFP-SGF29-R207P (R207P) variants.
